# SIMplyBee: an R package to simulate honeybee populations and breeding programs

**DOI:** 10.1186/s12711-023-00798-y

**Published:** 2023-05-09

**Authors:** Jana Obšteter, Laura K. Strachan, Jernej Bubnič, Janez Prešern, Gregor Gorjanc

**Affiliations:** 1 Department of Animal Science, The Agricultural Institute of Slovenia, Ljubljana, Slovenia; 2 The Roslin Institute and Royal (Dick) School of Veterinary Medicine, The University of Edinburgh, Edinburgh, UK; 3 Biotechnical Faculty, Department of Animal Science, The University of Ljubljana, Ljubljana, Slovenia

## Abstract

**Background:**

The Western honeybee is an economically important species globally, but has been experiencing colony losses that lead to economical damage and decreased genetic variability. This situation is spurring additional interest in honeybee breeding and conservation programs. Stochastic simulators are essential tools for rapid and low-cost testing of breeding programs and methods, yet no existing simulator allows for a detailed simulation of honeybee populations. Here we describe SIMplyBee, a holistic simulator of honeybee populations and breeding programs. SIMplyBee is an R package and hence freely available for installation from CRAN http://cran.r-project.org/package=SIMplyBee.

**Implementation:**

SIMplyBee builds upon the stochastic simulator AlphaSimR that simulates individuals with their corresponding genomes and quantitative genetic values. To enable honeybee-specific simulations, we extended AlphaSimR by developing classes for global simulation parameters, SimParamBee, for a honeybee colony, Colony, and multiple colonies, MultiColony. We also developed functions to address major honeybee specificities: honeybee genome, haplodiploid inheritance, social organisation, complementary sex determination, polyandry, colony events, and quantitative genetics at the individual- and colony-levels.

**Results:**

We describe its implementation for simulating a honeybee genome, creating a honeybee colony and its members, addressing haplodiploid inheritance and complementary sex determination, simulating colony events, creating and managing multiple colonies at the same time, and obtaining genomic data and honeybee quantitative genetics. Further documentation, available at http://www.SIMplyBee.info, provides details on these operations and describes additional operations related to genomics, quantitative genetics, and other functionalities.

**Discussion:**

SIMplyBee is a holistic simulator of honeybee populations and breeding programs. It simulates individual honeybees with their genomes, colonies with colony events, and individual- and colony-level genetic and breeding values. Regarding the latter, SIMplyBee takes a user-defined function to combine individual- into colony-level values and hence allows for modeling any type of interaction within a colony. SIMplyBee provides a research platform for testing breeding and conservation strategies and their effect on future genetic gain and genetic variability. Future developments of SIMplyBee will focus on improving the simulation of honeybee genomes, optimizing the simulator’s performance, and including spatial awareness in mating functions and phenotype simulation. We invite the honeybee genetics and breeding community to join us in the future development of SIMplyBee.

**Supplementary Information:**

The online version contains supplementary material available at 10.1186/s12711-023-00798-y.

## Background

The Western honeybee (*Apis mellifera*) is an economically important species globally that plays a major role in pollination and food production. The value of insect pollinators is estimated at 150 billion euros per year worldwide, which is approximately 10 percent of the global agriculture production [1–3]. In recent decades, wild and managed honeybee populations have been experiencing increased colony losses due to numerous biotic and abiotic factors [4–6]. Besides the economic loss, high colony mortality and human-mediated hybridisation have also driven the loss of within-species diversity during the last century and put native subspecies at risk [5, 7–9]. Although honeybees are a diverse species that are differentiated into seven evolutionary lineages and 33 subspecies [10, 11], two subspecies, *A. m. ligustica* and *A. m. carnica*, dominate the vast majority of commercial beekeeping operations [12]. The loss of genetic variability can decrease the fitness of the populations and further increases the susceptibility of populations to ecological and anthropogenic factors [5, 9].

Due to increased colony losses and a decline in genetic diversity, there has been increasing interest in honeybee management programs, either for breeding, conservation, or both. Breeding programs aim at improving honeybee production, behaviour, and resistance to pathogens, and managing genetic diversity that enables long-term response to selection. Conservation programs aim at preserving populations of endangered or native species by managing genetic diversity, reducing inbreeding depression, maintaining locally adaptive traits, and reducing the prevalence of pathogens.

The increased interest in honeybee breeding has spurred additional research in quantitative genetics of honeybees. Stochastic simulators are an essential tool for in-silico development and testing of quantitative genetic and statistical methods, and breeding strategies [13–16]. While simulations rely on many assumptions, they enable cost-effective and rapid testing of hypotheses before practical deployment. There are some simulators available for the most commercially relevant mammalian and plant species [13, 15, 16]. Due to the differences in biology and social organisation, these simulators cannot simulate honeybee populations. Although honeybee simulators have been developed, they are either too simplistic, do not simulate genomes and genetic and phenotypic values of individual honeybees, lack the flexibility to simulate the honeybee colony life cycle or the entire breeding program, or are not available as open source [14, 17]. One such honeybee simulator is BeeSim [14] that accounts for the quantitative genetics of the honeybees, but simulates quantitative values at the colony level, does not account for the colony events, and is also not publicly available. Another honeybee simulator, BEEHAVE [17], simulates colony and population dynamics and environmental variation to explore causes of colony failures and colony performance, but does not include genetics.

The aim of this work was to develop a holistic simulator of honeybee population management programs, SIMplyBee. SIMplyBee simulates (i) genomes as well as quantitative genetic and breeding values of individual honeybees and of whole colonies, (ii) major biological, reproductive, and organisational specificities of honeybees, and (iii) colony events. SIMplyBee is freely available from CRAN (http://cran.r-project.org/package=SIMplyBee) with extensive help pages, examples, and vignettes. See also http://www.SIMplyBee.info. We welcome contributions from the community at https://www.github.com/HighlanderLab/SIMplyBee. In the following, we describe the theory and technical implementation of SIMplyBee, demonstrate its use, and discuss its potential uses and plans for its future development.

## Implementation

SIMplyBee builds upon an established simulator, AlphaSimR [15, 18], and shares its core simulation principles and functionality. AlphaSimR is a stochastic simulator that simulates individuals with their corresponding genomes and quantitative genetic and phenotypic values. The most important classes in AlphaSimR are the SimParam class for global simulation parameters and the Pop class for objects that hold a group of individuals with their individual identification, parent identifications, as well as the genomes’ sequence and simulated traits’ values.

To enable a honeybee-specific simulation, SIMplyBee expands AlphaSimR with three classes: SimParamBee for global simulation parameters, Colony for a honeybee colony, and MultiColony for multiple honeybee colonies. Associated functions simulate honeybee populations and their events and facilitate an inspection or analysis of results. SIMplyBee’s functions address major honeybee specificities: honeybee genome, haplodiploid inheritance, complementary sex determination, social organisation, polyandry, and colony events. SIMplyBee includes five function groups related to: genome and genomic information, caste operations, colony and multicolony operations, quantitative genetics, and auxiliary operations. These functions operate at four levels with respect to the simplest object they return: level 0 being auxiliary functions returning standard R class objects such as vectors, matrices, and lists; level 1 returning an AlphaSimR Pop class object; level 2 returning a SIMplyBee Colony class object; and level 3 returning a SIMplyBee MultiColony class object. SIMplyBee includes over 16,000 lines of R code, documentation, and unit tests.

## Results

Here, we present the SIMplyBee functionalities by describing the underlying biological mechanisms behind the SIMplyBee functionality and by demonstrating its use. We describe: (i) how to simulate honeybee genomes; (ii) how to create a honeybee colony and its members; (iii) haplodiploid inheritance and complementary sex determination locus *CSD*; (iv) colony events; (v) how to work with multiple colonies; and (vi) honeybee genomics and quantitative genetics. Supplementary vignettes give further details for these and additional topics (Additional files 1, 2, 3, 4, 5, 6 and 7; http://SIMplyBee.info).

### Honeybee genome and initiating a honeybee simulation

**Fig. 1:**
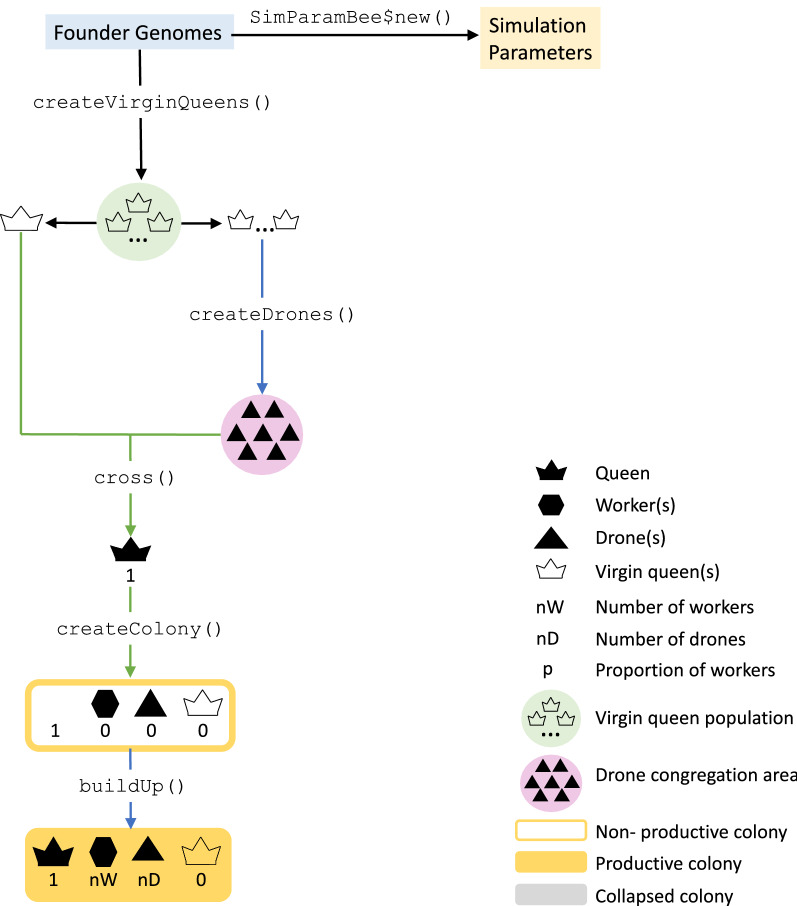
A flow chart of initialising a honeybee simulation in SIMplyBee. The first step is simulating a desired number of founder genomes and specifying the global simulation parameters in a new SimParamBee object. Next, we create the base virgin queens from the founder genomes. We can simulate any number (nInd) of virgin queens with the maximum being the number of simulated founder genomes. We choose one virgin queen as the future queen of the colony (left). On the other side (right), we select n virgin queens to provide drones for the DCA. We could select more virgin queens as future queens to create more colonies, and more virgin queen to contribute to the DCA. We next cross the virgin queen to a sample of drones from the DCA and use it to create a colony. We next build-up a colony, which adds in a desired number of workers and drones. The build-up also results in a productive colony

To initiate the simulation, we first need to simulate honeybee genomes and set simulation parameters (Fig. 1). The honeybee genome is small in its physical length, only 250 million bp, but large in its genetic length, 23 Morgans, due to a very high recombination rate of $2.3 \mathrm {\ x\ } 10^{-7}$ per bp [19]. SIMplyBee generates honeybee genome sequences with the approximate (Markovian) coalescent simulator MaCS [20]. It implements a state-of-the-art honeybee demographic model [21], allowing for the simulation of three subspecies: *A. m. ligustica*, *A. m. carnica*, and *A. m. mellifera*.

To start the simulation, the SIMplyBee package must first be installed and loaded:
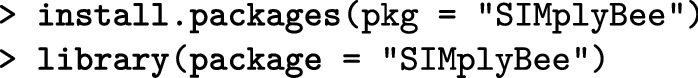


The first simulation step consists of generating founder honeybee genomes using simulateHoneybeeGenomes(). Here, we simulate 10 *A. m. carnica* honeybees with three chromosomes, each with 100 segregating sites. These numbers are not realistic, but enable a fast demonstration. Alternatively, we can import chromosome haplotypes, say from drones, or from phased queen or worker genotypes. Further details about initiating a simulation can be found in the Additional file 1.
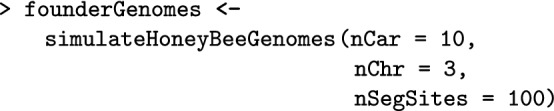


The second step is setting the global simulation parameters with SimParamBee, which builds upon the AlphaSimR class SimParam that contains global user-defined simulation parameters that apply to all individuals and populations, including genome and trait parameters, but also global pedigree and recombination events. In addition, SimParamBee holds honeybee-specific information: default numbers of workers (nWorkers), drones (nDrones), and virgin queens (nVirginQueens) in a full-size colony, the default number of drones that a queen mates with (nFathers), default proportions of workers that leave in a colony swarm (swarmP) or that are removed in a colony split (splitP), and the default percentage of workers that are removed during colony downsize (downsizeP). These default numbers can be changed according to the needs of a simulation or can be replaced by providing functions to sample numbers. For example, variable numbers of fathers, workers, drones, and virgin queens can be sampled from Poisson or truncated Poisson distributions (Additional file 7). Most SIMplyBee functions that take the number of individuals as an argument can accept these sampling functions as input, meaning that the output of such functions’ calls will be stochastic. SimParamBee also holds information about the *CSD* locus: the chromosome it is on (csdChr), its physical position on the chromosome (csdPos), and the number of alleles (nCsdAlleles). SimParamBee also holds the caste of each individual in the simulation that can be queen, father (drones that successfully mated and died), worker, drone, or virgin queen. The caste can change during the life of a honeybee. For example, after successful mating, a virgin queen becomes a queen and drones become fathers.

Here, we set the SimParamBee with the default number of workers in a colony being 100, the default number of drones in a colony being 10, and the *CSD* locus to have 32 alleles. We save the output to the SP object, which enables its direct use for other SIMplyBee functions without explicitly passing it as an argument.
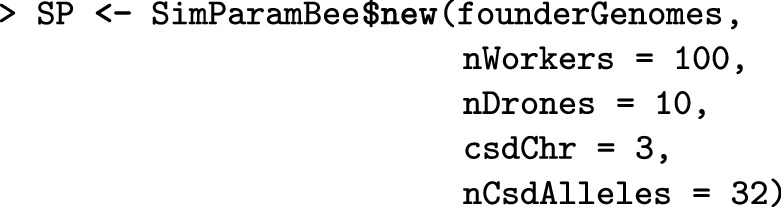


### Colony as an operational unit

A honeybee colony consists of individuals of two sexes, males, which are the drones, and females, which are differentiated into two castes, the queen and the workers. The queen is a single reproductive diploid female, the workers are non-reproductive diploid females that perform various colony maintenance tasks (collect food, nurse larvae, clean cells, etc.), and the drones are reproductive haploid males. A single colony can contain up to 65,000 workers [22] while drones can represent up to 20% of a honeybee colony [23]. In SIMplyBee, we accounted for this social organisation by creating a class Colony that holds all the above-mentioned individuals as AlphaSimR populations. For ease of use, all these groups are referred to as “castes”, including the drones that the queen mated with (fathers) and the virgin queens (queen-cells or emerged virgin queens). The Colony further contains technical information about the colony, its identification (id) and its location (location), coded as (latitude, longitude) coordinates, as well as logical information about past colony events: split, swarm, supersedure, or collapse. It also contains colony’s production status, which indicates whether production phenotypes can be collected from the colony. The latter is possible when the colony is built-up to its full size and has not swarmed. Production is turned off when a colony swarms, collapses, is downsized, or is split from another colony.

Here we show how to create a colony in SIMplyBee (Fig. 1). The createVirginQueens() function creates a base population of virgin queens (an AlphaSimR’s Pop class object) by recombining founder genomes. The isVirginQueen() function checks whether individuals are virgin queens. A similar is*() function checks the caste of each honeybee, where * is the inquired caste. These functions return TRUE or FALSE when an individual does or does not belong to the caste.
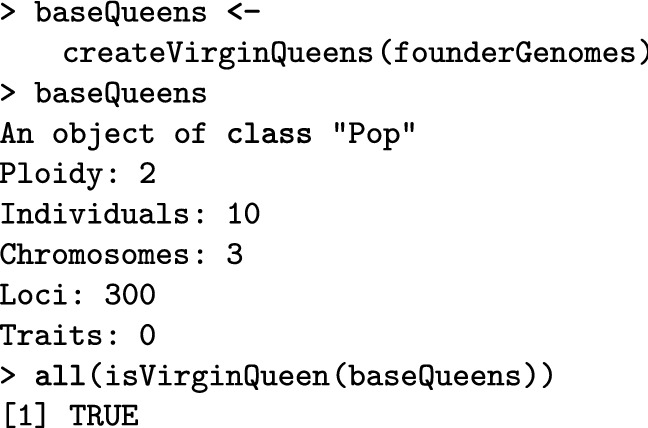


Here, the createColony() function creates a Colony object from the first virgin queen but can use n simulated queens to simulate n colonies. Printout of a Colony object returns its basic information: its id (1), location (not set, hence NA), queen (not yet available, hence NA), the number of fathers, workers, drones, and virgin queens, as well as the colony’s event statuses.
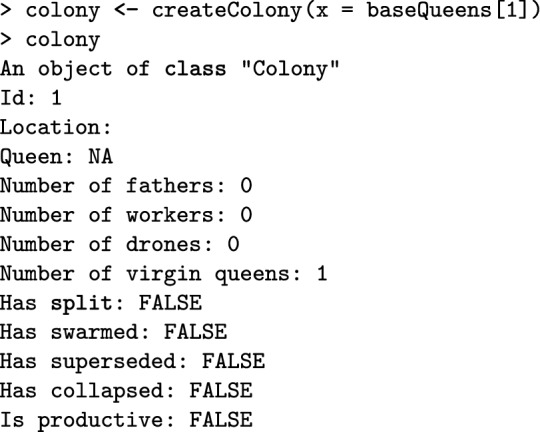


In honeybees, virgin queens mate with multiple drones, a phenomenon termed polyandry. A honeybee virgin queen will undergo several mating flights to a drone congregation area (DCA) that consists of thousands of drones from up to 240 colonies [24]. There, she will mate with 6 to 24 drones [25] and store the sperm in her spermatheca for life.

SIMplyBee contains a create*() function for each of the castes. For example, the createDrones() function creates drones from either a virgin queen, to kick-start the simulation in the absence of mated queens, or from a mated queen in a colony. This function can use more than one virgin queen to create a DCA. The simulation of genomes for these individuals is described in the next section (haplodiploid inheritance).
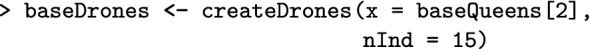


Here, the function cross() mates a virgin queen in the colony to the created drones. This promotes her to a queen so she can lay eggs for workers and drones. After the mating, the colony printout shows that the identification of the queen is “2”, that there are 15 fathers, and that there are no more virgin queens in the colony.
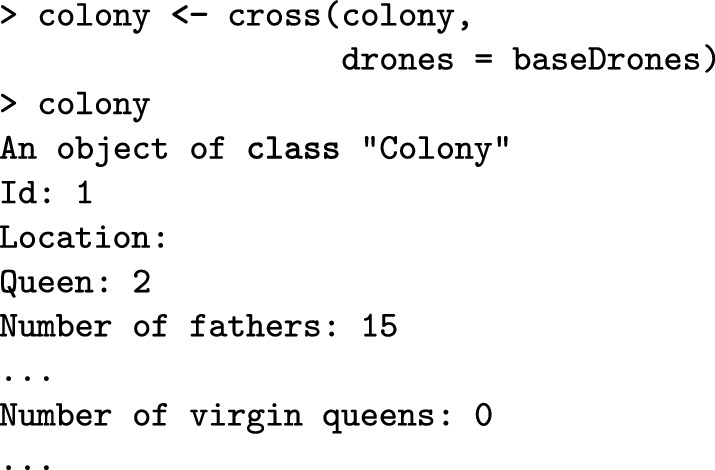


SIMplyBee includes additional functionality regarding open or controlled mating that are described in detail in the Crossing vignette in Additional file 4. There is a function to (i) create a DCA for open mating, createDCA(), or a DCA with drones from sister queens, as commonly found on honeybee mating stations, createMatingStationDCA(); (ii) sample a desired number of drones from a DCA, pullDroneGroupsFromDCA(); (iii) create a cross plan, which includes information about which drones will mate with each virgin queen, createRandomCrossPlan(); (iv) cross a virgin queen to a selected population of drones or according to a user-defined cross plan, cross().

Next, the buildUpColony() function builds-up the colony with a specified number of workers and drones. Without specifying these numbers, the function uses default numbers in the SimParamBee object. Building up the colony always switches the production status to TRUE.
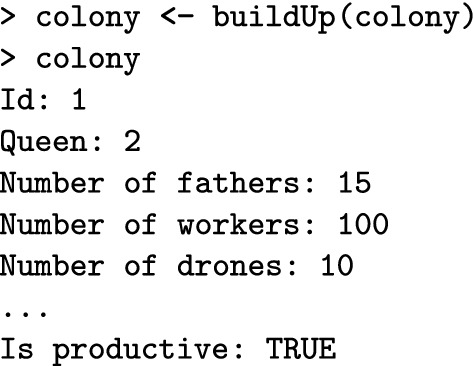


SIMplyBee also contains n*() functions to count individuals in each caste.
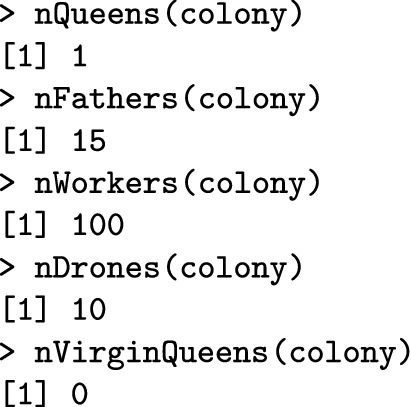


The get*() functions access individuals. Note that these functions copy individuals and hence leave individuals in the colony. Alternatively, the individuals can be “pulled” (and hence removed) from the colony with the pull*() functions.
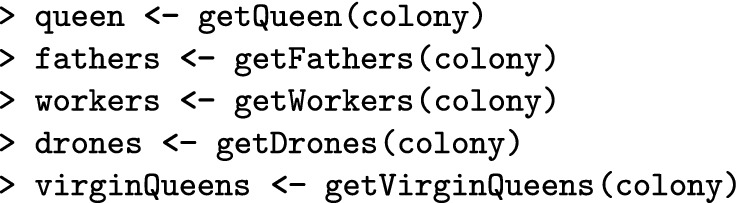


The getCaste() function accesses the caste information of every individual.
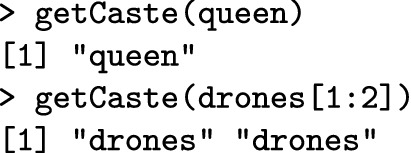


This function can be very useful when you have a group of honeybees and you do not know their source.
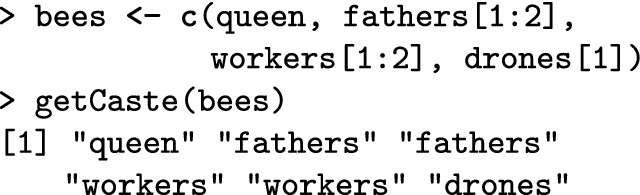


Additional functions for caste operations include obtaining the identifications of caste members and setting or getting the year of birth and age of the queen. The addCastePop(), replaceCastePop(), or removeCastePop() functions work with castes within a Colony object, and each return a modified Colony object.

### Haplodiploidy and *CSD*

Honeybees belong to the insect order Hymenoptera that is characterised by haplodiploid inheritance [26–28]. In SIMplyBee, we accounted for the haplodiploidy by simulating queens and workers as proper diploids and males as doubled haploids, which are fully homozygous individuals. However, we only use one (haploid) genome in all drone operations inside all SIMplyBee’s functions. Drones’ genomes are generated by recombining and segregating the queen’s genome. Workers’ and virgin queens’ genomes are generated by recombining and segregating the queen’s genome and segregating the fathers’ genomes. Hence, every simulated individual has a complete genome generated following the haplodiploid inheritance.

Besides haplodiploidy, sex in honeybees is determined by the complementary sex determination (*CSD*) locus. Fertilised eggs that are heterozygous at the *CSD* locus develop into diploid females, while homozygotes develop into diploid drones that are killed by workers [29]. In SIMplyBee, we assign a specific genomic region to represent the *CSD* gene. This region corresponds to the position of the *CSD* locus on chromosome 3 [30]. SIMplyBee simulates the *CSD* region as a sequence of non-recombining biallelic SNPs that determine a *CSD* allele. To account for balancing selection [31] at the *CSD* locus, we edit the initial founder genomes to achieve the desired number and frequency of *CSD* alleles in a population. The user can control the number of possible *CSD* alleles (2^length^) by controlling the length of the locus (in number of SNPs).

The getCsdAlleles() function retrieves *CSD* alleles and reports two non-recombining haplotypes for diploids as strings of 0s and 1s that represent respectively ancestral and mutation alleles along the *CSD* locus. The code below outputs the queen’s *CSD* alleles. The first row of the output shows locus identifications (chromosome_locus) and the first column shows haplotype identifications (individual_haplotype). The two sequences are different, meaning that the queen is heterozygous, as expected—otherwise her egg would have developed into a diploid drone that would have been killed by workers.
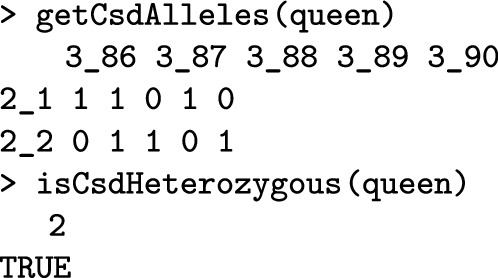


Heterozygosity of honeybees at the *CSD* locus is critical. The *CSD* alleles of this queen and the drones she mated with (compare getCsdAlleles(queen) and getCsdAlleles(fathers)—not shown) show no allele matches, which means we do not expect any homozygous brood in this colony. The pHomBrood() function calculates the theoretical brood homozygosity of a queen and the nHomBrood() function returns the realized number of homozygous offspring.
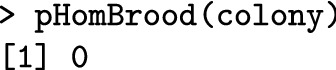


In the following, we create an inbred colony by mating a virgin queen from our colony with her brothers, and inspect the expected brood homozygosity.
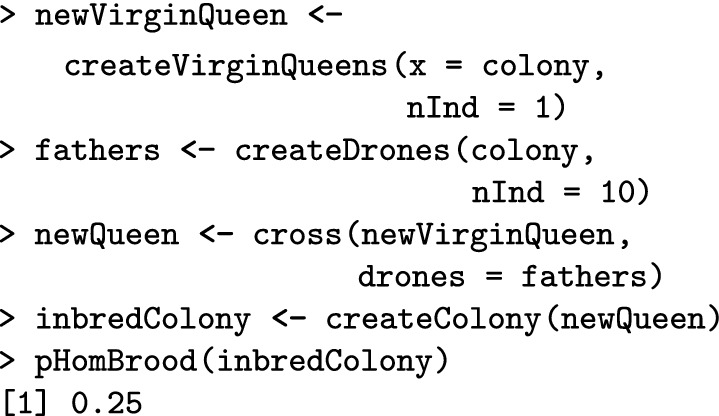


In this case, 25% of the diploid brood is expected to be homozygous. We now add workers to the colony to observe how many of them are homozygous. Inheritance is a random process, so the realised number of homozygotes will deviate from the expected proportion.
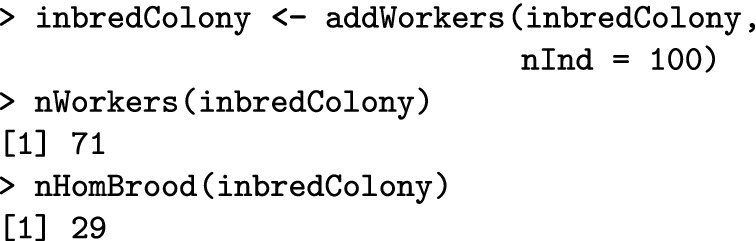


We aimed to add 100 workers, but we only got 71 due to CSD homozygous brood. The information about the number of homozygous brood is stored in the queen’s miscellaneous slot and is updated every time we create offspring from her.

### Colony events

A honeybee colony can experience a series of events during its life: swarming, superseeding, splitting, and collapsing. We present the details of colony events and their simulation in Additional file 3.

In swarming, a proportion of workers leave the hive with the queen, while the rest of the workers and the drones stay in the hive. New virgin queens emerge and compete in the colony. The winner undergoes mating flights as described above.

**Fig. 2:**
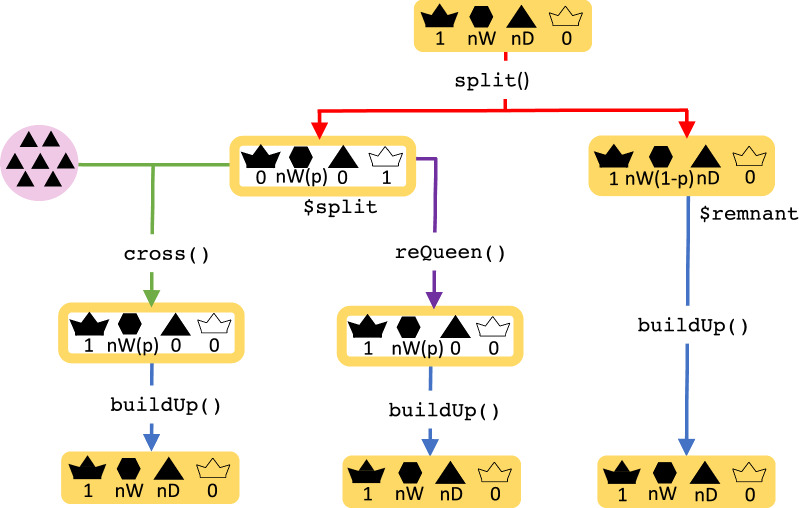
A flow chart of the colony swarming event in SIMplyBee. The swarm() function returns an R list with two colonies, swarm and remnant, both of which are non-productive. Parameter p represent the proportion of workers that leave with the swarm. After the swarm, the user can cross() the virgin queen of the remnant colony, or use an already mated queen from another source using reQueen(), which mimics the beekeepers’ options. Refer to the key in Fig. 1

The swarm() function swarms a colony (Fig. 2). The function takes the percentage of workers that leaves with the swarm, p. This can be either a fixed number or a function that samples p from either a uniform distribution or from a beta distribution that accounts for the number of individuals in a colony (colony strength). Further details about the sampling functions are described in Additional file 7. The swarm function returns an R list with two colonies: swarm, which contains the old queen and a proportion p of workers, and remnant, which contains the rest of workers, all the drones, and the virgin queens that are daughters of the queen that swarmed. The function also changes the swarm status to TRUE and the production status to FALSE
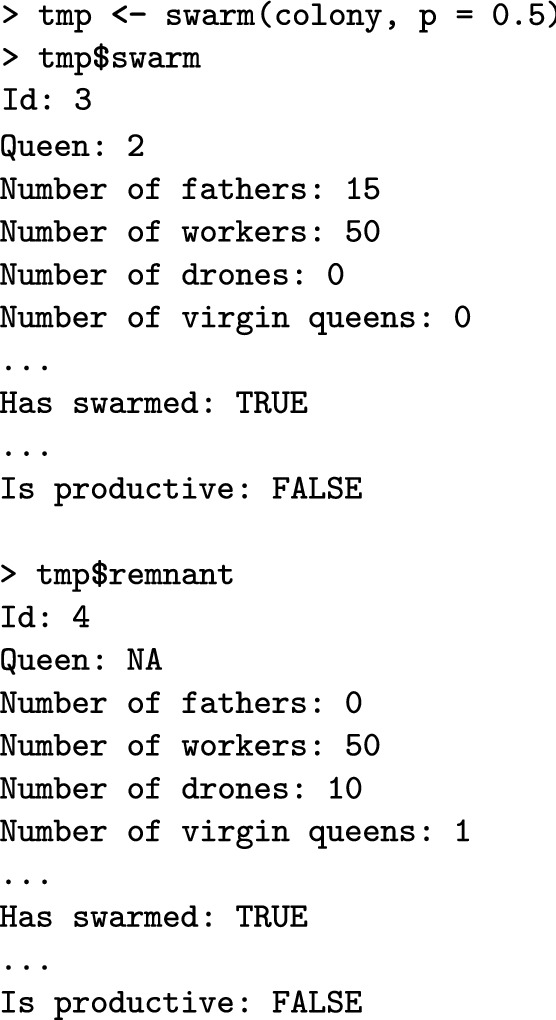


**Fig. 3:**
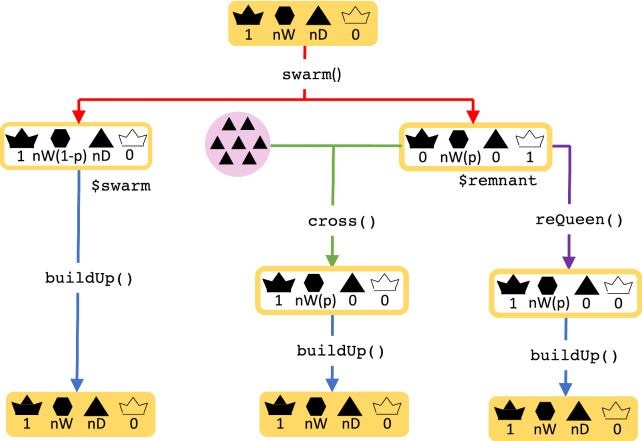
A flow chart of the colony splitting event in SIMplyBee. The split() function returns an R list with two colonies, split and remnant, where the *split* is non-productive and the remnant is productive. Parameter p represents the proportion of workers that are removed in a split. After the split, the user can cross() the virgin queen of the remnant colony, or use an already mated queen from another source using reQueen(), which mimics the beekeepers’ options. Refer to the key in Fig. 1

To build-up the population or prevent swarming, beekeepers often split strong colonies by taking away a proportion of the workers and starting a new colony with a new queen. The rest of the workers stay in the hive with the old queen. The split() function takes a colony and a proportion of the workers that are removed with the split, p. The split function returns an R list with two colonies: split, which contains the proportion p of workers taken from the main hive and virgin queens, and remnant, which contains the queen, the remaining workers, and drones (Fig. 3). After the split, the remnant colony is still productive, while the split is not.

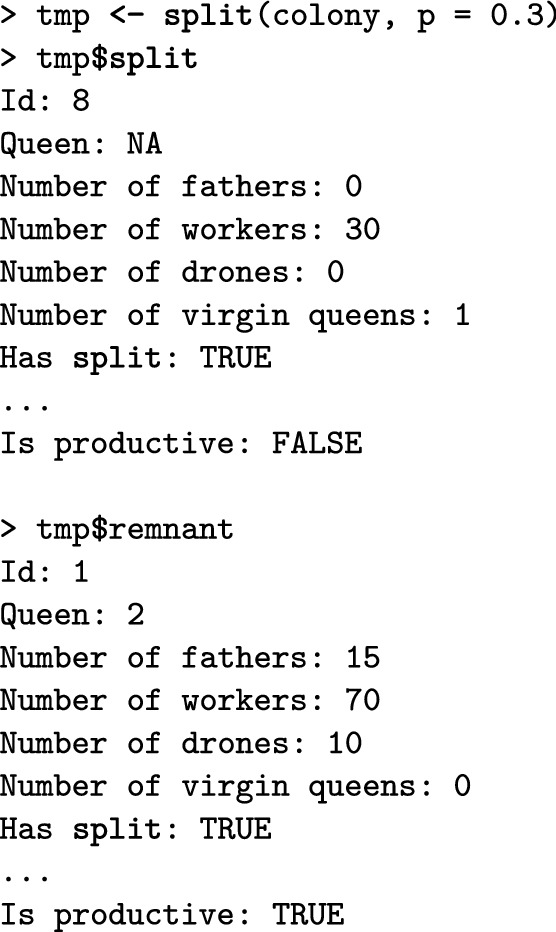



**Fig. 4:**
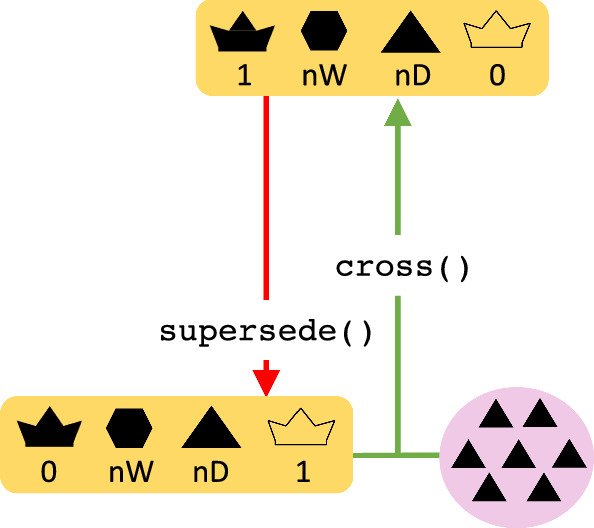
A flow chart of the colony supersedure event in SIMplyBee. The supersede() function returns a queen-less colony with a virgin queen. After a supersedure, a colony remains productive since the colony is still at its full size but a cross() is required for a new queen. Refer to the key in Fig. 1

In supersedure, the queen dies or is killed and its workers raise new virgin queens. The supersede() function removes the queen and produces new virgin queens from the brood (Fig. 4). After a supersedure, the colony is still productive because the workers are still present and working within the colony.

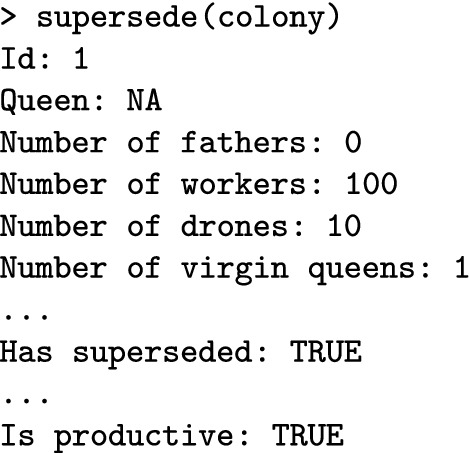



**Fig. 5:**
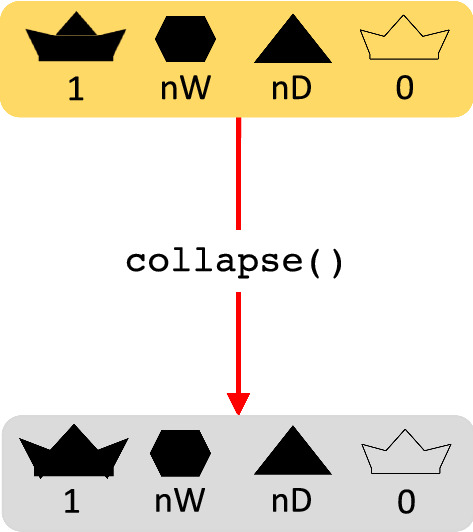
A flow chart of the colony collapse event in SIMplyBee. The collapse() function keeps all the individuals in a colony, but turns on the collapse parameter, hence marking the colony as collapsed and all the individual within it as dead. Further simulation with a collapsed colony is not allowed in SIMplyBee. Refer to the key in Fig. 1

Finally, some colonies can collapse due to the death of all its members. The collapse() function collapses a colony by changing the collapse status to TRUE (Fig. 5). The function keeps the individuals in the colony to enable the study of genetic and environmental causes that contributed to the collapse. In reality, dead honeybees would also be present in a collapsed colony.

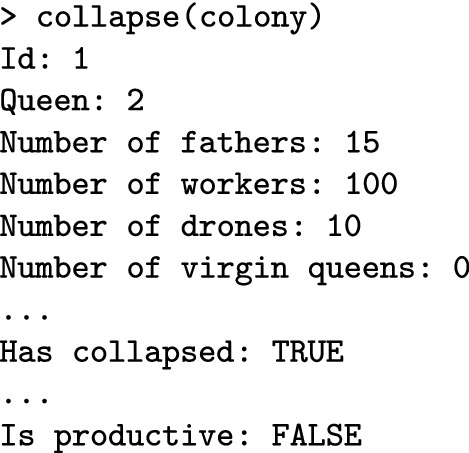



SIMplyBee also includes functions to build-up a colony, as shown above, a downsize() function that removes a proportion of the workers, all drones, and all virgin queens, and a function combine() that combines a strong and a weak colony. Additional details about simulating events are provided in Additional file 3.

### Working with multiple colonies

Beekeepers regularly work with a collection of colonies at the same time. The MultiColony class collects a list of colonies to represent an apiary, a region, an age group, etc. Additional details about working with multiple colonies are provided in Additional file 2.

The createMultiColony() function creates a MultiColony object. Here, we create an apiary with three virgin colonies. The printout of the object returns basic information, including the number of all, empty, and NULL colonies, and information about the colony events for the colonies in the Multicolony object.
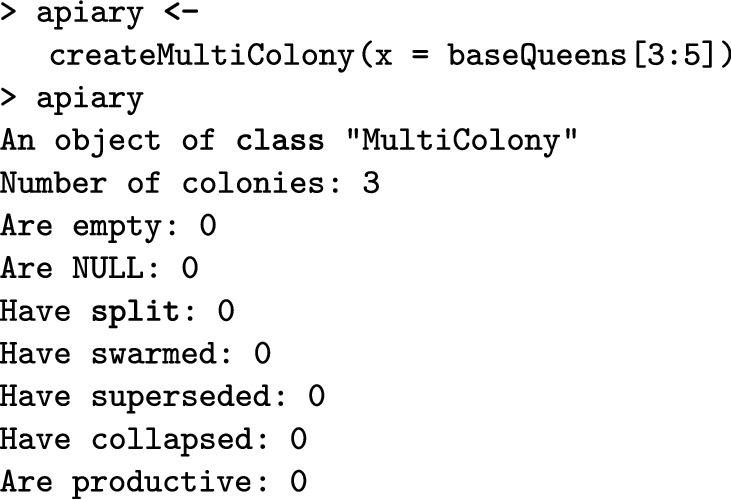


All functions for managing a Colony object can also be applied to a MultiColony object, which streamlines simulation scripts. These include functions for crossing, adding or removing individuals, simulating colony events, etc. Here, we demonstrate how to cross the apiary, build-up its colonies, and swarm some of them. The createDrones function creates a DCA from the base population virgin queens and the pullDroneGroupsFromDCA() function samples three groups of drones to mate the three virgin queens. As already mentioned, functions that sample individuals can use either fixed numbers or sampling functions (Additional file 7).
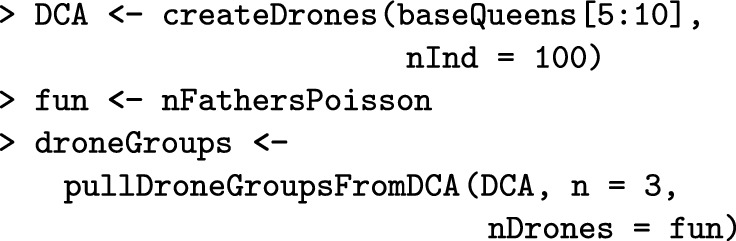


Next, the cross() function crosses the virgin queens in the apiary with the provided drone groups. To test for the presence of queens before and after mating to show that mating was successful, we can use is*Present() functions, where * is the caste, that check the presence of a caste in a colony. The buildUp() function builds-up all colonies in the MultiColony object.
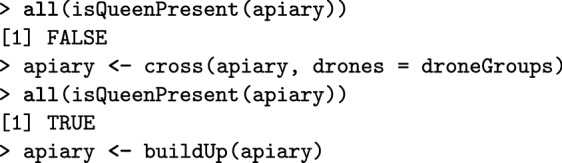


Next, the pullColonies() function samples one colony that will swarm. This returns an R list with two MultiColony objects: pulled with the sampled colonies, and remnant with the remaining ones. We save the latter back in the apiary.
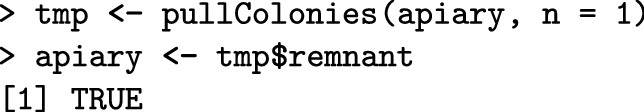


Now, the swarm function swarms the pulled colonies and returns an R list with two MultiColony objects, remnant and swarm. When applied to MultiColony, all the colonies are swarmed with the same p, unless specified otherwise. Additional details about simulating colony events for MultiColony are provided in Additional file 3.
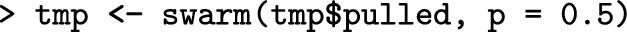


We can combine the colonies that did not swarm with the swarm remnant(s) into an updated apiary.
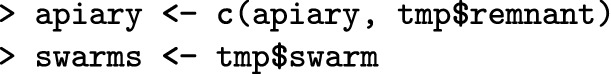


### Genomics and quantitative genetics

Similar to extracting the *CSD* genomic sequence, the functions get*Haplo() or get*Geno() extract whole-genome information for any set of individuals. Here, * can be SegSite to extract all segregating/polymorphic loci tracked in the simulation, Snp to extract marker loci, or Qtl to extract quantitative trait loci. There is also getIbdHaplo() to extract identity by descent information, with IBD alleles defined as those originating from the base population genomes. These functions leverage AlphaSimR functionality, but work with SIMplyBee Colony or MultiColony objects and in addition take the caste argument to extract information only for a specific caste. All this genome information is a result of haplodiploid inheritance. For example, the code below extracts genotypes of the first five workers in the colony at the first five tracked segregating sites. See further details in Additional file 5.
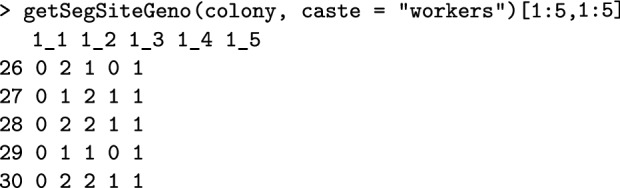


Honeybee phenotypes are characterized by two important phenomena. First, in honeybee keeping and breeding, phenotypes are mostly collected at the colony level as opposed to at the individual level. Second, phenotypes in honeybees are a complex interaction between queen and worker effects that are often negatively correlated [32, 33]. For most traits, the queen indirectly contributes to the colony phenotype by laying eggs [34, 35] and by stimulating the workers through pheromones [36, 37], while workers contribute directly by doing the actual work.

SIMplyBee simulates genetic and phenotypic values for each individual honeybee, but can also calculate colony-level values from individual-level values. Quantitative genetic simulation is initiated by specifying the assumptions about the genetic architecture of traits in SimParamBee, including the number of quantitative trait loci, the distribution of their effects, and genetic and environmental variances and covariances. In the following, we initiate another simulation and specify two negatively correlated traits that represent the queen and worker effect for honey yield. Additional details and a more extensive explanation of this simulation are provided in Additional file 6.
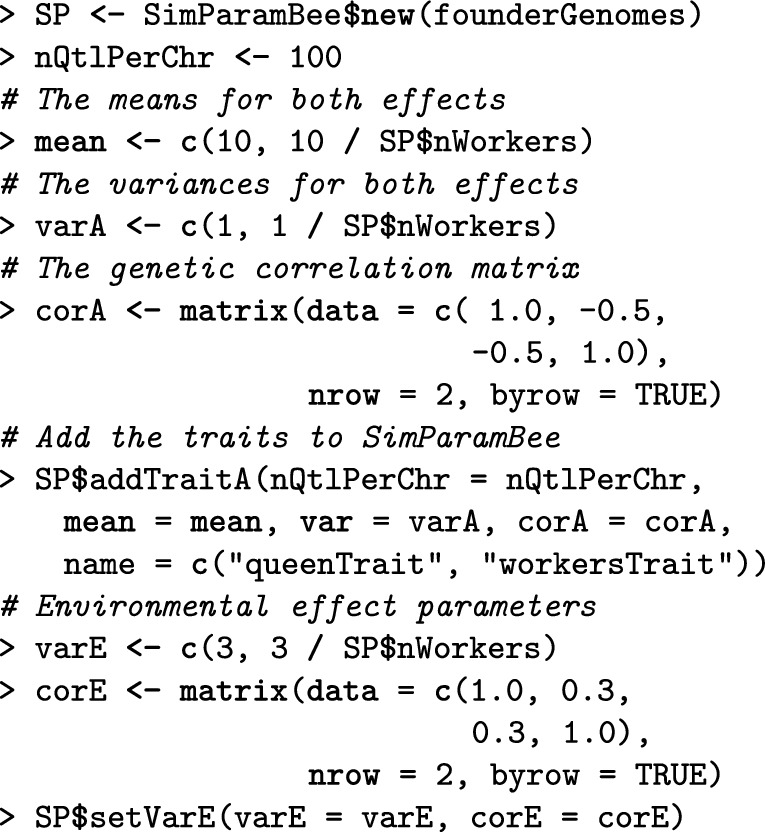


This initiation triggers calculation of individual-level genetic and phenotypic values. Using the AlphaSimR Pop class object, genetic and phenotypic values are stored in gv and pheno slots, respectively. They can be accessed with the getGv() and getPheno() functions, which both have the caste argument and work on Colony and MultiColony.
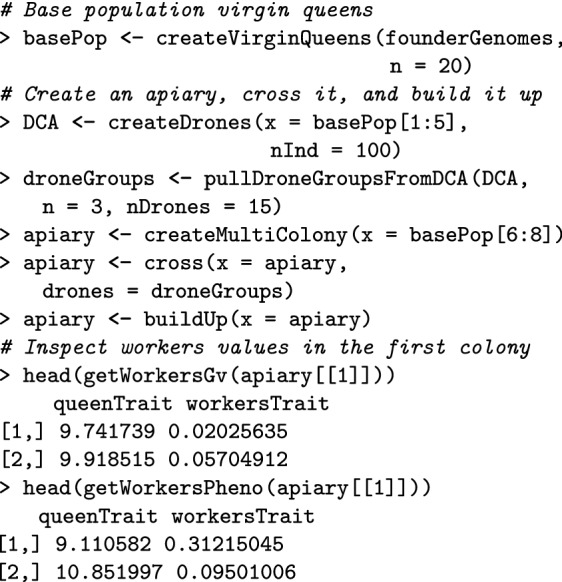


The calcColonyValue() function maps individual values to colony values using an established mapping function from the literature [14, 38, 39], but users can also provide their own mapping function. Examples of such quantitative genetic simulations of one or multiple correlated traits are shown in Additional file 6.

Here, we compute the colony-level genetic and phenotypic values for the colonies in our apiary.
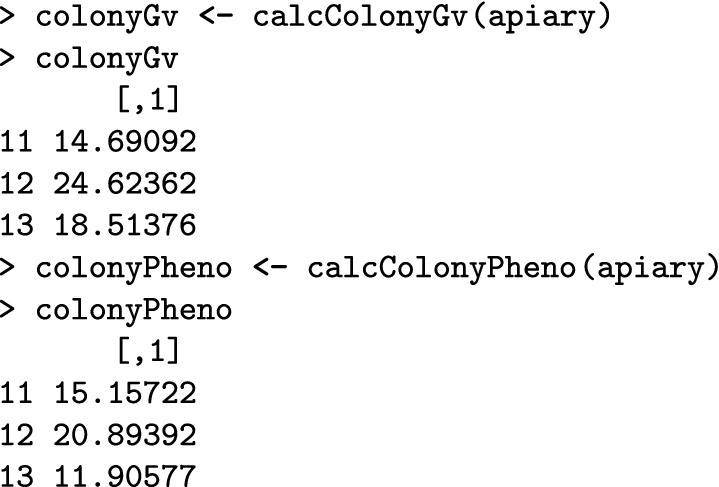


The best colony according to the genetic as well as the phenotypic value is the colony with ID “12”, hence we would select it for further reproduction. These values can be passed into the use parameter of the selectColonies() function.
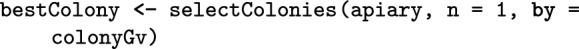


### Computing time

We tested the computing time needed to perform some basic actions with SIMplyBee: create drones, and create, cross, and build-up colonies (Additional file 8: Table S1). The results show that most operations take seconds but increase with larger numbers.

## Discussion

SIMplyBee is an R package for holistic simulation of honeybee breeding and conservation programs. In comparison to previously developed general genetics and breeding simulators [15, 16], it simulates honeybee-specific genomes, social organisation, and behaviours. SIMplyBee differs from previously developed honeybee-specific simulators [14, 17] by simulating individual honeybees, individual-level and colony-level quantitative values, and colony events that can affect genetic and phenotypic variation in a population.

### Potential uses of SIMplyBee

SIMplyBee provides a valuable research platform for testing different population-management decisions and answering various questions regarding the design of breeding schemes. SIMplyBee can be used to test the effect of various decisions in a breeding program on genetic gain, genetic diversity, and inbreeding; or to test the accuracy of inferences with competing quantitative genetic models. For example, users can test the effect of different phenotyping schemes by varying the frequency of phenotyping or the measuring scale. Furthermore, SIMplyBee can be used to test different mating control designs and the effect of varying the number of sires or drone-producing queens on a mating station. Users can also test different selection strategies by varying the time of selection, the number of selected queens, or the sources of information (pedigree, genomic, and phenotypic data). The list of potential studies is long. SIMplyBee is also a valuable platform for answering questions regarding the conservation of honeybees. Users might be interested in the effect of mating and management decisions on the genetic diversity in a population along the whole genome or only at the *CSD* locus; in comparing how different migration or import practices and associated policies affect genetic diversity; or how to design a conservation program to preserve genetic diversity.

### Why simulate individual bees?

Providing individual-level functionality might seem excessive since colony-level values in honeybees can be seen as equivalent to individual-level values in other species, say, mammals. However, there are at least seven reasons why this functionality supports current and future honeybee research. First, colonies are made up of individual honeybees and, thus, simulating individual honeybees with associated genomes and values is the correct thing to do. Second, having individual workers’ values allows different interactions between them to be simulated. For example, some phenotypes might be additive and the combined workers’ contribution is just a simple sum of individual values. This additive model has been shown for honey yield once the colony reaches a certain size to have a surplus of honey [22]. However, such a model cannot be assumed for all phenotypes. There is much debate and contradictory results regarding the contribution of individual bees to the colony-level phenotypic value. For example, for defensive behavior, some studies show that a single or a couple of aggressive workers in a colony can stimulate the rest and lead to highly defensive colony behavior [40], while others suggest an additive model [41]. Although this knowledge gap is understandable, given the considerable number of honeybees in a colony, future advances in sensor and beekeeping technologies and data science (machine learning) will provide ever more fine-grained data. Such data could further contribute to explaining the relationship between individual-level and colony-level phenotypes. To facilitate different models, SIMplyBee leaves the construction of the colony-level phenotype to the user, but it provides the additive model as the simplest example. As such, it can serve as a research platform for modeling different relationships and interactions. Third, simulating individuals within a colony enables the study of genetic relationships within and between colonies. Genetic variability can be driven by genetic processes and colony events. For example, splitting and swarming can substantially affect genetic variability within a colony. Fourth, related to the latter, individual-level simulation allows studying and developing methods to compute genetic relationships in honeybees for systems that deviate from the commonly studied breeding design [38, 39]. Fifth, having individual genomes allows studying how pooled genotyping samples, commonly used for honeybees, represent the queen’s or the colony’s genetics [42], either in parentage testing and discovery, or for quantitative genetic analyses, such as genomic prediction and genome-wide association studies. Sixth, simulating individual drones inherently simulates multiple patrilines within a colony, a patriline being all offspring of a single drone. This simulates a genetically diverse colony and allows the potential differential contributions of partilines to colony performance to be modelled [43]. And seventh, researchers have already begun to collect individual-level drone phenotypes, for example, information on drone sperm quality [44].

### Genetic values and its statistical components

SIMplyBee can serve as a research platform to test assumptions about current quantitative genetic models [14, 38, 39]. Quantitative genetic models can estimate the joint effect of workers and the effect of the queen on the colony-level phenotype. However, sometimes simpler models are used [33, 45]. Based on the worker and queen effect estimates, the performance, selection, and inheritance criteria can be created [46].

Another challenge that we faced in developing SIMplyBee was in providing functionality to calculate statistical genetic values, that is, breeding values, dominance deviations, and epistasis deviations. Since SIMplyBee leverages AlphaSimR [15], these values can be calculated using AlphaSimR’s bv(), dd(), and aa() functions. However, caution is required since traditional formulae for these statistical components of genetic values assume Hardy-Weinberg equilibrium [47] and are computed “relative” to the population of individuals at hand. The latter means that for a honeybee simulation we would either have to report these values relative to each colony population, which would make the output relevant only for each colony, or we would have to create a large “meta” population object of all currently living honeybees. Further development is required to address this aspect in SIMplyBee.

### Future development

Future development of the SIMplyBee package will focus on additional features and on improving the functionality and efficiency of existing features. Our immediate focus is on the following three features. First, on developing a new honeybee demographic model to include more subspecies and to improve estimates of model parameters [48]. While SIMplyBee currently uses MaCS [20] to simulate the genome, we will consider novel simulators that may offer more flexibility and computational speed, such as msprime (backward in time) [49, 50] and SLiM (forward in time) [51] simulators. These simulators rely on a public library of species’ genome information and demographic models, named stdpopsim, to which we have already added the honeybee [52, 53]. Second, we will further optimize the speed and memory performance of SIMplyBee. A simulation of a real-size honeybee colony or a breeding program with such colonies can be computationally demanding because a single colony can hold up to several tens of thousands of workers. By timing some of the basic SIMplyBee functions, we identified that the current mating implementation is slow (Additional file 8: Table S1). However, mating a large number of queens by proving a single drone population and a mating plan (crossPlan) halves computing times compared to mating queens to predefined drone populations (“drone packages”). Users can also decrease the computational burden by simulating workers and drones only when needed, for example, drones at the time of mating and workers at the time of generating and analyzing phenotypes. We plan to further optimize SIMplyBee by allowing workers’ contributions to colony-level phenotypes to be simulated without storing the workers, which saves some time, but mostly memory. Such a solution has already been implemented in AlphaSimR for the simulation of hybrid plant breeding programs. Running time can also be decreased by, for example, working with the expectation and variance of genetic values in progeny [54, 55] instead of simulating tens of thousands of workers. We will also strive to optimize functions by leveraging C++ via the Rcpp package [56]. Third, we will add a geospatial component to the simulation. Colony location plays a major role in honeybee mating and colony performance. The current implementation enables setting the location of every Colony and MultiColony object. We will develop functionality to create a DCA or sample the drones for a virgin queen mating according to the location of colonies, for example, in a certain radius, since virgin queens are more likely to mate with drones from nearby colonies. We will also add spatially-aware simulation of environmental effects. Honeybee colony performance depends heavily on the environment in terms of food provision, weather, pests, etc. Such environmental conditions usually change continuously through space, hence colonies that are closer together usually experience more similar environmental conditions than colonies that are further apart. The framework for such spatially aware simulation and modelling has already been developed and tested in a livestock setting [57].

We invite the honeybee genetics and breeding community to join us in the future development of SIMplyBee. The development is hosted on GitHub at https://github.com/HighlanderLab/SIMplyBee. We welcome users and developers to fork this git repository and provide “pull request (PR)” contributions. Each pull request is reviewed by one of the developers within the core team. Based on the review, pull requests will be updated before being merged into the development branch. The development branch is periodically merged into the main branch for publication on CRAN and for user installation. For each function we request documentation with examples and unit tests to ensure future changes will not break the functionality.

This work describes the usage of SIMplyBee for simulating honeybee populations. However, other bee species share a similar organisation and behaviour as the honeybee. Hence, SIMplyBee could also be used to simulate other *Apis* species. For example, *Apis florea*, the dwarf honeybee, and *Apis cerana*. *Apis florea* importantly contributes to pollination in some countries of the Middle East and Asia. Its range is predicted to increase due to climate change [58] and SIMplyBee could be used to model a breeding program for this bee species as well.

## Conclusions

This paper presents a stochastic simulator, SIMplyBee, for holistic simulation of honeybee populations and population management programs. SIMplyBee builds upon its predecessors by simulating genomes of individual honeybees and corresponding individual-level genetic and breeding values. SIMplyBee stores individual honeybees as the caste populations within a colony object, which enables the simulation of colony events and calculation of colony-level quantitative values. Colonies can be further organised into multi-colony objects for ease of use. SIMplyBee provides a valuable research platform for honeybee genetics, breeding, and conservation. Possible uses include testing the effects of breeding or conservation decisions on genetic gain and genetic variability in honeybee populations, testing the performance of existing and novel statistical methods, etc. Future directions include improvements to the simulation of honeybee chromosomes through new demographic models, the addition of spatial awareness in mating and phenotype simulation, reducing computational bottlenecks, and encouraging community engagement. We invite the honeybee genetics and breeding community to collaborate with us in improving SIMplyBee.

## Supplementary Information

**Additional file 1**. Honey biology vignette. This vignette introduces SIMplyBee package by describing anddemonstrating how SIMplyBee implements honeybee biology. Specifically, itdescribes how to initiate simulation with founder genomes and simulationparameters, how to create and build-up a colony, the colony structure, andcomplementary sex determininglocus [20, 21] This vignette can also be found on https://cran.r-project.org/package=SIMplyBee and http://www.SIMplyBee.info.

**Additional file 2**. Multiple colonies vignette. This vignette introduces working with multiple colonies bydemonstrating how to create and work with MultiColony objects inSIMplyBee. This vignette can also be found on https://cran.r-project.org/package=SIMplyBee and http://www.SIMplyBee.info.

**Additional file 3**. Colony events vignette. This vignette introduces the colony events and how tosimulate them in SIMplyBee. It shows how to simulate swarming, splitting,superseding, and collapsing either a Colony or MultiColony objects[59–61]. This vignette can also be found on https://cran.r-project.org/package=SIMplyBee and http://www.SIMplyBee.info.

**Additional file 4**. Crossing vignette. This vignette demonstrated how to cross virgin queens inSIMplyBee. It demonstrates how to cross a single or multiple virgin queens,cross either with pre-selected population/group of drones or according to across plan, and cross queens on an open DCA or mating station. This vignette can also be found on https://cran.r-project.org/package=SIMplyBee and http://www.SIMplyBee.info.

**Additional file 5**. Genomics vignette. This vignette demonstrates how to obtain genomic informationof simulated honeybees. It also demonstrates, how to compute honeybeegenomic relationship matrices in SIMplyBee [62–67]. This vignette can also be found on https://cran.r-project.org/package=SIMplyBee and http://www.SIMplyBee.info.

**Additional file 6**. Quantitative genetics vignette. This vignette describes and demonstrates how SIMplyBeeimplements quantitative genetics principles for honeybees. Specifically, itdescribes three different examples where we simulate a single colony trait,two colony traits, and two colony traits where one trait impacts the otherone via the number of workers. This vignette can also be found on https://cran.r-project.org/package=SIMplyBee and http://www.SIMplyBee.info.

**Additional file 7**. Sampling functions vignette. This vignette introduces sampling functions that sample eitherthe number of caste individuals or the proportion of workers that stay orare removed in colony events. This vignette can also be found on https://cran.r-project.org/package=SIMplyBee and http://www.SIMplyBee.info.

**Additional file 8**. Computing time. The table shows the mean computing time for basicSIMplyBee functions of ten replicates. It shows the time to create ten or amillion drones; create ten or a thousand empty or virgin colonies; to crossten or a thousand colonies by providing n drone populations, where n is the number of virgin queens, or by providing a singledrone population and a cross plan; and to build-up ten or a thousandcolonies to a thousand or 60 thousand workers.

## Data Availability

The data and material for this study are available at SIMplyBee website http://www.SIMplyBee.info, CRAN https://cran.r-project.org/package=SIMplyBee, and the SIMplyBee GitHub repository https://github.com/HighlanderLab/SIMplyBe.
