# Supplementary material for: SIMplyBee: an R package to simulate honeybee populations and breeding programs
Source: Genet Sel Evol. 2023 May 9;55:31. doi: 10.1186/s12711-023-00798-y (PMC10169377; doi:10.1186/s12711-023-00798-y)
Supplement: Supplementary file 1 — Additional file 1. Honey biology vignette. This vignette introduces SIMplyBee package by describing anddemonstrating how SIMplyBee implements honeybee biology. Specifically, itdescribes how to initiate simulation with founder genomes and simulationparameters, how to create and build-up a colony, the colony structure, andcomplementary sex determininglocus [20, 21] This vignette can also be found on https://cran.r-project.org/package=SIMplyBee and http://www.SIMplyBee.info. [file 12711_2023_798_MOESM1_ESM.pdf]

Additional file 1 - Honeybee biology vignette

2023-03-24

Introduction

This vignette describes and demonstrates how SIMplyBee implements honeybee biology. Specifically, it describes:

1. initiating simulation with founder genomes and simulation parameters,
2. creating and building up a colony,
3. colony structure, and
4. complementary sex determining (*CSD*) locus.

First, you need to install the package with `install.packages(pkg = "SIMplyBee")`.

Now load the package and dive in! You load the package by running:

```
library(package = "SIMplyBee")
#> Loading required package: AlphaSimR
#> Loading required package: R6
#>
#> Attaching package: 'SIMplyBee'
#> The following object is masked from 'package:base':
#>
#>     split
```

Initiating simulation with founder genomes and global parameters

Figure 1 visualizes the initiation of the simulation. First, we simulate some honeybee genomes that represent the founder population. You can quickly generate random genomes using AlphaSimR's `quickHaplo()`. These founder genomes are rapidly simulated by sampling 0s and 1s, and do not include any species-specific demographic history. This is equivalent to all loci having allele frequency 0.5 and being in linkage equilibrium. We use this approach only for demonstrations and testing.

Alternatively, you can more accurately simulate honeybee genomes with SIMplyBee's `simulateHoneybeeGenomes()`. This function simulates the honeybee genome using MaCS [20] for three subspecies: *A. m. ligustica*, *A. m. carnica*, and *A. m. mellifera* according to the demographic model described by [21].

As a first demonstration, we will use `quickHaplo()` and simulate genomes of two founding individuals. In this example, the genomes will be represented by only three chromosomes and 1,000 segregating sites per chromosome. Honeybees have 16 chromosomes and far more segregating sites per chromosome, but we want a quick simulation here.

```
founderGenomes <- quickHaplo(nInd = 2, nChr = 3, segSites = 100)
```

Alternatively, we use `simulateHoneybeeGenomes()` to sample genomes of a founder population including 4 *A. m. mellifera* (North) individuals and 2 *A. m. carnica* individuals. The genomes will be represented by only three chromosomes and 5 segregating sites per chromosome. These numbers are of course extremely

low because we want a quick examample for demonstrative reasons. This chunk of code should take a few minutes to run.

```
founderGenomes2 <- simulateHoneyBeeGenomes(nMelN = 4,
                                            nCar = 2,
                                            nChr = 3,
                                            nSegSites = 5)
```

Unfortunately, due to the complexity of this function, even using such small numbers takes a while to run. Simulating a group of founder genomes with more realistic numbers will therefore require a lot of time to run. We suggest running this part to an external server and save the outcome as an RData file, which we can load in our environment and work with it.

```
save(founderGenomes2, file="FounderGenomes2_3chr.RData")
```

Besides specifying the number of individuals, chromosomes, and segregating sites, `simulateHoneybeeGenomes()`, also takes a number of genomic parameters: effective population size, ploidy, length of chromosomes in base pairs, genetic length of a chromosome in Morgans, mutation rate, recombination rate, and time of population splits. The default values for these numbers follow published references [21]. While you can change these parameters, we don't advise doing this because such demographic models, and their parameters, are estimated jointly, so we should not be changing them independently. You can read more about these parameters in the help page:

```
??simulateHoneybeeGenomes
```

Now we are ready to setup global simulation parameters using `SimParamBee`. `SimParamBee` builds upon AlphaSimR's `SimParam`, which includes genome and trait parameters, but also global pedigree and recombination events. We usually save the output of `SimParamBee` as the `SP` object (we will assume this in all vignettes). Namely, all SIMplyBee functions will use this object if you don't directly specify `simParamBee` argument. `SimParamBee` additionally holds honeybee specific simulation information (Figure 1):

- default number of workers (`SP$nWorkers`) and drones (`SP$nDrones`) in a full-sized colony; these numbers are used by functions such as `createWorkers/Drones()`, `addWorkers/Drones()` and `buildUp()`;
- default number of drones that a virgin queen mates with (`SP$nFathers`)
- the *CSD* information: the chromosome of the *CSD* (`SP$csdChr`), the position (`SP$csdPos`), and the desired number of *CSD* alleles in a population (`SP$nCsdAlleles`). The number of *CSD* alleles determines the length of the *CSD* locus (`SP$nCsdSites`): `nCsdAlleles = nCsdSites**2`. By default, the *CSD* is placed on its real genomic position on chromosome 3. However, if the user simulates less than three chromosomes, the *CSD* is placed on chromosome 1;
- pedigree for each individual created in the simulation (`SP$pedigree`) if requested by `SP$setTrackPed(TRUE)`; and
- caste information for each individual created in the simulation (`SP$caste`).

You can read more about the `SimParam` and `SimParamBee` in their help pages (`help(SimParam)` and `help(SimParamBee)`).

Below we use set the number of *CSD* alleles and default number of workers and drones in a colony:

```
SP <- SimParamBee$new(founderGenomes, nCsdAlleles = 32)
SP$nWorkers <- 100
SP$nDrones <- 10
```

After creating the `SimParamBee` object, you can inspect it! This returns a lot of output and we suggest you return back to this point once you are comfortable with the basic functionality!

```
print(SP)
```

From the simulated founder genomes, we can create virgin queens (Figure 1). These will serve as our our first honeybee individuals (the so called base or founder population). In AlphaSimR and SIMplyBee,

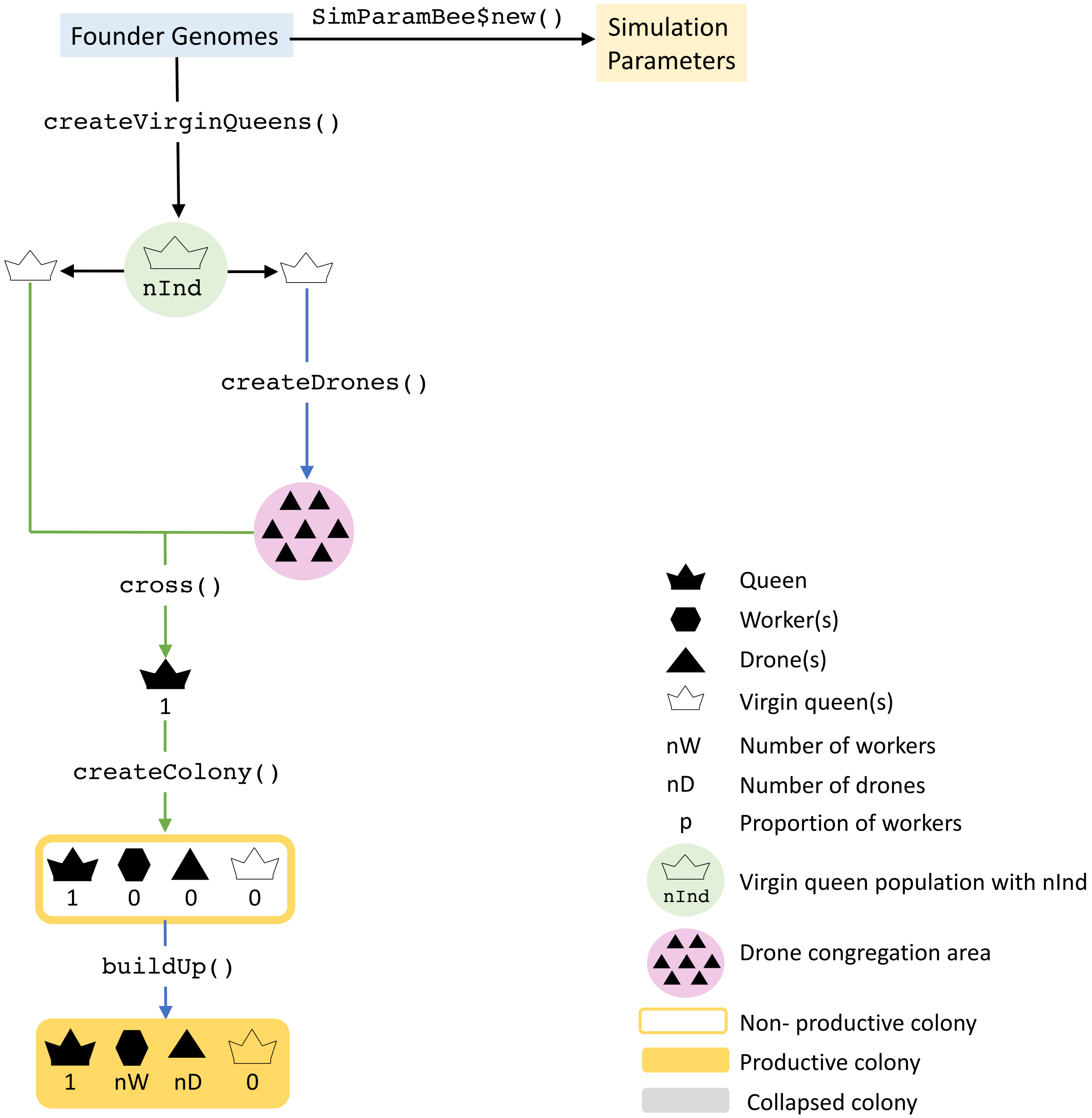

Figure 1: Simulation initiation.

individuals are stored in Pop class objects, that hold a group of individuals with their individual identification, parent identifications, as well as genomes and trait values. So, the basePop is a population (Pop class object) of two individuals, our two virgin queens. If we print out basePop, we see some basic information about the population: the ploidy, number of individuals, chromosome, loci, and traits. We next check whether our individuals are of certain caste with is*() functions, where * can be either queen, worker, drone, virginQueen, or father. These functions return TRUE if the individual is a member of the caste in question and FALSE is it is not. These functions check the caste information in the SP$caste. Here, we use isVirginQueen() to check whether our base population individuals are virgin queens.

```
baseQueens <- createVirginQueens(founderGenomes)
baseQueens
#> An object of class "Pop"
#> Ploidy: 2
#> Individuals: 2
#> Chromosomes: 3
#> Loci: 300
#> Traits: 0
isVirginQueen(baseQueens)
#> [1] TRUE TRUE
```

Similarly, you can use the function getCaste() to get the caste of each individual.

We will use the first virgin queen to create five drones for future mating. Note that virgin queens do not create drones. Only queens with colonies create drones. However, to get the simulation up and running, we need drones and the function createDrones() can work both with virgin queens or colonies (we will present colonies in the next section). You can use more than one virgin queen to create the drones or even an entire drone congregation area (DCA) with as many drones per virgin queen as you want (nInd).

```
baseDrones <- createDrones(x = baseQueens[1], nInd = 15)
baseDrones
#> An object of class "Pop"
#> Ploidy: 2
#> Individuals: 15
#> Chromosomes: 3
#> Loci: 300
#> Traits: 0
```

Creating and building up a colony

We will use the other virgin queen to create a colony. You can use more than one virgin queen to create more than one colony. In SIMplyBee, a honeybee colony is stored in an object of Colony class. You can create a new colony with the function createColony(). You can create a completely empty colony or a colony with either a virgin or a mated queen. The Colony class organises all its members in five castes: queen, fathers, workers, drones, and virginQueens. We describe the castes in next section. The Colony further contains technical information about the colony, its identification id and location coordinates coded as (latitude, longitude). Further, it contains logical information about the past colony events: split, swarm, supersedure, or collapse. It also contains production status, which indicates whether we can collect a production phenotype from the colony. The latter is possible when the colony is built-up to its full size and has not swarmed. The production is turned off when a colony downsizes, collapses, or swarms, and for the split of a split colony. You will learn about these colony events in the Colony events vignette.

```
colony <- createColony(x = baseQueens[2])
colony
#> An object of class "Colony"
#> Id: 1
#> Location:
```

```
#> Queen: NA
#> Number of fathers: 0
#> Number of workers: 0
#> Number of drones: 0
#> Number of virgin queens: 1
#> Has split: FALSE
#> Has swarmed: FALSE
#> Has superseded: FALSE
#> Has collapsed: FALSE
#> Is productive: FALSE
```

We see all the above mentioned information in the printout of the `Colony` object. For this specific colony, we see that the ID of the colony is "8", the location is not set, and there is no queen (hence `NA`). There are consequently no fathers in the colony, nor any workers, drones or virgin queens. All the events are set to `FALSE` (you will learn more about events in the Colony events vignette) and the colony is not productive, since it does not include any individuals.

Let's now mate our virgin queen, so that she is promoted to a queen and can start laying eggs of her own workers and drones.

```
colony <- cross(colony, drones = baseDrones)
colony
#> An object of class "Colony"
#> Id: 1
#> Location:
#> Queen: 2
#> Number of fathers: 15
#> Number of workers: 0
#> Number of drones: 0
#> Number of virgin queens: 0
#> Has split: FALSE
#> Has swarmed: FALSE
#> Has superseded: FALSE
#> Has collapsed: FALSE
#> Is productive: FALSE
```

We see that the virgin queen is now a queen - hence we have a queen with the ID "2" and no virgin queens in our colony.

Next, let's build up our colony using the function `buildUp()` that adds in workers and drones. This function takes parameters `nWorkers` and `nDrones`, where we specify how many workers and drones to add. However, if these numbers are not specified in the function's call, the function uses the default numbers from the `SimParamBee` object (`SP$nWorkers` and `SP$nDrones`). This function also always turns the `production` status to `TRUE`, since it assumes we are building the colony up to its full-size.

```
buildUp(colony, nWorkers = 10, nDrones = 7)
#> An object of class "Colony"
#> Id: 1
#> Location:
#> Queen: 2
#> Number of fathers: 15
#> Number of workers: 10
#> Number of drones: 7
#> Number of virgin queens: 0
#> Has split: FALSE
#> Has swarmed: FALSE
```

```
#> Has superseded: FALSE
#> Has collapsed: FALSE
#> Is productive: TRUE
buildUp(colony)
#> An object of class "Colony"
#> Id: 1
#> Location:
#> Queen: 2
#> Number of fathers: 15
#> Number of workers: 100
#> Number of drones: 10
#> Number of virgin queens: 0
#> Has split: FALSE
#> Has swarmed: FALSE
#> Has superseded: FALSE
#> Has collapsed: FALSE
#> Is productive: TRUE
```

All the functions in SIMplyBee return objects, hence we need to save them as an object, otherwise they are lost.

```
colony <- buildUp(colony)
colony
#> An object of class "Colony"
#> Id: 1
#> Location:
#> Queen: 2
#> Number of fathers: 15
#> Number of workers: 100
#> Number of drones: 10
#> Number of virgin queens: 0
#> Has split: FALSE
#> Has swarmed: FALSE
#> Has superseded: FALSE
#> Has collapsed: FALSE
#> Is productive: TRUE
```

Colony structure

Lets explore our colony. In every colony we have different groups of individuals (castes). These include: queen, fathers, workers, drones, and virgin queens. The queen controls the colony, workers do all the hard work, drones disseminate queen's genes, and one of the virgin queens will eventually replace the queen. We also store fathers, which represent drones that the queen mated with. The fathers caste is effectively the drone sperm stored in queen's spermatheca. Storing fathers enables us to generate colony members on demand. SIMplyBee contains `n*()` functions to count the number of individuals in each caste, where `*` is `queen`, `fathers`, `workers`, `drones`, and `virginQueens`. Let's count how many individuals we have for each caste in our colony.

```
nQueens(colony)
#> [1] 1
```

```
nFathers(colony)
#> [1] 15
```

```r
nWorkers(colony)
#> [1] 100
```

```r
nDrones(colony)
#> [1] 10
```

```r
nVirginQueens(colony)
#> [1] 0
```

Next, we can access the individuals of each caste with `get*()` functions. These functions leave the colony and its members intact (they do not change the colony) by copying the individuals.

```r
(queen <- getQueen(colony))
#> An object of class "Pop"
#> Ploidy: 2
#> Individuals: 1
#> Chromosomes: 3
#> Loci: 300
#> Traits: 0
```

```r
(fathers <- getFathers(colony))
#> An object of class "Pop"
#> Ploidy: 2
#> Individuals: 15
#> Chromosomes: 3
#> Loci: 300
#> Traits: 0
```

```r
(workers <- getWorkers(colony))
#> An object of class "Pop"
#> Ploidy: 2
#> Individuals: 100
#> Chromosomes: 3
#> Loci: 300
#> Traits: 0
```

```r
(drones <- getDrones(colony))
#> An object of class "Pop"
#> Ploidy: 2
#> Individuals: 10
#> Chromosomes: 3
#> Loci: 300
#> Traits: 0
```

```r
(virginQueens <- getVirginQueens(colony))
#> NULL
```

As you see above, there are no virgin queens present in the colony at this moment, since the queen is active. Future colony events might change this.

Should you want to pull out, that is, remove castes or their members, have a look at `pull*()` functions. These functions return a list of objects: `pulled` being the pulled individuals (`Pop` object), and `remnant` being the remaining colony without the pulled individuals.

```r
tmp <- pullWorkers(colony, n = 10)
colony <- tmp$remnant
colony
#> An object of class "Colony"
```

```
#> Id: 1
#> Location:
#> Queen: 2
#> Number of fathers: 15
#> Number of workers: 90
#> Number of drones: 10
#> Number of virgin queens: 0
#> Has split: FALSE
#> Has swarmed: FALSE
#> Has superseded: FALSE
#> Has collapsed: FALSE
#> Is productive: TRUE
```

```
pulledWorkers <- tmp$pulled
pulledWorkers
#> An object of class "Pop"
#> Ploidy: 2
#> Individuals: 10
#> Chromosomes: 3
#> Loci: 300
#> Traits: 0
```

Next, you can obtain the caste of each individual with the `getCaste()` function. As already mentioned above, a similar group of functions are the `is*()` functions that check whether an individual is of specific caste. Let's now obtain the caste of colony members:

```
getCaste(queen)
#> [1] "queen"
```

```
getCaste(fathers)
#>  [1] "fathers" "fathers" "fathers" "fathers" "fathers" "fathers" "fathers"
#>  [8] "fathers" "fathers" "fathers" "fathers" "fathers" "fathers" "fathers"
#> [15] "fathers"
```

and so on. When you have a collection of bees at hand and you might not know their source, the `getCaste()` can be very useful:

```
bees <- c(queen, fathers[1:2], workers[1:2], drones[1:2])
getCaste(bees)
#> [1] "queen"   "fathers" "fathers" "workers" "workers" "drones"  "drones"
```

Complementary sex determining locus

The complementary sex determiner (*CSD*) locus, well, complements sex determination. Fertilised eggs that are heterozygous at the *CSD* locus develop into workers. On the other hand, homozygous eggs develop into unviable drones. These drones are usually discarded by workers. SIMplyBee does not store these unviable drones, but it does store their number in the queen's miscellaneous slot (`queen@misc`). Here, you can find the total number of workers and drones produced by the queen (`nWorkers` and `nDrones`) and how many of the diploid offspring were homozygous at the *CSD* (`nHomBrood`). There is also a `pHomBrood` slot, that represents the theoretical (expected) proportion of offspring that are expected to be homozygous based on queen's and father's *CSD* alleles. You can obtain `pHomBrood` and `nHomBrood` values with the corresponding `pHomBrood()` and `nHombrood()` functions that can be applied either on the queen (`Pop` class) or colony (`Colony` class) directly. You can obtain the entire `misc` slot with the `getMisc()` function.

```r
getMisc(getQueen(colony))
#> [[1]]
#> [[1]]$fathers
#> An object of class "Pop"
#> Ploidy: 2
#> Individuals: 15
#> Chromosomes: 3
#> Loci: 300
#> Traits: 0
#>
#> [[1]]$nWorkers
#> [1] 100
#>
#> [[1]]$nDrones
#> [1] 10
#>
#> [[1]]$nHomBrood
#> [1] 0
#>
#> [[1]]$pHomBrood
#> [1] 0
```

Technically, in SIMplyBee we represent the *CSD* locus as a series of bi-allelic single nucleotide polymorphisms that don't recombine. So, the *CSD* locus is represented as a non-recombining haplotype and different haplotypes represent different *CSD* alleles. By varying the number of sites within the *CSD* locus we can control the number of distinct alleles (see `help(SimParamBee)`).

We can retrieve information about *CSD* alleles with `getCsdAlleles()`. For details on where the *CSD* locus is and the number of distinct alleles, see `help(SimParamBee)`. Looking at the below output, the first row shows marker identifications (chromosome_locus) and the first column shows haplotype identifications (individual_haplotype). The alleles are represented with a sequence of 0's and 1's. You can see that the two sequences are different, meaning that the queen is heterozygous, as expected.

```r
getCsdAlleles(queen)
#>     3_86 3_87 3_88 3_89 3_90
#> 2_1    1    1    0    0    1
#> 2_2    0    1    0    1    1
```

A keen geneticist would immediately inspect *CSD* alleles of fathers to check for any similarity with the queen's *CSD* alleles. Let's boost a chance of such an event by creating an inbreed colony. We will create a virgin queen from the current colony and mate her with her brothers. Oh, dear.

```r
inbredColony <- createColony(x = createVirginQueens(x = colony, nInd = 1))
fathers <- selectInd(drones, nInd = SP$nFathers, use = "rand")
#> Warning in selectInd(drones, nInd = SP$nFathers, use = "rand"): Suitable
#> candidates smaller than nInd, returning 10 individuals
inbredColony <- cross(inbredColony, drones = fathers)
getCsdAlleles(inbredColony)
#> $queen
#>       3_86 3_87 3_88 3_89 3_90
#> 255_1    0    1    0    1    1
#> 255_2    1    0    0    0    0
#>
#> $fathers
#>       3_86 3_87 3_88 3_89 3_90
```

```
#> 246_1    1    1    0    0    1
#> 249_1    0    1    0    1    1
#> 247_1    1    1    0    0    1
#> 254_1    0    1    0    1    1
#> 252_1    1    1    0    0    1
#> 250_1    1    1    0    0    1
#> 245_1    1    1    0    0    1
#> 253_1    1    1    0    0    1
#> 248_1    1    1    0    0    1
#> 251_1    0    1    0    1    1
#>
#> $workers
#> NULL
#>
#> $drones
#> NULL
#>
#> $virginQueens
#> NULL
getCsdAlleles(inbredColony, unique = TRUE)
#> $queen
#>       3_86 3_87 3_88 3_89 3_90
#> 255_1    0    1    0    1    1
#> 255_2    1    0    0    0    0
#>
#> $fathers
#>       3_86 3_87 3_88 3_89 3_90
#> 252_1    1    1    0    0    1
#> 249_1    0    1    0    1    1
#>
#> $workers
#> NULL
#>
#> $drones
#> NULL
#>
#> $virginQueens
#> NULL
```

Can you spot any matches? Let's calculate the expected proportion of homozygous brood from this mating.

```
pHomBrood(inbredColony)
#> [1] 0.15
```

Let's see how many homozygotes will we observe. Note that inheritance is a random process, so a realised number of homozygotes will deviate from the expected proportion.

```
inbredColony <- addWorkers(inbredColony, nInd = 100)
inbredColony
#> An object of class "Colony"
#> Id: 2
#> Location:
#> Queen: 255
#> Number of fathers: 10
#> Number of workers: 80
```

```
#> Number of drones: 0
#> Number of virgin queens: 0
#> Has split: FALSE
#> Has swarmed: FALSE
#> Has superseded: FALSE
#> Has collapsed: FALSE
#> Is productive: FALSE
nHomBrood(inbredColony)
#> [1] 20
```

We tried adding 100 workers, but we only got 80. The difference of 20 is due to *CSD* homozygous brood. Let's add another set of workers to show variation in the realised numbers and accumulation of information.

```
inbredColony <- addWorkers(inbredColony, nInd = 100)
inbredColony
#> An object of class "Colony"
#> Id: 2
#> Location:
#> Queen: 255
#> Number of fathers: 10
#> Number of workers: 171
#> Number of drones: 0
#> Number of virgin queens: 0
#> Has split: FALSE
#> Has swarmed: FALSE
#> Has superseded: FALSE
#> Has collapsed: FALSE
#> Is productive: FALSE
nHomBrood(inbredColony)
#> [1] 29
```

In total we tried adding 200 workers. We got 171 workers and 29 homozygous brood. To see all this information, we can inspect the miscellaneous slot of the queen that contains the fathers population as well as the cumulative number of workers, drones, homozygous brood, and the expected proportion of homozygous brood.

```
getMisc(getQueen(inbredColony))
#> [[1]]
#> [[1]]$fathers
#> An object of class "Pop"
#> Ploidy: 2
#> Individuals: 10
#> Chromosomes: 3
#> Loci: 300
#> Traits: 0
#>
#> [[1]]$nWorkers
#> [1] 171
#>
#> [[1]]$nDrones
#> [1] 0
#>
#> [[1]]$nHomBrood
#> [1] 29
#>
```

```
#> [[1]]$pHomBrood
#> [1] 0.15
```
