# Supplementary material for: SIMplyBee: an R package to simulate honeybee populations and breeding programs
Source: Genet Sel Evol. 2023 May 9;55:31. doi: 10.1186/s12711-023-00798-y (PMC10169377; doi:10.1186/s12711-023-00798-y)
Supplement: Supplementary file 2 — Additional file 2. Multiple colonies vignette. This vignette introduces working with multiple colonies bydemonstrating how to create and work with MultiColony objects inSIMplyBee. This vignette can also be found on https://cran.r-project.org/package=SIMplyBee and http://www.SIMplyBee.info. [file 12711_2023_798_MOESM2_ESM.pdf]

Additional file 2 - Multiple colonies vignette

2023-03-21

Introduction

We have already introduced the Colony class that holds colony-specific information and caste individuals. However, when working with honeybees, we usually do not work with a single colony, but with apiaries or even whole populations of colonies. To cater for this, SIMplyBee provides a `MultiColony` class. It behaves as a list of `Colony` objects but with additional functionality - you can apply function directly to the `MultiColony` objects. A `MultiColony` can represent different apiaries or sub-populations in terms of either age of the queens or geographical location of the apiaries etc. This vignette demonstrates creating and working with `MultiColony` objects. First, we again load the package.

```
library(package = "SIMplyBee")
#> Loading required package: AlphaSimR
#> Loading required package: R6
#>
#> Attaching package: 'SIMplyBee'
#> The following object is masked from 'package:base':
#>
#>     split
```

Creating a MultiColony object

We create a `MultiColony` object with `createMultiColony()` function. Let's say you want to create a `MultiColony` object that represents a single apiary. The first option is to initialise an empty `MultiColony` object that represents an empty apiary without any colonies and individuals within them.

```
# Create an empty apiary
emptyApiary <- createMultiColony()
emptyApiary
#> An object of class "MultiColony"
#> Number of colonies: 0
#> Are empty: 0
#> Are NULL: 0
#> Have split: 0
#> Have swarmed: 0
#> Have superseded: 0
#> Have collapsed: 0
#> Are productive: 0
```

Let's inspect the printout of the `MultiColony` object. This tells how many colonies are within, how many of them are `empty` and contain no individuals, how many are `NULL` objects, how many have experienced a split, swarm, supersedure, or a collapse (you can read more about these events in the Colony events vignette), and how many of them are productive, meaning that we can collect a production phenotype from them such as honey yield.

The second option is again to create an empty `MultiColony` object that represents an empty apiary without any individuals within, but with a defined number of colony slots.

```r
# Create an empty apiary with 10 colony slots
emptyApiary1 <- createMultiColony(n = 10)
emptyApiary1
#> An object of class "MultiColony"
#> Number of colonies: 10
#> Are empty: 10
#> Are NULL: 10
#> Have split: 0
#> Have swarmed: 0
#> Have superseded: 0
#> Have collapsed: 0
#> Are productive: 0
```

The third option is to create a `MultiColony` object with a population of either virgin or mated queens. For this, we first have to initialise the simulation with founder genomes and creating a base population of virgin queens. We will use 10 virgin queens to produce drones and create a DCA.

```r
# Create 20 founder genomes
founderGenomes <- quickHaplo(nInd = 30, nChr = 1, segSites = 100)
# Set up new global simulation parameters
SP <- SimParamBee$new(founderGenomes)
# Create a base population of 20 virgin queens
basePop <- createVirginQueens(founderGenomes)
# Create a DCA from the drones of the first 10 queens
DCA <- createDrones(basePop[1:10], nInd = 100)
```

We will now create an apiary with 10 virgin colonies with the `createMultiColony()` function by providing the second set of 10 virgin queens as the input parameter. Let's call this apiary `apiary1` and say that it is positioned at the location `(1,1)`.

```r
# Create an apiary with the remaining virgin queens
apiary1 <- createMultiColony(x = basePop[11:20])
# Set the location of the apiary
apiary1 <- setLocation(apiary1, c(1,1))
```

Let's now use functions `isQueenPresent()` and `isVirginQueensPresent()` to confirm all the colonies are virgin.

```r
# Check whether all the colonies are virgin
isQueenPresent(apiary1)
#>     1     2     3     4     5     6     7     8     9    10
#> FALSE FALSE FALSE FALSE FALSE FALSE FALSE FALSE FALSE FALSE
isVirginQueensPresent(apiary1)
#>    1    2    3    4    5    6    7    8    9   10
#> TRUE TRUE TRUE TRUE TRUE TRUE TRUE TRUE TRUE TRUE
```

MultiColony operations

Once we have a non-empty `MultiColony` object, we can do basic operations on it. First, we can select some colonies by either specifying their IDs, desired number or percentage of randomly selected colonies.

```r
# Get the IDs of the colonies
getId(apiary1)
#>  [1]  1  2  3  4  5  6  7  8  9 10
# Select colonies according to IDs
selectColonies(apiary1, ID = c(1,2))
```

```
#> An object of class "MultiColony"
#> Number of colonies: 2
#> Are empty: 0
#> Are NULL: 0
#> Have split: 0
#> Have swarmed: 0
#> Have superseded: 0
#> Have collapsed: 0
#> Are productive: 0
# Randomly select a given percentage of colonies
selectColonies(apiary1, p = 0.1)
#> Randomly selecting colonies: 1
#> An object of class "MultiColony"
#> Number of colonies: 1
#> Are empty: 0
#> Are NULL: 0
#> Have split: 0
#> Have swarmed: 0
#> Have superseded: 0
#> Have collapsed: 0
#> Are productive: 0
```

Second, we can pull some colonies from the `MultiColony` object. This means, that the pulled colonies are removed from the original object. The function `pullColonies()` therefore returns two object - the pulled colonies and the remnant colonies.

```
# Pull one colony - returns a list with $remnant and $pulled nodes
pullColonies(apiary1, n = 1)
#> Randomly pulling colonies: 1
#> $pulled
#> An object of class "MultiColony"
#> Number of colonies: 1
#> Are empty: 0
#> Are NULL: 0
#> Have split: 0
#> Have swarmed: 0
#> Have superseded: 0
#> Have collapsed: 0
#> Are productive: 0
#>
#> $remnant
#> An object of class "MultiColony"
#> Number of colonies: 9
#> Are empty: 0
#> Are NULL: 0
#> Have split: 0
#> Have swarmed: 0
#> Have superseded: 0
#> Have collapsed: 0
#> Are productive: 0
```

Third, we can also remove some colonies from the `MultiColony` object with `removeColonies()` function.

```
removeColonies(apiary1, ID = 13)
#> Warning in removeColonies(apiary1, ID = 13): ID parameter contains come invalid
```

```
#> IDs!
#> An object of class "MultiColony"
#> Number of colonies: 10
#> Are empty: 0
#> Are NULL: 0
#> Have split: 0
#> Have swarmed: 0
#> Have superseded: 0
#> Have collapsed: 0
#> Are productive: 0
```

These three functions can also select, pull, and remove colonies based on some values (phenotypes, genetic values ...). You can read more about that in the Quantitative genetics vignette.

Crossing a MultiColony

Next, we will cross all the virgin queens in the apiary with the `cross()` function to groups of drones that we collected from the DCA with the `pullDroneGroupsFromDCA()` function. We have to collect at least as many groups of drones as we have colonies in our `MultiColony`.

```
# Pull 10 groups of drones from the DCA
droneGroups <- pullDroneGroupsFromDCA(DCA, n = 10, nDrones = nFathersPoisson)
# Cross all virgin queens in the apiary to the selected drones
apiary1 <- cross(apiary1, drones = droneGroups)
# Check whether the queens are present (and hence mated)
isQueenPresent(apiary1)
#>    1    2    3    4    5    6    7    8    9   10
#> TRUE TRUE TRUE TRUE TRUE TRUE TRUE TRUE TRUE TRUE
```

Once we have mated queens in the apiary, we can apply all the event functions directly to the `MultiColony` object: `buildUp()`, `downsize()`, `swarm()`, `split()`, `supersede()`, `collapse()` but also all the functions that either add, replace, or remove individuals from the castes. Let's say we want to build-up all the colonies in our apiary.

```
# Build-up all the colonies in the apiary1
apiary1 <- buildUp(apiary1, nWorkers = 1000, nDrones = 100)
```

Furthermore, we can use the `pullColonies()` or `selectColonies()` to subset the colonies that will for example swarm, collapse, or supersede (presented in the Colony events vignette), or the ones that we decided to split (check out the Colony events vignette).

Working with multiple MultiColony objects

Let's now initiate another `MultiColony` named as `apiary2` that is placed at location `(2,2)`. Here, we define different `MultiColony` object according to the location of the apiary, but the objects could also be defined according to the age of the queens (such as `age0`, `age1`...). `apiary2` contains only virgin queens and we want to mate them to a DCA made of drones from `apiary1`.

```
# Initiate apiary2 at the location (2,2)
apiary2 <- createMultiColony(basePop[21:30])
apiary2 <- setLocation(apiary2, c(2,2))
```

Since some time has passed, we want to first replace the drones in `apiary1` with new drones. We can do that with `replaceDrones()` function.

```r
apiary1 <- replaceDrones(apiary1)
```

Now that we have a new set of drones, we can create a DCA with the function `createDCA()` and mate virgin queens in apiary2 to the DCA.

```r
# Check whether all colonies in apiary2 are virgin
isQueenPresent(apiary2)
#>    11    12    13    14    15    16    17    18    19    20
#> FALSE FALSE FALSE FALSE FALSE FALSE FALSE FALSE FALSE FALSE
isVirginQueensPresent(apiary2)
#>   11   12   13   14   15   16   17   18   19   20
#> TRUE TRUE TRUE TRUE TRUE TRUE TRUE TRUE TRUE TRUE
# Create a DCA from all the drones in apiary
DCA <- createDCA(apiary1)
# Check how big is the DCA
DCA
#> An object of class "Pop"
#> Ploidy: 2
#> Individuals: 1000
#> Chromosomes: 1
#> Loci: 100
#> Traits: 0
# Sample drones groups from the DCA
droneGroups <- pullDroneGroupsFromDCA(DCA,
                                      n = nColonies(apiary2),
                                      nDrones = nFathersPoisson)
# Cross virgin queens in apiary2 to selected drones
apiary2 <- cross(apiary2, drones = droneGroups)
```

To learn more about the `nFathersPoisson()` function and other similar functions, read the Sampliong functions vignette.
