# Supplementary material for: SIMplyBee: an R package to simulate honeybee populations and breeding programs
Source: Genet Sel Evol. 2023 May 9;55:31. doi: 10.1186/s12711-023-00798-y (PMC10169377; doi:10.1186/s12711-023-00798-y)
Supplement: Supplementary file 3 — Additional file 3. Colony events vignette. This vignette introduces the colony events and how tosimulate them in SIMplyBee. It shows how to simulate swarming, splitting,superseding, and collapsing either a Colony or MultiColony objects[59–61]. This vignette can also be found on https://cran.r-project.org/package=SIMplyBee and http://www.SIMplyBee.info. [file 12711_2023_798_MOESM3_ESM.pdf]

Additional file 3 - Colony events vignette

2023-03-24

Introduction

This vignette will introduce you to honeybee colony events. SIMplyBee implements the most important natural events like swarming, supersedure, and collapse of the colony, and a beekeeping management practice called splitting. All functions that implement colony events work both on `Colony` and `MultiColony` objects.

First, we start by loading the package.

```
library(package = "SIMplyBee")
#> Loading required package: AlphaSimR
#> Loading required package: R6
#>
#> Attaching package: 'SIMplyBee'
#> The following object is masked from 'package:base':
#>
#>     split
```

The code below generates 6 colonies so we can demonstrate various colony events.

```
founderGenomes <- quickHaplo(nInd = 30, nChr = 1, segSites = 100)
SP <- SimParamBee$new(founderGenomes)
basePop <- createVirginQueens(founderGenomes)
drones <- createDrones(basePop[1:10], n = 1000)
fatherGroups <- pullDroneGroupsFromDCA(drones, n = 30, nDrones = 10)

# Create Colony and MultiColony class, cross them and build them up
colony <- createColony(x = basePop[11])
colony <- cross(colony, drones = fatherGroups[[1]])
colony <- buildUp(colony, nWorkers = 100, nDrones = 20)

apiary <- createMultiColony(basePop[12:17])
apiary <- cross(apiary, drones = fatherGroups[2:7])
apiary <- buildUp(apiary, nWorkers = 100, nDrones = 20, exact = TRUE)
```

Swarming

Swarming is the process through which honeybee colonies produce new colonies. When a honeybee colony outgrows its hive, becomes too congested, or too populated for the queen's pheromones to spread among workers, then the swarming begins. The workers start building swarm cells for new virgin queens. When the queen is ready, she leaves the hive and is followed by about 70% of the workers in a massive cloud of flying bees, the swarm [59]. The swarm will cluster on a nearby tree or bush and remain there until they find a suitable new home.

The virgin queens developing in the old hive are daughters of the queen that swarmed and are attended by the remnant workers that did not leave with the swarm. After few days, the new virgin queens begin to emerge. Typically, the first queen to emerge will kill the rest of virgin queens to assume the role as the new

queen for the colony. She will then go on a mating flight to find drones to mate with to begin laying eggs and rebuilding the workforce in the colony [59, 60].

In SIMplyBee, function `swarm()` simulates swarming (Figure 1). The function takes a `Colony` class object and a percentage `p` of workers that leave with the swarm. The function returns a list with two `Colony` class objects, `swarm` and `remnant`. The `swarm` contains the old queen and `p` percentage of workers that left the hive. The `remnant` contains the rest of workers `(1-p)`, all the drones, and virgin queens that are daughters of the old queen that swarmed.

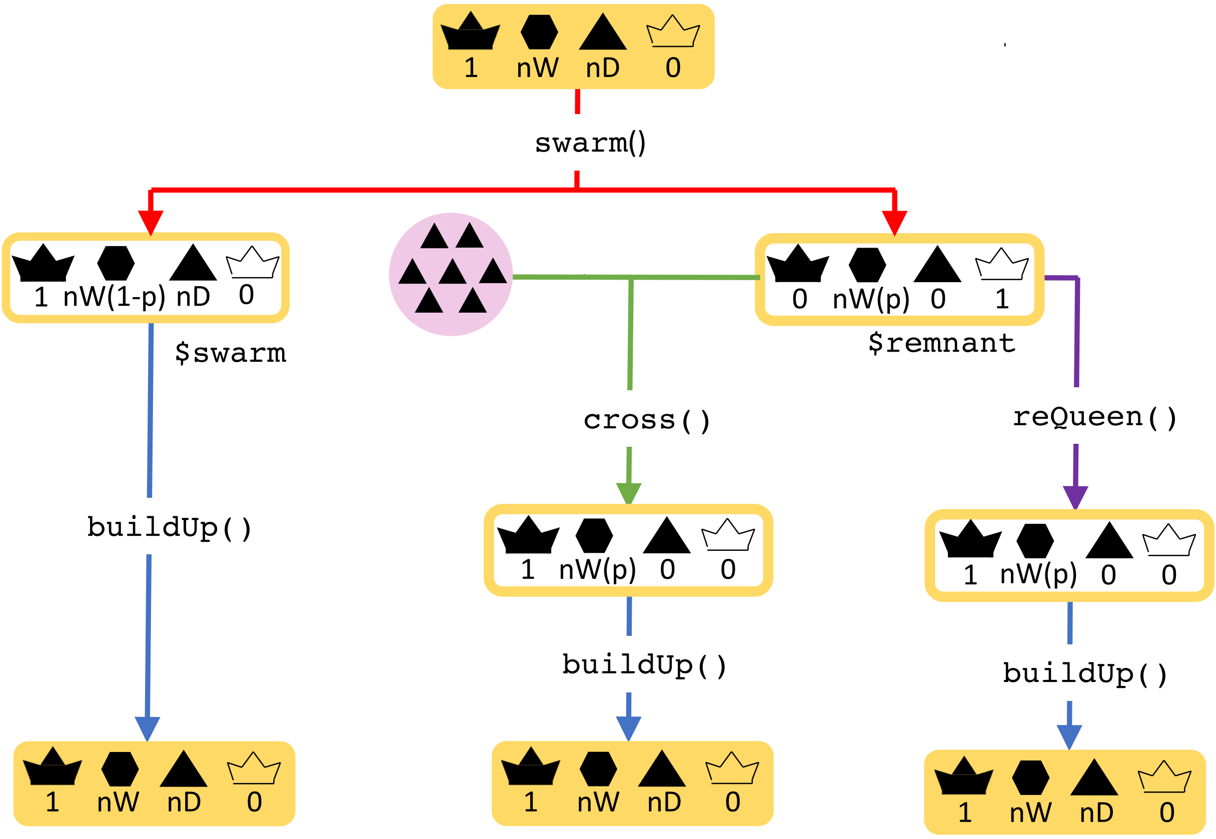

Figure 1: Swarm function.

- Swarming a Colony

Let's swarm our `colony`:

```
tmp <- swarm(colony, p = 0.4)
tmp
#> $swarm
#> An object of class "Colony"
#> Id: 8
#> Location:
#> Queen: 11
#> Number of fathers: 10
#> Number of workers: 36
#> Number of drones: 0
```

```
#> Number of virgin queens: 0
#> Has split: FALSE
#> Has swarmed: TRUE
#> Has superseded: FALSE
#> Has collapsed: FALSE
#> Is productive: FALSE
#>
#> $remnant
#> An object of class "Colony"
#> Id: 9
#> Location:
#> Queen: NA
#> Number of fathers: 0
#> Number of workers: 54
#> Number of drones: 20
#> Number of virgin queens: 1
#> Has split: FALSE
#> Has swarmed: TRUE
#> Has superseded: FALSE
#> Has collapsed: FALSE
#> Is productive: FALSE
```

We see that the remnant colony does not have a queen but has one virgin queen that will have to be mated. It also has 60 workers since we set `p` argument to 0.4, meaning that 40% of workers left with the swarm. All the drones remained in the remnant. Note that the `swarm` status is turned to `TRUE`.

The swarm contains the old queen, no virgin queens, and 40 workers, since we set the proportion `p` to 0.4. Same as in the remnant, the `swarm` status is turned to `TRUE` in the swarm.

Swarming also turns the `production` slot to `FALSE` for both, the swarm and the remnant colony, since we would not be able to collect honey from such colonies (or other products).

The swarm stays genetically identical to the "old" colony, although downsized. Assuming that we have caught the swarm, we assign it back to the original colony object. The remnant has a new queen and is hence genetically different from the original colony. Thus, we assigned it to a new colony object.

```
colony <- tmp$swarm
colony1 <- tmp$remnant
```

After swarming, the colony would usually build-up back to the full size and the virgin queens would mate. Building-up a colony turns the production status back to `TRUE`.

```
colony <- buildUp(colony)
colony
#> An object of class "Colony"
#> Id: 8
#> Location:
#> Queen: 11
#> Number of fathers: 10
#> Number of workers: 89
#> Number of drones: 100
#> Number of virgin queens: 0
#> Has split: FALSE
#> Has swarmed: TRUE
#> Has superseded: FALSE
#> Has collapsed: FALSE
```

```
#> Is productive: TRUE
```

Instead of setting the `p` every time we call the `swarm()` function, we can save the `swarmP` argument in the `SimParamBee` object. The `swarm()` function will then use this percentage if `p` is not set.

```
SP$swarmP
#> [1] 0.5
```

The default value is 0.5, but we can set any value we want.

```
SP$swarmP <- 0.35
SP$swarmP
#> [1] 0.35
```

You can also use a non-fixed `p` parameter by using the function `swarmPUnif` that samples the `p` from a uniform distribution between values 0.4 and 0.6 irrespective of the colony strength. You can read more about this in the Sampling functions vignette.

- Swarming a MultiColony

We swarm a `MultiColony` object in the same was we swarm a single `Colony` - with the `swarm()` function. The `swarm()` function is here applied to each colony in the `MultiColony` object with the same parameters. The function now returns a list with two `MultiColony` objects - one containing the swarms and the other containing the remnants.

```
tmp <- swarm(apiary)
tmp
#> $swarm
#> An object of class "MultiColony"
#> Number of colonies: 6
#> Are empty: 0
#> Are NULL: 0
#> Have split: 0
#> Have swarmed: 6
#> Have superseded: 0
#> Have collapsed: 0
#> Are productive: 0
#>
#> $remnant
#> An object of class "MultiColony"
#> Number of colonies: 6
#> Are empty: 0
#> Are NULL: 0
#> Have split: 0
#> Have swarmed: 6
#> Have superseded: 0
#> Have collapsed: 0
#> Are productive: 0
```

We see that we get six swarms and six remnants from the apiary with six colonies. We can inspect individuals colonies to ensure they swarmed according to the parameters. Let's inspect the swarm and remnant of the third colony.

```
tmp$swarm[[3]]
#> An object of class "Colony"
#> Id: 14
#> Location:
#> Queen: 14
```

```
#> Number of fathers: 10
#> Number of workers: 35
#> Number of drones: 0
#> Number of virgin queens: 0
#> Has split: FALSE
#> Has swarmed: TRUE
#> Has superseded: FALSE
#> Has collapsed: FALSE
#> Is productive: FALSE
```

```
tmp$remnant[[3]]
#> An object of class "Colony"
#> Id: 15
#> Location:
#> Queen: NA
#> Number of fathers: 0
#> Number of workers: 65
#> Number of drones: 20
#> Number of virgin queens: 1
#> Has split: FALSE
#> Has swarmed: TRUE
#> Has superseded: FALSE
#> Has collapsed: FALSE
#> Is productive: FALSE
```

We see that the the third colony was swarmed with `p` of 35% as specified in the `SimParamBee`, hence the swarm left with 35 workers and the old queen, and the remnant stayed behind with a new virgin queen and 65 workers.

Above, all the colonies in a `MultiColony` are swarmed with the same percentage. However, we can also specify a different `p` for each colony.

```
tmp <- swarm(apiary, p = c(0.3, 0.4, 0.5, 0.6, 0.7, 0.8))
```

If we now inspect the first and the second swarm, we see that each colony has a different percentage of workers that stayed or left.

```
tmp$swarm[[1]]
#> An object of class "Colony"
#> Id: 22
#> Location:
#> Queen: 12
#> Number of fathers: 10
#> Number of workers: 30
#> Number of drones: 0
#> Number of virgin queens: 0
#> Has split: FALSE
#> Has swarmed: TRUE
#> Has superseded: FALSE
#> Has collapsed: FALSE
#> Is productive: FALSE
```

```
tmp$swarm[[3]]
#> An object of class "Colony"
#> Id: 26
#> Location:
```

```
#> Queen: 14
#> Number of fathers: 10
#> Number of workers: 50
#> Number of drones: 0
#> Number of virgin queens: 0
#> Has split: FALSE
#> Has swarmed: TRUE
#> Has superseded: FALSE
#> Has collapsed: FALSE
#> Is productive: FALSE
```

If you want to track the genetics, you might want to assign the swarms back to the original apiary and create a new apiary from the remnants. However, if you want to track the position, the remnant actually stay in the original position and would hence be assigned back to the same apiary, while the swarm would be assigned to a new apiary or even be lost. Here, we will track the genetics and assign the swarm back to the original apiary and remnants to a new apiary.

```
apiary <- tmp$swarm
apiary <- buildUp(apiary)
apiary1 <- tmp$remnant
```

Split

Colony splitting is a common beekeeping technique to limit swarming. A percentage of workers, brood, and food stores are split away to create a new colony or combine two split. Old queen normally stays in the original (remnant) colony. We created a function `split()` that works on `Colony` and `Multicolony` objects (Figure 2). It accepts the `p` argument as a proportion of workers that will be split away for a new colony. The output of the function is a list of two `Colony` or `MultiColony` objects: remnant that contains the old queen, drones, and `(1-p)` workers; and split that doesn't contain a queen, but contains virgin queens and `p` workers. The `split()` function follows the same principles as the `swarm()`, hence we will limit explaining the outputs.

- Splitting a Colony

```
tmp <- split(colony, p = 0.3)
tmp
#> $split
#> An object of class "Colony"
#> Id: 34
#> Location:
#> Queen: NA
#> Number of fathers: 0
#> Number of workers: 27
#> Number of drones: 0
#> Number of virgin queens: 1
#> Has split: TRUE
#> Has swarmed: FALSE
#> Has superseded: FALSE
#> Has collapsed: FALSE
#> Is productive: FALSE
#>
#> $remnant
#> An object of class "Colony"
#> Id: 8
#> Location:
```

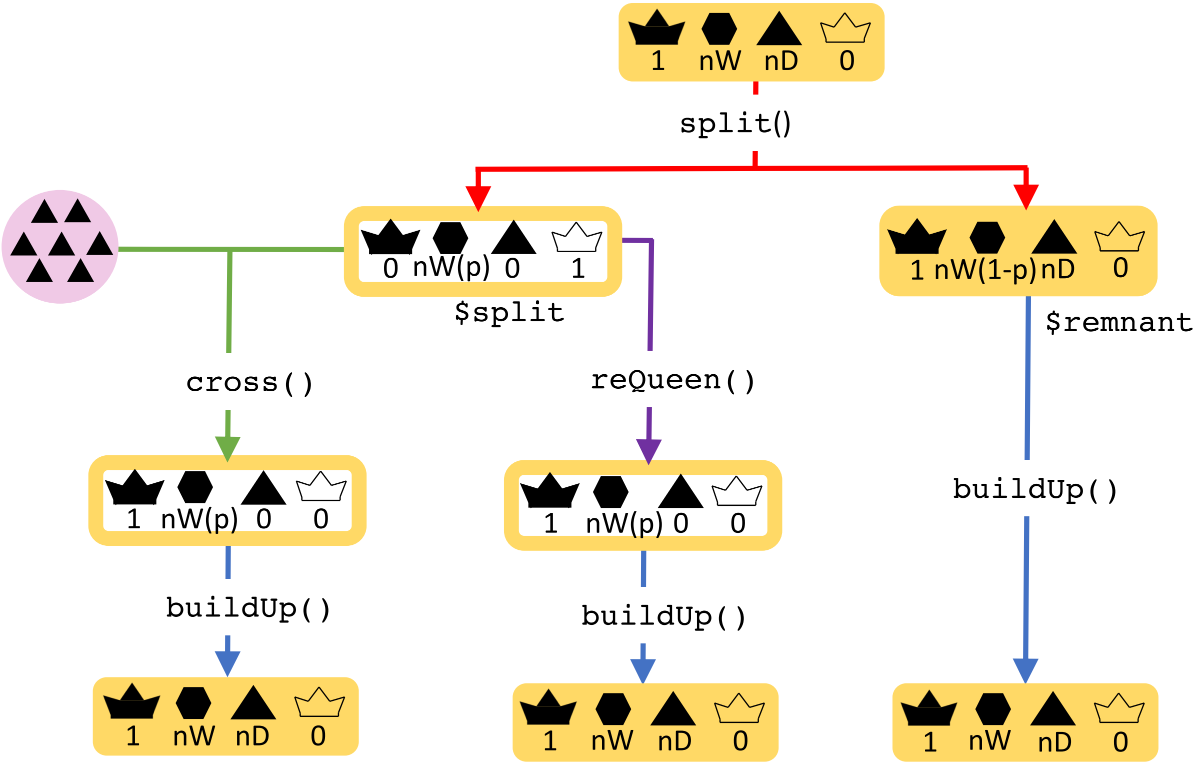

Figure 2: Split function

```
#> Queen: 11
#> Number of fathers: 10
#> Number of workers: 62
#> Number of drones: 100
#> Number of virgin queens: 0
#> Has split: TRUE
#> Has swarmed: TRUE
#> Has superseded: FALSE
#> Has collapsed: FALSE
#> Is productive: TRUE
```

We see that remnant contains the old queen and 70% of workers since we set `p` argument to 0.3, meaning that 30% of workers are removed in the split. The `split` status of the remnant colony is turned to `TRUE`. The `production` status of the remnant stays `TRUE`, because in reality we always split a colony in a way that does not threaten the production of the remnant colony.

If we inspect the split, we see that it contains 30% of the workers, the `split` status is turned to `TRUE`, and the `production` status is turned to `FALSE`, since in reality, these small colonies would not be productive.

We would usually consider the remnant as the original colony, since it tracks its genetics and location.

```
colony <- tmp$remnant
colony
#> An object of class "Colony"
#> Id: 8
#> Location:
#> Queen: 11
#> Number of fathers: 10
#> Number of workers: 62
#> Number of drones: 100
#> Number of virgin queens: 0
#> Has split: TRUE
#> Has swarmed: TRUE
#> Has superseded: FALSE
#> Has collapsed: FALSE
#> Is productive: TRUE
```

After a split, the colony would build-up back to their full-size.

```
colony <- buildUp(colony)
```

The `p` argument for splitting can be also saved in `SP` object, so we do not specify it each time we call the function - see `SimParamBee$splitP`.

- Splitting a MultiColony

We split a `MultiColony` object in the same way we split a `Colony` (as shown in swarm).

```
tmp <- split(apiary, p = 0.3)
tmp$remnant[[1]]
#> An object of class "Colony"
#> Id: 22
#> Location:
#> Queen: 12
#> Number of fathers: 10
#> Number of workers: 70
#> Number of drones: 100
#> Number of virgin queens: 0
```

```
#> Has split: TRUE
#> Has swarmed: TRUE
#> Has superseded: FALSE
#> Has collapsed: FALSE
#> Is productive: TRUE
```

We again see that in remnant we have the queen and 70 workers:

```
tmp$split[[1]]
#> An object of class "Colony"
#> Id: 35
#> Location:
#> Queen: NA
#> Number of fathers: 0
#> Number of workers: 30
#> Number of drones: 0
#> Number of virgin queens: 1
#> Has split: TRUE
#> Has swarmed: FALSE
#> Has superseded: FALSE
#> Has collapsed: FALSE
#> Is productive: FALSE
```

and a virgin queen with 30 workers in split. We can use the vector of `p`, different for each colony same as shown for the `swarm()` function above.

After the split, we would assign the remnant colonies back to the apiary and build them up.

```
apiary <- tmp$remnant
apiary <- buildUp(apiary)
```

Supersedure

Supersedure is a replacement of the queen by one of her daughters without interference of the beekeeper. Supersedure is a natural way of re-queening a colony without swarming. There are many reasons for supersedure: poor physical condition of a queen, old age, diseases, depleted spermatheca, poorly bread queen, reduced pheromone output and many others [61].

Function `supersede()` removes the old queen and triggers the creation of new virgin queens from the brood (Figure 3). The function returns a single `Colony` or `MultiColony` object (not a list of two).

- Superseding a Colony

```
colony <- supersede(colony)
colony
#> An object of class "Colony"
#> Id: 8
#> Location:
#> Queen: NA
#> Number of fathers: 0
#> Number of workers: 90
#> Number of drones: 100
#> Number of virgin queens: 1
#> Has split: TRUE
#> Has swarmed: TRUE
#> Has superseded: TRUE
```

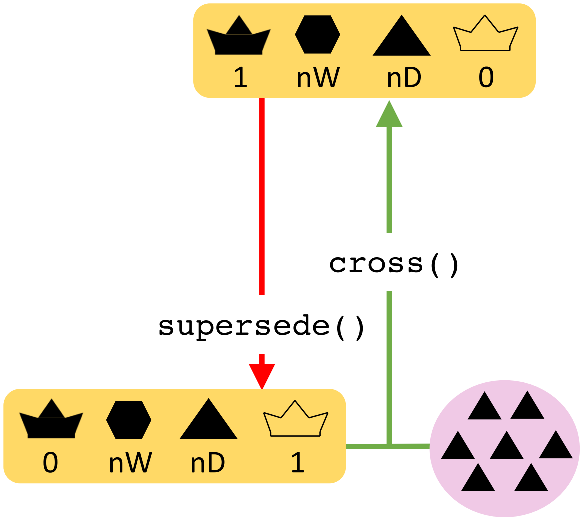

Figure 3: Supersede function

```
#> Has collapsed: FALSE
#> Is productive: TRUE
```

We see that after a supersedure, the old queen is removed and a new virgin queen is ready to mate. Hence, the next step in the simulation would be to cross this virgin queen. We also see that the `supersedure` status is set to `TRUE`. The `production` status stays set to `TRUE`, since the colony did not loose any individuals (it stayed at it's full-size).

- Superseding a MultiColony

The function `supersede()` works both on `Colony` and `MultiColony` classes. We supersede a `MultiColony` in a same way as a `Colony`.

```
apiary <- supersede(apiary)
apiary
#> An object of class "MultiColony"
#> Number of colonies: 6
#> Are empty: 0
#> Are NULL: 0
#> Have split: 6
#> Have swarmed: 6
#> Have superseded: 6
#> Have collapsed: 0
#> Are productive: 6
```

Collapse

Collapse of the colony is a term that describes the death of a colony - when all individuals within a colony die. There are many reasons for the collapse of a honey bee colony: diseases, starvation, queen problems, contamination with pesticides, etc. Colony losses can be high, possibly up to 60% of colonies per year. High

colony losses can significantly influence genetic structure of a population and hence genetic diversity and genetic gain.

Function `collapse()` simulates the collapse of a colony (Figure 4). This function does not remove individuals from the colony, but sets the `collapse` status of the colony to `TRUE` and `production` status to `FALSE`. This is to mimic reality, where bees would still be present in the colony, although being dead. This allows us to extract any genetic material from the colony even after collapse, say to study genetic causes related to the collapse. Future operations in terms of reproduction or simulation of events are not allowed with a collapsed colony.

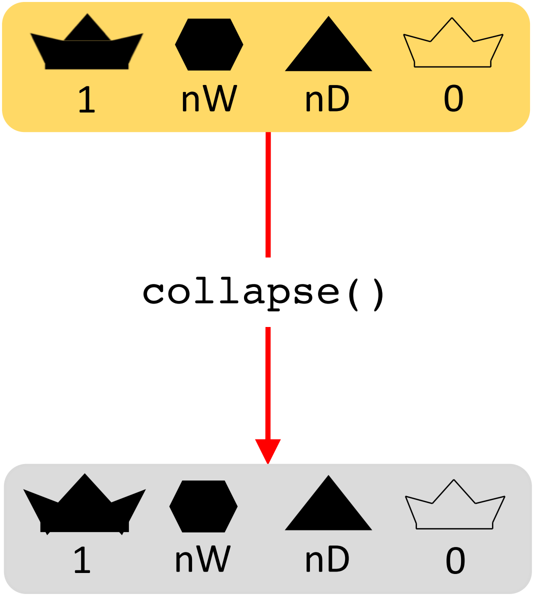

Figure 4: Collapse function

- Collapsing a Colony

```
colony <- collapse(colony)
colony
#> An object of class "Colony"
#> Id: 8
#> Location:
#> Queen: NA
#> Number of fathers: 0
#> Number of workers: 90
#> Number of drones: 100
#> Number of virgin queens: 1
#> Has split: TRUE
#> Has swarmed: TRUE
#> Has superseded: TRUE
#> Has collapsed: TRUE
#> Is productive: FALSE
```

- Collapsing a MultiColony

`collapse()` function is used when you want to keep collapsed colonies for subsequent analyses. If you don't need the collapsed colony, you can also simply select the surviving colonies with the `selectColonies()` function.

```r
apiary <- collapse(apiary)
apiary[[3]]
#> An object of class "Colony"
#> Id: 26
#> Location:
#> Queen: NA
#> Number of fathers: 0
#> Number of workers: 100
#> Number of drones: 100
#> Number of virgin queens: 1
#> Has split: TRUE
#> Has swarmed: TRUE
#> Has superseded: TRUE
#> Has collapsed: TRUE
#> Is productive: FALSE
apiary
#> An object of class "MultiColony"
#> Number of colonies: 6
#> Are empty: 0
#> Are NULL: 0
#> Have split: 6
#> Have swarmed: 6
#> Have superseded: 6
#> Have collapsed: 6
#> Are productive: 0
```
