# Supplementary material for: SIMplyBee: an R package to simulate honeybee populations and breeding programs
Source: Genet Sel Evol. 2023 May 9;55:31. doi: 10.1186/s12711-023-00798-y (PMC10169377; doi:10.1186/s12711-023-00798-y)
Supplement: Supplementary file 4 — Additional file 4. Crossing vignette. This vignette demonstrated how to cross virgin queens inSIMplyBee. It demonstrates how to cross a single or multiple virgin queens,cross either with pre-selected population/group of drones or according to across plan, and cross queens on an open DCA or mating station. This vignette can also be found on https://cran.r-project.org/package=SIMplyBee and http://www.SIMplyBee.info. [file 12711_2023_798_MOESM4_ESM.pdf]

Additional file 4 - Crossing vignette

2023-03-21

Introduction

This vignette shows how you can cross virgin queens in SIMplyBee. Here, we present how you can cross:

- single or multiple virgin queens (class `Pop`), virgin queen in a colony (class `Colony`), or all the virgin queens in an apiary or population (class `MultiColony`);
- cross either with pre-selected population of drones or according to a cross plan, and
- cross queens at an open drone congregation area (DCA) or at a mating station.

Start by loading the package:

```
library(package = "SIMplyBee")
#> Loading required package: AlphaSimR
#> Loading required package: R6
#>
#> Attaching package: 'SIMplyBee'
#> The following object is masked from 'package:base':
#>
#>     split
```

First, we create a founder population and some virgin queen, virgin colonies, and virgin apiaries that we will later cross.

```
# Simulate 40 founder genomes
founderGenomes <- quickHaplo(nInd = 50, nChr = 1, segSites = 100)
# Set global population paramaters
SP <- SimParamBee$new(founderGenomes)
# Create a base population of 40 virgin queens
basePop <- createVirginQueens(founderGenomes)

# Prepare populations with a single virgin queen
virginQueen1 <- basePop[1]
virginQueen2 <- basePop[2]
virginQueen3 <- basePop[3]
# Prepare populations with multiple virgin queens
virginQueens1 <- basePop[4:6]
virginQueens2 <- basePop[7:9]
virginQueens3 <- basePop[10:12]
# Prepare virgin Colony objects
colony1 <- createColony(basePop[13])
colony2 <- createColony(basePop[14])
colony3 <- createColony(basePop[15])
colony4 <- createColony(basePop[16])
# Prepare virgin MultiColony objects
apiary1 <- createMultiColony(basePop[17:21])
apiary2 <- createMultiColony(basePop[22:26])
```

```
apiary3 <- createMultiColony(basePop[27:31])
apiary4 <- createMultiColony(basePop[32:41])
```

We will now create a groups of drones from the remaining queens with 1,000 drones per queen that will represent a drone congregation area (DCA).

```
# Create a DCA from the remaining virgin queens
DCA <- createDrones(basePop[42:50], nInd = 1000)
```

Cross virgin queens at an open DCA

Cross by pre-selecting drone populations

We start by crossing our populations and colonies to pre-selected populations of drones. We pre-select the groups by pulling a desired number of drone packages from a DCA with the function `pullDroneGroupsFromDCA()`. This function requires you to specify a group of drones (`DCA`), how many groups you want to pull from the DCA (`n`), and how many drones per group you want (`nDrones`). For `nDrones`, you can either specify an integer or a sampling function, which results in a different number of drones in each of the pulled groups (you can read more about this in the Sampling functions vignette). These sampling functions are particularly useful in crossing simulations:

- `nFathersPoisson()`: samples the number of drones from a Poisson distribution with a default mean of 15 (the user can specify a different mean) - the output can contain the value 0 and
- `nFathersTruncPoisson()`: samples the number of drones from a zero truncated Poisson distribution with a default mean of 15 (the user can specif a different mean) - the output does not contain the value 0.

If these functions do not satisfy your needs, you can specify your own sampling function(s).

We can pull the drone groups out separately for each crossing or pull them out all at once.

```
# Pre-select drone (father) populations from a DCA
droneGroups <- pullDroneGroupsFromDCA(DCA, n = 20, nDrones = nFathersTruncPoisson)
```

Once we inspect these drone groups, we see there is a different number of drones in each group because we used a sampling function:

```
sapply(droneGroups, FUN = nInd)
#>  [1] 14 19 21 17 14 15 13 15 21 14 18 17 22 19  6 16 16 11  7 13
```

Now, we can cross our virgin queens to drone groups.

```
# A single virgin queen
virginQueen1 <- cross(virginQueen1, drones = droneGroups[[1]])
nFathers(virginQueen1)
#> [1] 14
```

```
# Multiple virgin queens
virginQueens1 <- cross(virginQueens1, drones = droneGroups[2:4])
nFathers(virginQueens1)
#> [1] 19 21 17
```

```
# A colony
colony1 <- cross(colony1, drones = droneGroups[[5]])
nFathers(colony1)
#> [1] 14
```

```r
# An apiary
apiary1 <- cross(x = apiary1, drones = droneGroups[6:10])
nFathers(apiary1)
#>  5  6  7  8  9
#> 15 13 15 21 14
```

Cross according to a cross plan

Another option is to provide a cross plan with IDs of the virgin queens/colonies and drones, and a single drone population with all the drones listed in the cross plan. You can create a cross plan with the function `createRandomCrossPlan()`. This function creates a cross plan by randomly sampling a desired number of drone IDs from a DCA and assigning them to either virgin queen ID or colony ID. When crossing a virgin queen from a colony, you have to provide the colony ID, since there could be multiple virgin queens within the colony. In that case, the random selection of one virgin queen occurs within the `cross()` function. To create a cross plan you therefore have to provide the IDs of either the virgin queens or the colonies you want to cross (but not both in the same cross plan!!!), the drone population, and the number of drones you want to cross to a particular virgin queen. This can again be a fixed number or a sampling function. We can create a separate cross plan for each mating or create one combined cross plan for multiple matings (but can not have virgin queen and colony IDs in the cross plan at the same time!!!). Here, we again mate a single virgin queen, a population of virgin queens, virgin queens from a colony, and from an apiary.

```r
# Create a combined cross for mating a single queen (virginQueen2) and a population
# of virgin queen (virginQueens2)
(crossPlanQueens <- createRandomCrossPlan(IDs = c(getId(virginQueen2),
                                                  getId(virginQueens2)),
                                          drones = DCA,
                                          nDrones = 15))
#> $`2`
#>  [1] "2999" "1887" "2077" "3412" "7870" "6978" "1319" "8926" "7919" "6548"
#> [11] "1314" "501"  "8216" "4047" "8349"
#>
#> $`7`
#>  [1] "6149" "7813" "6114" "6107" "6411" "4860" "2312" "1148" "1619" "2199"
#> [11] "1389" "8751" "6502" "4544" "6635"
#>
#> $`8`
#>  [1] "6781" "7659" "6643" "904"  "6115" "7979" "5942" "2799" "4271" "3211"
#> [11] "6213" "8411" "4180" "4424" "1964"
#>
#> $`9`
#>  [1] "1267" "6512" "3997" "8384" "5935" "1116" "1672" "6102" "8612" "7508"
#> [11] "120"  "2851" "1491" "184"  "7330"
```

We see that the created cross plan is a list with IDs corresponding to the virgin queen's IDs and the elements of each list being the IDs of the drones the virgin queen will mate with. Now we can cross these virgin queens by providing the `crossPlanQueens` to the `cross()` functions.

```r
# Cross a single virgin queen
virginQueen2 <- cross(virginQueen2, drones = DCA, crossPlan = crossPlanQueens)
nFathers(virginQueen2)
#> [1] 15
# Cross multiple virgin queens
virginQueens2 <- cross(virginQueens2, drones = DCA, crossPlan = crossPlanQueens)
nFathers(virginQueens2)
#> [1] 15 15 15
```

As already mentioned, we need to create a separate plan for crossing virgin queens already in colonies, since here we need to provide colony IDs.

```
(crossPlanColonies <- createRandomCrossPlan(IDs = c(getId(colony2), getId(apiary2)),
                                            drones = DCA,
                                            nDrones = nFathersPoisson))
#> $`2`
#>  [1] "2480" "1656" "6425" "7683" "5121" "8328" "7684" "6221" "3823" "3819"
#> [11] "8867" "4902" "1628" "6724"
#>
#> $`10`
#>  [1] "1383" "1751" "3527" "8496" "4940" "5258" "7481" "3486" "6196" "3635"
#> [11] "4876" "943"  "6288" "2710" "4703" "565"  "1774" "1536" "3863"
#>
#> $`11`
#>  [1] "3165" "6282" "3562" "8567" "2404" "846"  "5236" "5186" "8334" "7814"
#> [11] "5425" "1324" "3712" "4039" "3928"
#>
#> $`12`
#>  [1] "2431" "2156" "8432" "5851" "6267" "1690" "96"   "8653" "3413" "5175"
#> [11] "5024" "2647" "5587" "2702" "6270" "5187" "3181" "2704" "7658"
#>
#> $`13`
#> [1] "643"  "7907" "306"  "3237" "6674" "1158" "5856" "6353"
#>
#> $`14`
#>  [1] "7312" "6544" "6367" "835"  "1104" "2171" "6829" "3488" "8368" "7942"
#> [11] "6239" "8737" "4606" "4033" "1998" "5081"
```

We again see that the cross plan is a list, now with colony IDs and elements of the list being the ID of drones the virgin queen within each colony will mate with. Now, we can cross our "colonies" by providing the `crossPlanColonies` to the `cross()` function.

```
# Cross a single colony
colony2 <- cross(colony2, drones = DCA, crossPlan = crossPlanColonies)
nFathers(colony2)
#> [1] 14
# Cross an apiary
apiary2 <- cross(x = apiary2, drones = DCA, crossPlan = crossPlanColonies)
nFathers(apiary2)
#> 10 11 12 13 14
#> 19 15 19  8 16
```

Cross virgin queens at a mating station

Mating virgin queens at a mating station is no different than mating them at an open DCA - the difference is in the DCA itself. In the case of open mating, the DCA consists of drones from multiple queens, all of which are usually unknown. In the case of a mating station, the DCA consists of drones coming from a sister group of drone producing queens (DPQ), the queen of which is known. This allows us to track the pedigree on the paternal side.

To simulate this situation, we first create a mating station DCA using `createMatingStationDCA()` function, which takes a single "sire" colony (queen of the DPQs). From the "sire" colony, the function first produces a desired number of sister DPQs, and next produces a desired number of drones per DPQ. The produced drones represent the mating station's DCA.

```r
# Create a DCA at a mating station from colony1
(matingStationDCA <- createMatingStationDCA(colony1, nDPQs = 20, nDronePerDPQ = 1000))
#> An object of class "Pop"
#> Ploidy: 2
#> Individuals: 20000
#> Chromosomes: 1
#> Loci: 100
#> Traits: 0
```

We see that the output of the function is a single population of 20,000 drones that represents the DCA. Once you have the DCA, you can cross virgin queens either by pulling out populations of drones or creating a mating plan as described above.

Here, we will mate a single colony (`colony3`) and a group of colonies (`apiary3`) at a mating station according to a cross plan.

```r
# Mate only an apiary
crossPlanMatingStation <- createRandomCrossPlan(IDs = c(getId(colony3),
                                                        getId(apiary3)),
                                                drones = matingStationDCA,
                                                nDrones = nFathersTruncPoisson)
# Cross a colony
colony3 <- cross(colony3, crossPlan = crossPlanMatingStation, drones = matingStationDCA)
nFathers(colony3)
#> [1] 12
# Cross an apiary
apiary3 <- cross(apiary3, crossPlan = crossPlanMatingStation, drones = matingStationDCA)
nFathers(apiary3)
#> 15 16 17 18 19
#> 11 13 13 15 20
```

Cross virgin queens with different methods

It could happen that you have some virgin colonies in an apiary and you want to inseminate one of the virgin queens artificially with a single drone, take three of them to a mating station, and mate the rest of them openly at a local DCA. Since the cross plan is a named list, you can concatenate multiple cross plans into one. Let's mate the multicolony `apiary4` in such a manner.

```r
# Create a single drone for single drone insemination
singleDrone = createDrones(colony2, nInd = 1)
# Create a cross plan for crossing some of the colonies in an open DCA,
# some with single drone, and some on a mating station
crossPlanApiary4 <- c(
  createRandomCrossPlan(IDs = getId(apiary4)[1],
                        drones = singleDrone,
                        nDrones = 1),
  createRandomCrossPlan(IDs = getId(apiary4)[2:6],
                        drones = DCA,
                        nDrones = nFathersTruncPoisson),
  createRandomCrossPlan(IDs = getId(apiary4)[7:10],
                        drones = matingStationDCA,
                        nDrones = nFathersTruncPoisson)
  )

apiary4 <- cross(apiary4,
```

```
                 crossPlan = crossPlanApiary4,
                 drones = c(singleDrone, DCA, matingStationDCA))
nFathers(apiary4)
#> 20 21 22 23 24 25 26 27 28 29
#>  1 18 16 15  9 14 13 17 16 17
```
