# Supplementary material for: SIMplyBee: an R package to simulate honeybee populations and breeding programs
Source: Genet Sel Evol. 2023 May 9;55:31. doi: 10.1186/s12711-023-00798-y (PMC10169377; doi:10.1186/s12711-023-00798-y)
Supplement: Supplementary file 5 — Additional file 5. Genomics vignette. This vignette demonstrates how to obtain genomic informationof simulated honeybees. It also demonstrates, how to compute honeybeegenomic relationship matrices in SIMplyBee [62–67]. This vignette can also be found on https://cran.r-project.org/package=SIMplyBee and http://www.SIMplyBee.info. [file 12711_2023_798_MOESM5_ESM.pdf]

Additional file 5 - Genomics vignette

2023-03-24

Introduction

This vignette demonstrates how SIMplyBee manages and manipulates the honey bee's genomic information. Specifically, it describes:

- how to obtain the genomic information,
- how to pool genotypes, and
- how to compute genomic relationship matrices.

Let's first create a colony.

```
library(package = "SIMplyBee")
#> Loading required package: AlphaSimR
#> Loading required package: R6
#>
#> Attaching package: 'SIMplyBee'
#> The following object is masked from 'package:base':
#>
#>     split
founderGenomes <- quickHaplo(nInd = 2, nChr = 3, segSites = 100)
SP <- SimParamBee$new(founderGenomes)
SP$setTrackRec(TRUE) # request recombination tracking

baseQueens <- createVirginQueens(founderGenomes)
baseDrones <- createDrones(x = baseQueens[1], nInd = 15)

colony <- createColony(x = baseQueens[2])
colony <- cross(colony, drones = baseDrones)
colony <- buildUp(colony)
```

Obtaining genomic information

Honeybees have a haplo-diploid inheritance system where queens and workers are diploid and drones are haploid. In SIMplyBee, we simulate drones as doubled-haploids, that is, as fully homozygous diploid individuals. This means that they have two identical sets of chromosomes. When they produce sperm, their gametes all have the same one set of chromosomes. Despite them being diploid, we generally return a haploid set of chromosomes from drones, unless specifically requested that you want the doubled-haploid genotype.

Following AlphaSimR, SIMplybee has a group of genome retrieval functions `get*Haplo/Geno()` which extract haplotypes and genotypes for all segregating sites (`SegSites`), quantitative trait loci (`QTL`), markers (`SNP`), and the identical by descent (`IBD`) haplotypes. Here, site, locus and marker are all synonyms for a position in the genome. These functions leverage AlphaSimR functionality, but work with SIMplyBee's `Colony` or `MultiColony` objects and in addition take the `caste` argument to extract information for a specific caste. Another argument you can use with this function is `collapse = TRUE/FALSE`. If `collapse = TRUE` then all of the information is collapsed together and a single matrix is returned, if `collapse = FALSE` we return a list by caste or by colony.

We recommend that you study the index of available `get*()` functions in SIMplyBee and read this vignette for a short demonstration.

```
help(SIMplyBee)
```

To show all this functionality, let's get haplotypes and genotypes across the segregating sites for the different castes using `getSegSitesGeno()` or `getSegSitesHaplo()`. The first row of the output shows marker identifications (chromosome_locus) and the first column shows haplotype identifications (individual_haplotype). The alleles are represented with a sequence of 0's and 1's. Let's first obtain the information at the segregating sites for the queen (we limit the output to the first 10 sites):

```
getSegSiteHaplo(colony, caste = "queen")[, 1:10]
#>     1_1 1_2 1_3 1_4 1_5 1_6 1_7 1_8 1_9 1_10
#> 2_1   1   0   0   1   1   1   1   0   1    1
#> 2_2   0   0   1   0   0   0   1   1   0    0
```

```
getSegSiteGeno(colony, caste = "queen")[, 1:10]
#>  1_1  1_2  1_3  1_4  1_5  1_6  1_7  1_8  1_9 1_10
#>    1    0    1    1    1    1    2    1    1    1
```

Now for the fathers:

```
getSegSiteHaplo(colony, caste = "fathers")[, 1:10]
#>      1_1 1_2 1_3 1_4 1_5 1_6 1_7 1_8 1_9 1_10
#> 9_1    0   1   0   0   1   1   1   1   0    0
#> 10_1   1   0   0   1   0   1   1   1   0    1
#> 3_1    1   0   0   1   0   1   1   1   0    1
#> 4_1    0   1   0   0   1   1   1   1   0    1
#> 7_1    0   1   0   0   1   1   1   1   0    0
#> 11_1   0   1   0   0   1   1   1   1   0    0
#> 14_1   0   1   0   0   1   1   1   1   0    0
#> 15_1   1   0   0   1   0   1   1   1   0    1
#> 17_1   0   1   0   0   1   1   1   1   0    0
#> 6_1    0   1   0   1   0   1   1   1   0    1
#> 12_1   0   1   0   0   1   1   1   1   0    0
#> 13_1   1   0   0   1   0   1   1   1   0    1
#> 5_1    1   0   0   1   0   1   1   1   0    1
#> 8_1    0   1   0   0   1   1   1   1   0    0
#> 16_1   1   0   0   1   0   1   1   1   0    0
```

```
getSegSiteGeno(colony, caste = "fathers")[, 1:10]
#>    1_1 1_2 1_3 1_4 1_5 1_6 1_7 1_8 1_9 1_10
#> 3    1   0   0   1   0   1   1   1   0    1
#> 10   1   0   0   1   0   1   1   1   0    1
#> 4    0   1   0   0   1   1   1   1   0    1
#> 12   0   1   0   0   1   1   1   1   0    0
#> 8    0   1   0   0   1   1   1   1   0    0
#> 5    1   0   0   1   0   1   1   1   0    1
#> 16   1   0   0   1   0   1   1   1   0    0
#> 9    0   1   0   0   1   1   1   1   0    0
#> 15   1   0   0   1   0   1   1   1   0    1
#> 13   1   0   0   1   0   1   1   1   0    1
#> 6    0   1   0   1   0   1   1   1   0    1
#> 17   0   1   0   0   1   1   1   1   0    0
#> 7    0   1   0   0   1   1   1   1   0    0
#> 11   0   1   0   0   1   1   1   1   0    0
#> 14   0   1   0   0   1   1   1   1   0    0
```

```
```

Since father are drones, and these are haploid, we get one row per father. We can retrieve the doublet-haploid (diploid implementation) state, if this is desired (showing just one father to show this clearly):

```
getSegSiteHaplo(colony, caste = "fathers",
                nInd = 1, dronesHaploid = FALSE)[, 1:10]
#>     1_1 1_2 1_3 1_4 1_5 1_6 1_7 1_8 1_9 1_10
#> 6_1   0   1   0   1   0   1   1   1   0    1
#> 6_2   0   1   0   1   0   1   1   1   0    1
```

```
getSegSiteGeno(colony, caste = "fathers",
               nInd = 1, dronesHaploid = FALSE)[, 1:10, drop = FALSE]
#>   1_1 1_2 1_3 1_4 1_5 1_6 1_7 1_8 1_9 1_10
#> 7   0   2   0   0   2   2   2   2   0    0
```

Now two workers:

```
getSegSiteHaplo(colony, caste = "workers", nInd = 2)[, 1:10]
#>      1_1 1_2 1_3 1_4 1_5 1_6 1_7 1_8 1_9 1_10
#> 67_1   1   0   0   1   1   1   1   0   1    1
#> 67_2   0   1   0   0   1   1   1   1   0    0
#> 89_1   1   0   0   1   1   1   1   0   1    1
#> 89_2   0   1   0   0   1   1   1   1   0    0
```

```
getSegSiteGeno(colony, caste = "workers", nInd = 2)[, 1:10]
#>     1_1 1_2 1_3 1_4 1_5 1_6 1_7 1_8 1_9 1_10
#> 104   0   1   1   0   1   1   2   2   0    1
#> 75    2   0   0   2   1   2   2   1   1    2
```

And finally four drones:

```
getSegSiteHaplo(colony, caste = "drones", nInd = 4)[, 1:10]
#>       1_1 1_2 1_3 1_4 1_5 1_6 1_7 1_8 1_9 1_10
#> 173_1   1   0   0   1   1   1   1   1   0    0
#> 177_1   1   0   0   1   1   1   1   0   1    1
#> 185_1   0   0   1   0   0   0   1   1   0    0
#> 130_1   1   0   0   1   1   1   1   0   1    1
```

```
getSegSiteGeno(colony, caste = "drones", nInd = 4)[, 1:10]
#>     1_1 1_2 1_3 1_4 1_5 1_6 1_7 1_8 1_9 1_10
#> 191   1   0   0   1   1   1   1   0   1    1
#> 148   0   0   1   0   0   0   1   1   0    0
#> 171   0   0   1   0   0   0   1   1   0    0
#> 210   0   0   1   0   0   0   1   1   0    0
```

You can also use `caste = "all"` to get the haplotypes and phenotypes from every individual in the colony. If the argument `collapse` is set to `FALSE`, then the function returns a list with haplotypes for each caste. Let's explore the structure of the output:

```
str(getSegSiteHaplo(colony, caste = "all", collapse = FALSE))
#> List of 5
#>  $ queen       : int [1:2, 1:300] 1 0 0 0 0 1 1 0 1 0 ...
#>   ..- attr(*, "dimnames")=List of 2
#>   .. ..$ : chr [1:2] "2_1" "2_2"
#>   .. ..$ : chr [1:300] "1_1" "1_2" "1_3" "1_4" ...
#>  $ fathers     : int [1:15, 1:300] 0 0 0 0 1 1 0 1 0 0 ...
#>   ..- attr(*, "dimnames")=List of 2
```

```
#>   .. ..$ : chr [1:15] "4_1" "8_1" "17_1" "7_1" ...
#>   .. ..$ : chr [1:300] "1_1" "1_2" "1_3" "1_4" ...
#>  $ workers     : int [1:200, 1:300] 0 0 0 0 0 1 1 1 0 0 ...
#>   ..- attr(*, "dimnames")=List of 2
#>   .. ..$ : chr [1:200] "57_1" "57_2" "91_1" "91_2" ...
#>   .. ..$ : chr [1:300] "1_1" "1_2" "1_3" "1_4" ...
#>  $ drones      : int [1:100, 1:300] 1 1 1 0 0 0 0 1 1 0 ...
#>   ..- attr(*, "dimnames")=List of 2
#>   .. ..$ : chr [1:100] "137_1" "216_1" "145_1" "179_1" ...
#>   .. ..$ : chr [1:300] "1_1" "1_2" "1_3" "1_4" ...
#>  $ virginQueens: NULL
```

If the argument `collapse` is set to `TRUE`, the function returns a single matrix with haplotypes of all the individuals. The same behaviour is implemented for all the functions that extract genomic information

```
str(getSegSiteHaplo(colony, caste = "all", collapse = TRUE))
#>  int [1:317, 1:300] 1 0 0 0 0 1 1 1 0 1 ...
#>  - attr(*, "dimnames")=List of 2
#>   ..$ : chr [1:317] "2_1" "2_2" "4_1" "8_1" ...
#>   ..$ : chr [1:300] "1_1" "1_2" "1_3" "1_4" ...
```

```
getSegSiteHaplo(colony, caste = "all", collapse = TRUE)[1:10, 1:10]
#>      1_1 1_2 1_3 1_4 1_5 1_6 1_7 1_8 1_9 1_10
#> 2_1    1   0   0   1   1   1   1   0   1    1
#> 2_2    0   0   1   0   0   0   1   1   0    0
#> 5_1    1   0   0   1   0   1   1   1   0    1
#> 15_1   1   0   0   1   0   1   1   1   0    1
#> 8_1    0   1   0   0   1   1   1   1   0    0
#> 11_1   0   1   0   0   1   1   1   1   0    0
#> 7_1    0   1   0   0   1   1   1   1   0    0
#> 16_1   1   0   0   1   0   1   1   1   0    0
#> 3_1    1   0   0   1   0   1   1   1   0    1
#> 4_1    0   1   0   0   1   1   1   1   0    1
```

```
getSegSiteGeno(colony, caste = "all", collapse = TRUE)[1:10, 1:10]
#>    1_1 1_2 1_3 1_4 1_5 1_6 1_7 1_8 1_9 1_10
#> 2    1   0   1   1   1   1   2   1   1    1
#> 4    0   1   0   0   1   1   1   1   0    1
#> 8    0   1   0   0   1   1   1   1   0    0
#> 3    1   0   0   1   0   1   1   1   0    1
#> 6    0   1   0   1   0   1   1   1   0    1
#> 15   1   0   0   1   0   1   1   1   0    1
#> 14   0   1   0   0   1   1   1   1   0    0
#> 17   0   1   0   0   1   1   1   1   0    0
#> 5    1   0   0   1   0   1   1   1   0    1
#> 13   1   0   0   1   0   1   1   1   0    1
```

SIMplyBee also has shortcuts for these haplotype and genotype functions to make life a bit easier for the user:

- `getQueenSegSitesHaplo()`
- `getQueenSegSitesGeno()`
- `getFathersSegSitesHaplo()`
- `getFathersSegSitesGeno()`

- `getWorkersSegSitesHaplo()`
- `getWorkersSegSitesGeno()`
- `getDronesSegSitesHaplo()`
- `getDronesSegSitesGeno()`
- `getVirginQueensSegSitesHaplo()`
- `getVriginQueensSegSitesGeno()`

Similar aliases exist also for extracting information about quantitative trait loci (`QTL`), markers (`SNP`), and the identical by descent (`IBD`) haplotypes.

Pooling genotypic information

Unfortunately, in real life it's challenging to get the genotype of every individual honeybee and so SIMplyBee provides the function `getPooledGeno()` to imitate real life data. `getPooledGeno()` returns a pooled genotype from individual genotypes to mimic the genotyping of a pool of colony members. A comparison of pooled and individual genotypes also allows the user to compare the two and see the impact of pooled samples on results.

Firstly let's obtain the genotypes of the workers and of the queen so that they're easier to work with:

```
genoQ <- getSegSiteGeno(colony, caste = "queen")
genoW <- getSegSiteGeno(colony, caste = "workers")
```

The function `getPooledGeno()` required also the sex of individuals whose genotype are getting pooled (`F` for females and `M` for males).

```
sexW <- getCasteSex(colony, caste = "workers")
```

You have two options when choosing what kind of pooled genotypes you would like, using the `type =` argument. You can use `type = "mean"` for the average genotypes and `type = "count"` for the counts of reference and alternative alleles.

```
getPooledGeno(x = genoW, type = "count", sex = sexW)[, 1:10]
#>   1_1 1_2 1_3 1_4 1_5 1_6 1_7 1_8 1_9 1_10
#> 0 109 139 151 104  96  47   0  52 145   96
#> 1  91  61  49  96 104 153 200 148  55  104
```

```
(poolW <- getPooledGeno(x = genoW, type = "mean", sex = sexW))[, 1:10]
#>  1_1  1_2  1_3  1_4  1_5  1_6  1_7  1_8  1_9 1_10
#> 0.91 0.61 0.49 0.96 1.04 1.53 2.00 1.48 0.55 1.04
```

Now lets plot and compare the pooled workers to the queen's genotype (note the use of jitter for queen's genotype on the x-axis so we can spread out the dots in the plot!).

```
plot(y = poolW, x = jitter(genoQ), ylim = c(0, 2), xlim = c(0, 2),
     ylab = "Average allele dosage in workers",
     xlab = "Allele dosage in the queen" )
```

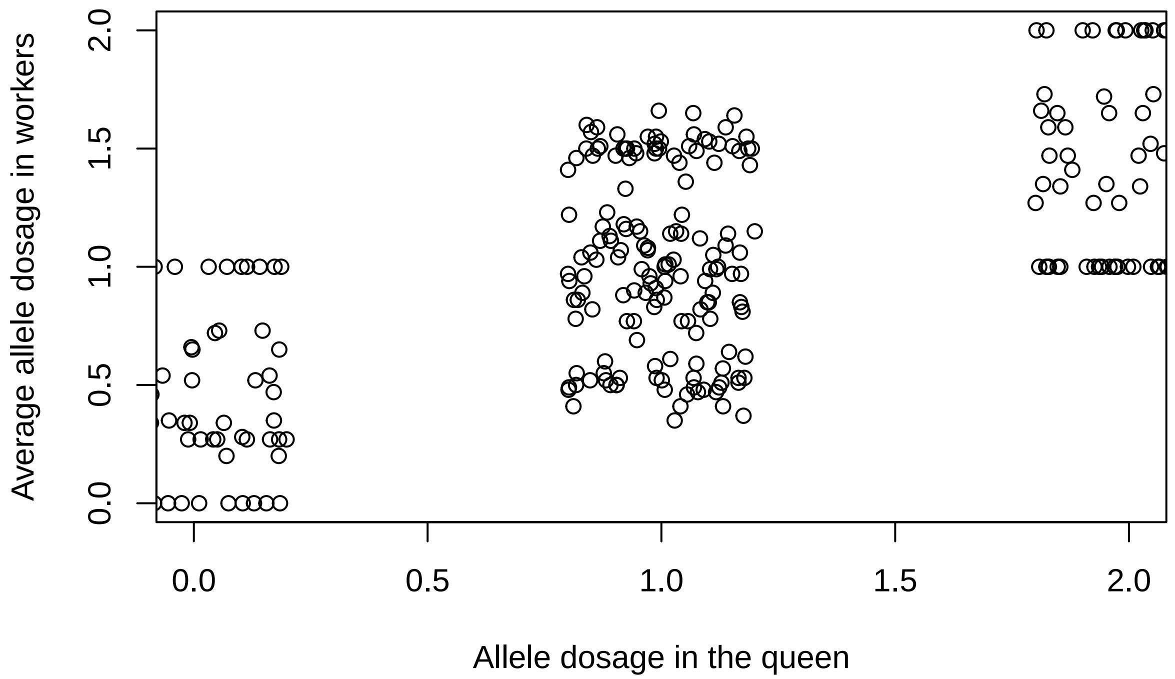

Computing Genomic Relationship Matrices

This section introduces the calculations of IBD and IBS genomic relationship matrices, so let's have a quick reminder of what these mean. Identity-by-state (IBS) is a term used when two alleles, two segments or sequences of the genome are identical. Identity-by-descent (IBD) is when a segment of matching (IBS) DNA shared by two or more individuals has been inherited from a common ancestor.

Using IBD and IBS can allow a user to look into the relationships based on the genomic data. We'll demonstrate this by calculating some Genomic Relationship Matrices (GRM) using SIMplyBee's `calcBeeGRMIbs()` and `calcBeeGRMIbd()`.

Let's look at the `calcBeeGRMIbs()` first. This function returns a Genomic Relatedness Matrix (GRM) for honeybees from IBS genomic data (bi-allelic SNP represented as allele dosages) following the method for the sex X chromosome [62].

To see this, let's obtain the genotypes and sex information of all individuals in the colony.

```
geno <- getSegSiteGeno(colony, collapse = TRUE)
sex <- getCasteSex(x = colony, collapse = TRUE)
```

Now let's calculate the IBS GRM, we will use the genotypes to calculate this:

```
GRM <- calcBeeGRMIbs(x = geno, sex = sex)
```

This produces a matrix that we can plot and summarise - its useful to summarise diagonal and off-diagonal values separately.

```
library("Matrix")
image(as(GRM, "Matrix"))
```

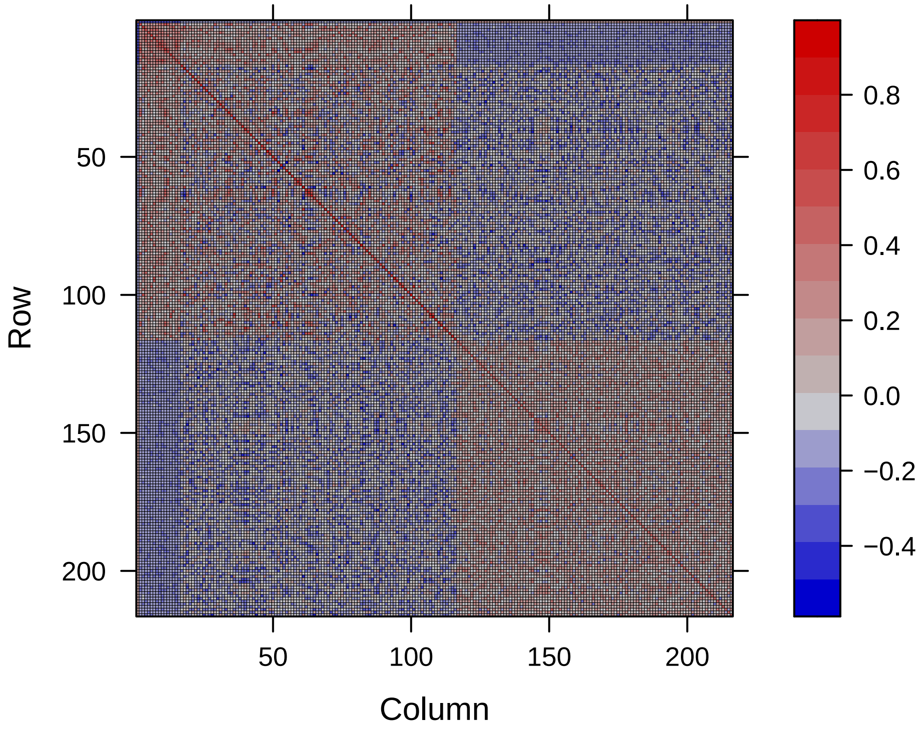

Dimensions: 216 x 216

```
x <- diag(GRM)
hist(x)
```

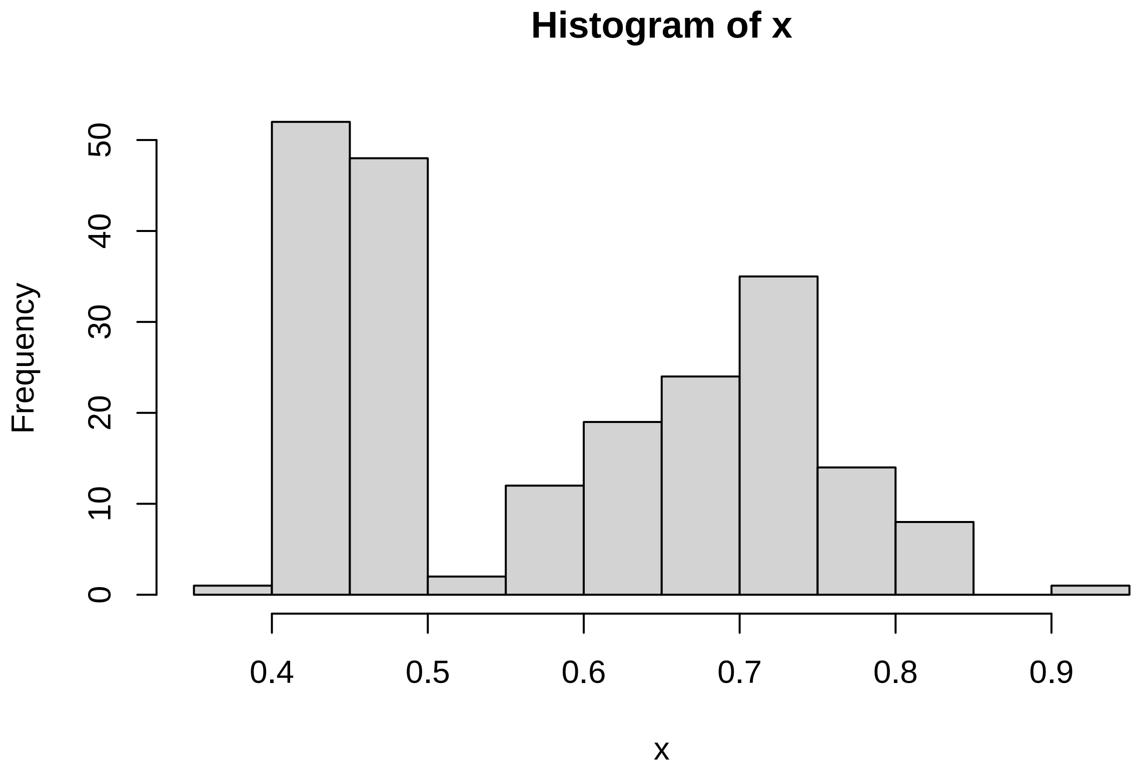


```
summary(x)
#>    Min. 1st Qu.  Median    Mean 3rd Qu.    Max.
#>  0.3829  0.4506  0.5791  0.5788  0.7025  0.9008
```

```
x <- GRM[lower.tri(x = GRM, diag = FALSE)]
hist(x)
```

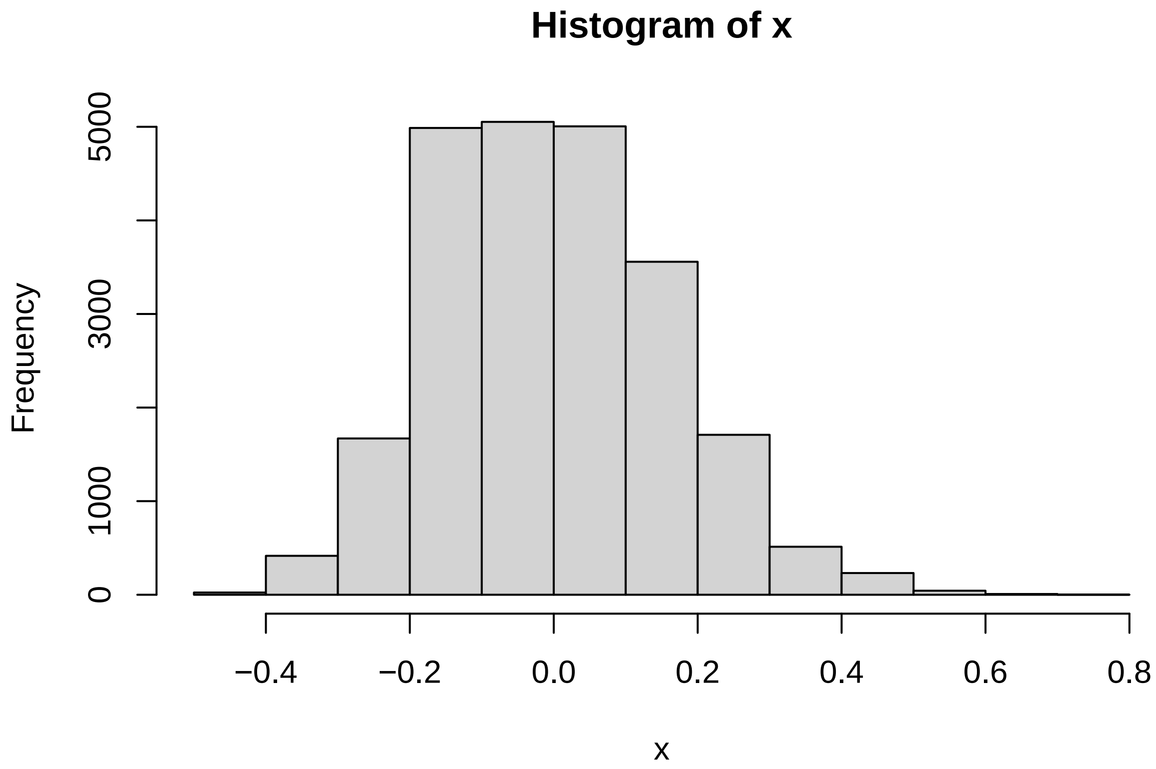


```
summary(x)
#>      Min.   1st Qu.    Median      Mean   3rd Qu.      Max.
#> -0.490409 -0.127333 -0.010990 -0.002692  0.105466  0.707445
```

We can also inspect GRM elements between specific caste members:

```
ids <- getCasteId(colony)
idQueen <- ids$queen
idFathers <- ids$fathers
idWorkers <- ids$workers
idDrones <- ids$drones
idVirginQueens <- ids$virginQueens
mw <- "mw"
md <- "md"
```

```
r <- range(GRM)
hist(GRM[idQueen, idFathers], xlim = r)
```

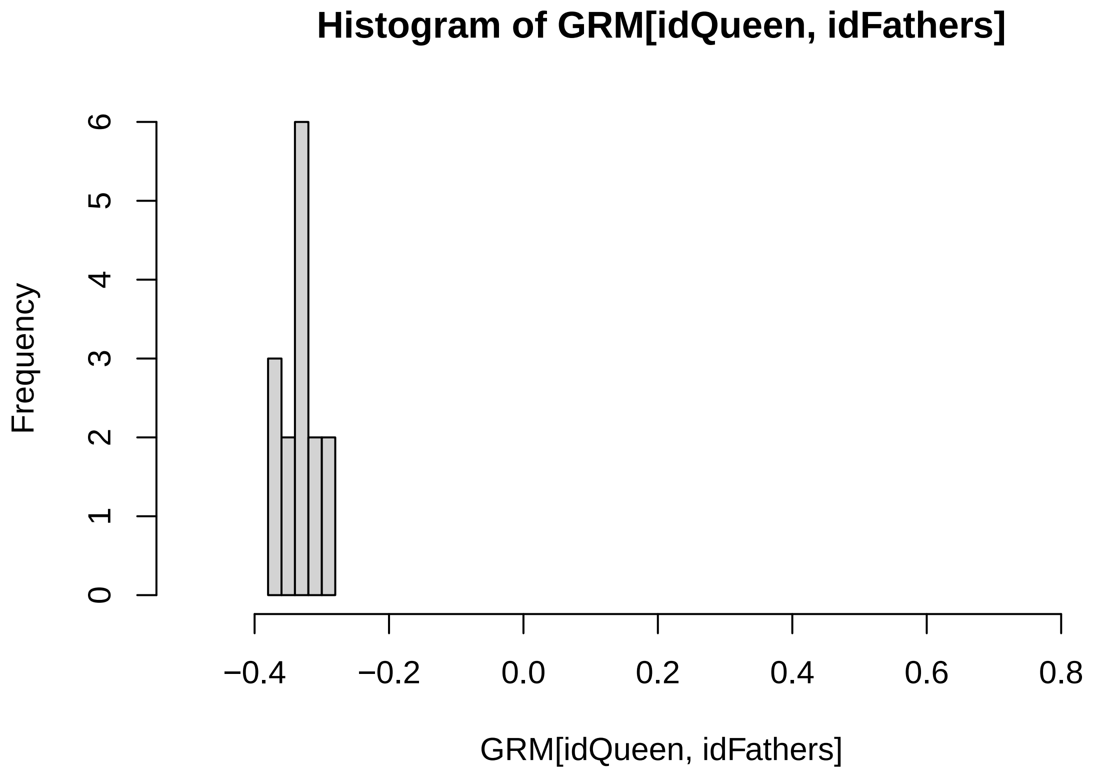


```
hist(GRM[idQueen, idWorkers], xlim = r)
```

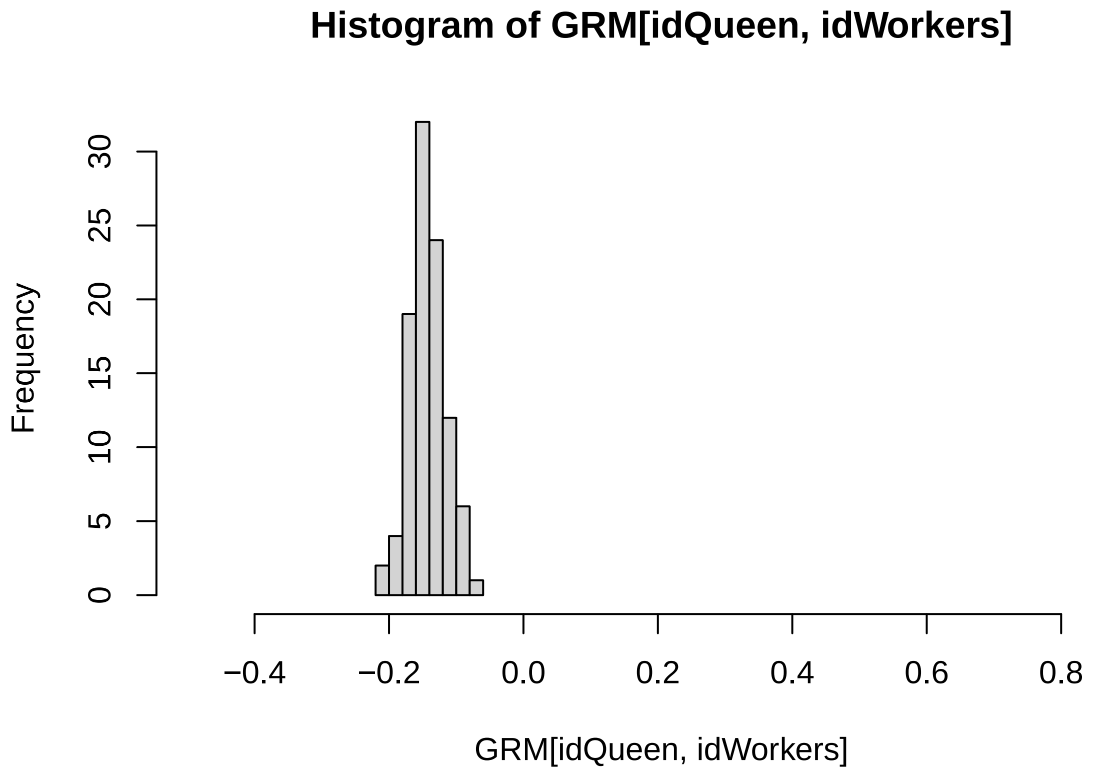


```
hist(GRM[idQueen, idDrones], xlim = r)
```

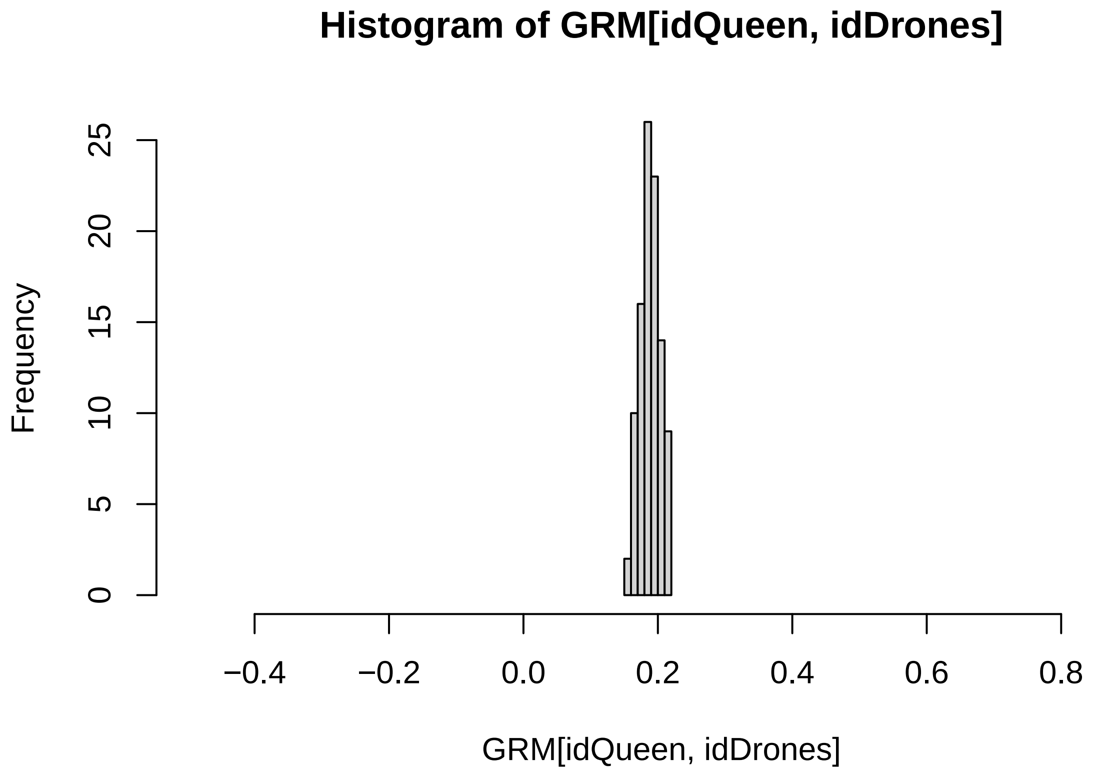

`calcBeeGRMIbs()` uses the `calcBeeAlleleFreq()` function to calculate allele frequencies for centering the honeybee genotypes. You can also use it in some other cases:

```
hist(alleleFreq <- calcBeeAlleleFreq(x = geno, sex = sex))
```

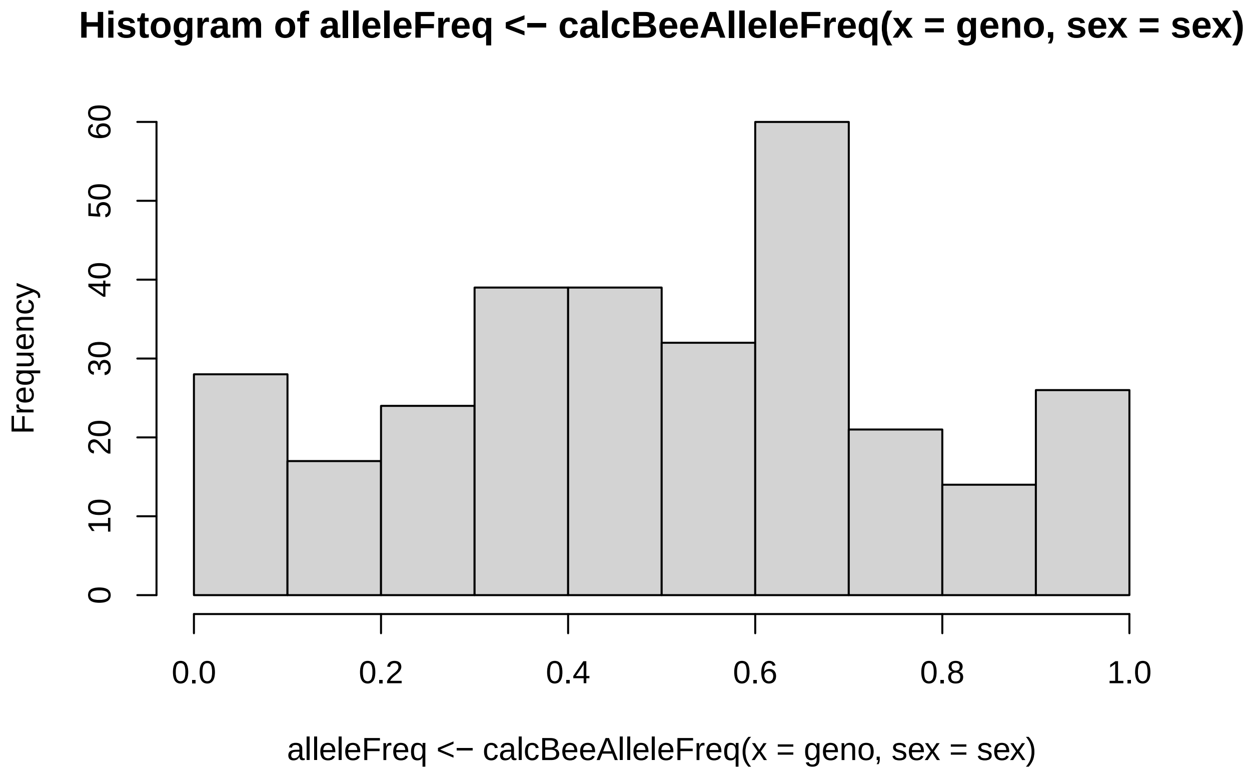

Now lets look at `calcBeeGRMIbd()`. This function creates Genomic Relatedness Matrix (GRM) for honeybees based on Identical-By-Descent (IBD) information. It returns a list with a matrix of gametic relatedness coefficients (between genomes) and a matrix of individual relatedness coefficients (between individuals). Please refer to [63-67] for the background on this function.

Now obtain the IBD haplotypes and compute IBD GRM.

```
haploQ <- getQueenIbdHaplo(colony)
haploF <- getFathersIbdHaplo(colony)
haploW <- getWorkersIbdHaplo(colony)
haploD <- getDronesIbdHaplo(colony)
haploV <- getVirginQueensIbdHaplo(colony)

haplo <- rbind(haploQ, haploF, haploW, haploD, haploV)
```

```
GRMs <- calcBeeGRMIbd(x = haplo)
```

Let's view this matrix:

```
image(as(GRMs$genome, "Matrix"))
```

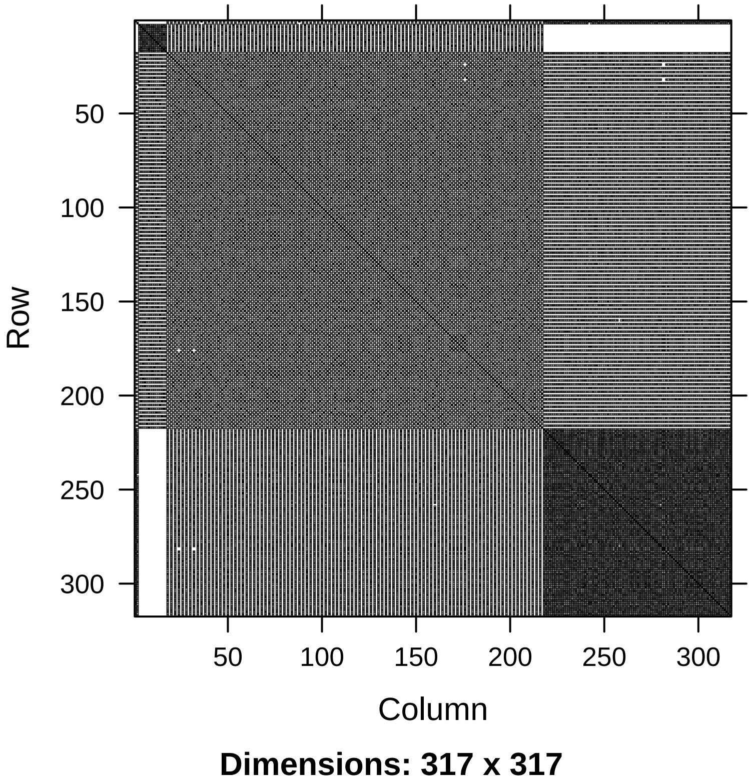


```
image(as(GRMs$indiv, "Matrix"))
```

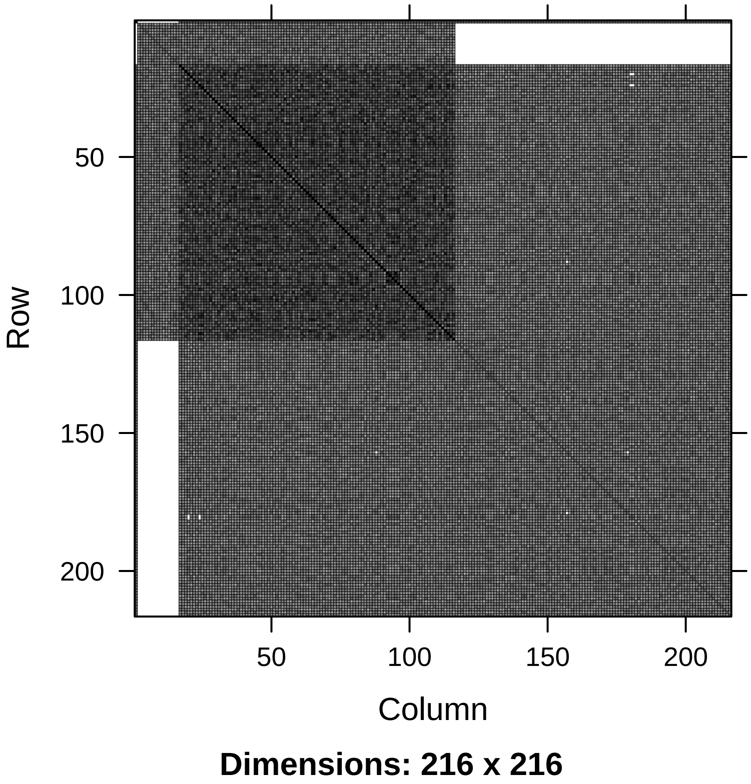

Now we can look at the diagonal of the obtained matrices that represent 1 for a genomes and 1 + inbreeding coefficient individuals.

```
i <- diag(GRMs$genome)
summary(x)
#>      Min.   1st Qu.    Median      Mean   3rd Qu.      Max.
#> -0.490409 -0.127333 -0.010990 -0.002692  0.105466  0.707445

i <- diag(GRMs$indiv)
summary(i)
#>    Min. 1st Qu.  Median    Mean 3rd Qu.    Max.
#>  0.5000  0.5000  0.5000  0.7338  1.0000  1.0000
```

And now the non-diagonals that represent the coefficients of relationship between genomes or between individuals.

```
x <- GRMs$genome[lower.tri(x = GRMs$genome, diag = FALSE)]
hist(x)
```

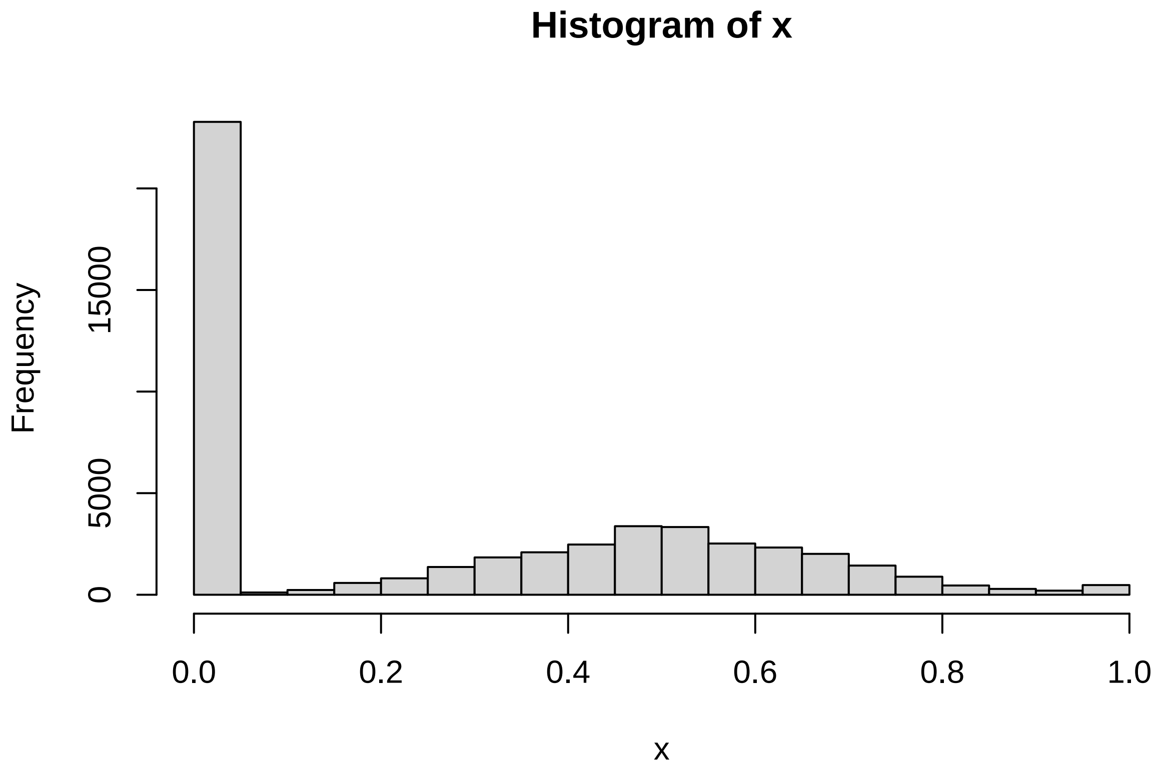


```
summary(x)
#>    Min. 1st Qu.  Median    Mean 3rd Qu.    Max.
#>  0.0000  0.0000  0.2533  0.2755  0.5233  1.0000

i <- GRMs$indiv[lower.tri(x = GRMs$indiv, diag = FALSE)]
hist(i)
```

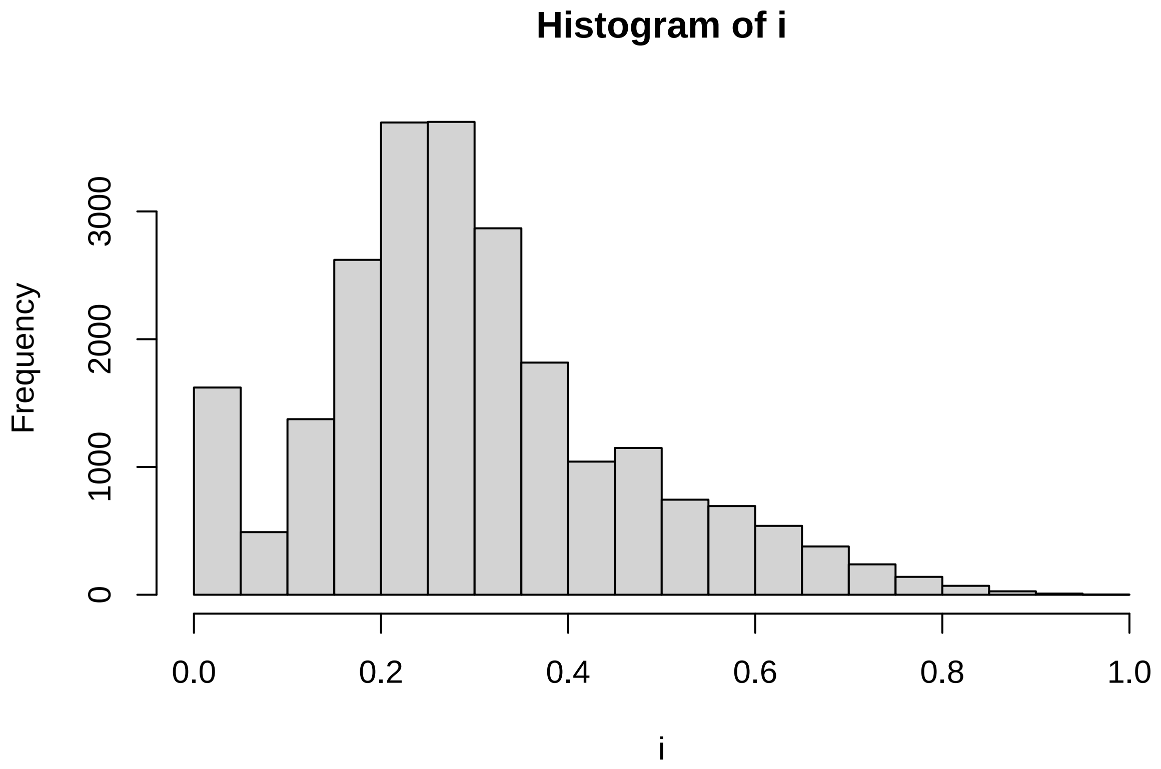


```
summary(i)
#>    Min. 1st Qu.  Median    Mean 3rd Qu.    Max.
#>  0.0000  0.1950  0.2733  0.2972  0.3767  0.9917
```

Let's now compare compare relationships between caste members within a colony.

```
# Obtains caste member IDs
qI <- getQueen(colony)@id
fI <- sort(getFathers(colony)@id)
wI <- sort(getWorkers(colony)@id)
dI <- sort(getDrones(colony)@id)
r <- range(GRMs$indiv)
```

Compare queen and fathers:

```
hist(GRMs$indiv[fI, qI], xlim = r)
```

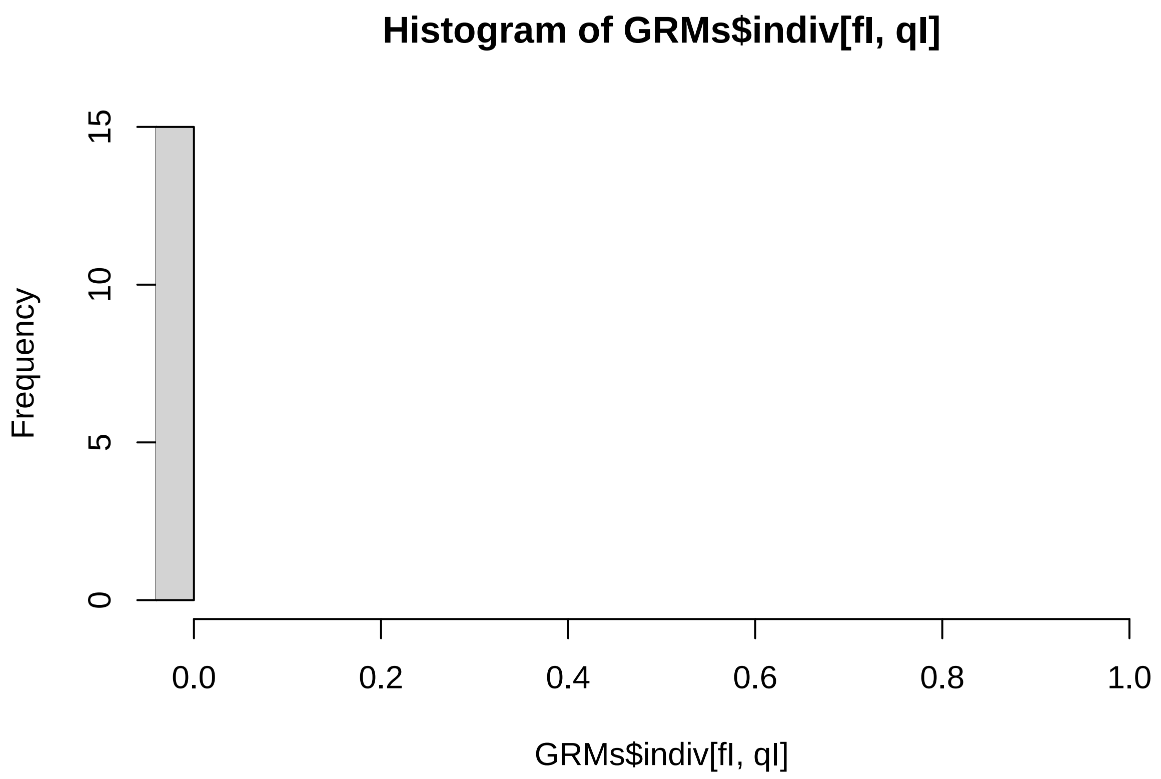

Queen and workers:

```
hist(GRMs$indiv[wI, qI], xlim = r)
```

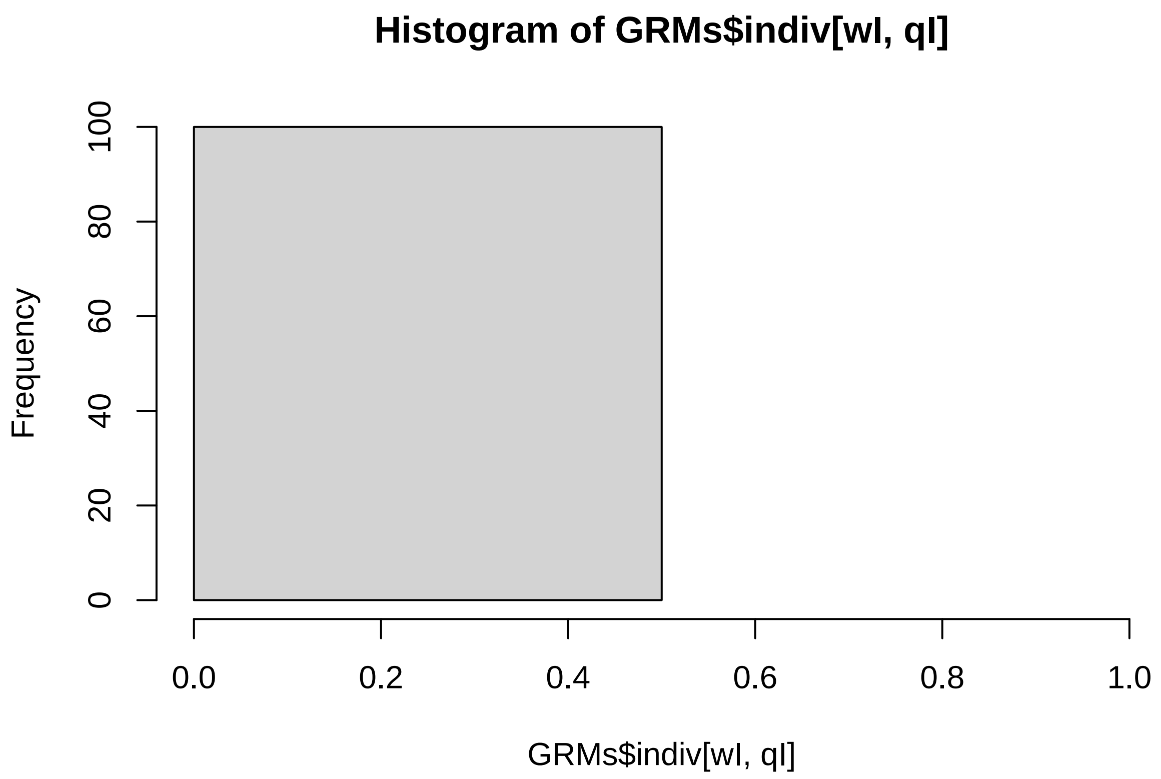

Queen and drones:

```
hist(GRMs$indiv[dI, qI], xlim = r)
```

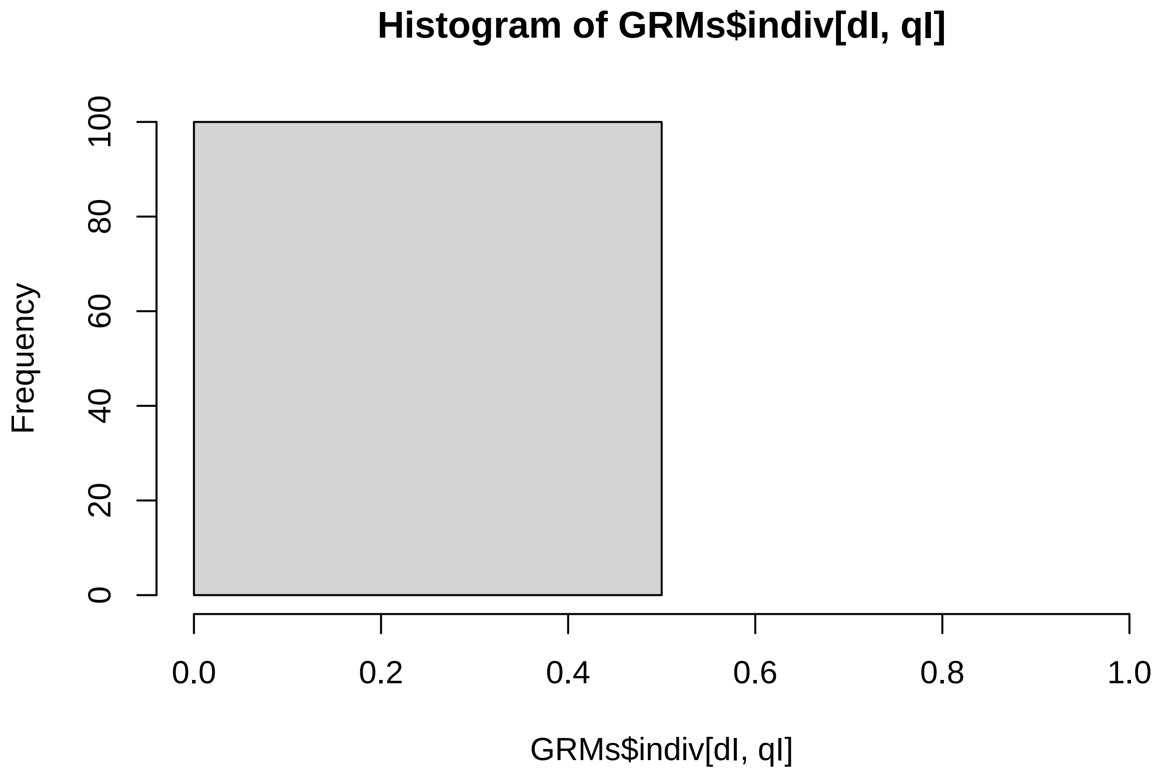
Histogram of GRMs$indiv[dI, qI]
Frequency
0
20
40
60
80
100
0.0
0.2
0.4
0.6
0.8
1.0
GRMs$indiv[dI, qI]
