# Supplementary material for: SIMplyBee: an R package to simulate honeybee populations and breeding programs
Source: Genet Sel Evol. 2023 May 9;55:31. doi: 10.1186/s12711-023-00798-y (PMC10169377; doi:10.1186/s12711-023-00798-y)

Additional file 6 - Quantitative genetics vignette

2023-03-21

Introduction

This vignette describes and demonstrates how SIMplyBee implements quantitative genetics principles for honeybees. Specifically, it describes three different examples where we simulate:

1. Honey yield - a single colony trait,
2. Honey yield and Calmness - two colony traits, and
3. Colony strength and Honey yield - two colony traits where one trait impacts the other one via the number of workers.

We start by loading SIMplyBee and quickly simulating genomes for some founder honeybees. Specifically, we will simulate genomes for 20 individuals with 16 chromosomes and 1000 segregating sites per chromosome.

```
library(package = "SIMplyBee")
#> Loading required package: AlphaSimR
#> Loading required package: R6
#>
#> Attaching package: 'SIMplyBee'
#> The following object is masked from 'package:base':
#>
#>     split
library(package = "ggplot2")
founderGenomes <- quickHaplo(nInd = 20, nChr = 16, segSites = 1000)
```

Honey yield

This section shows how to simulate one colony trait, honey yield, that is influenced by the queen and workers as well as the environment. We will achieve this by:

a. setting base population quantitative genetic parameters,
b. inspecting individual values in the base population,
c. inspecting individual values in a colony,
d. calculating colony value,
e. calculating multi-colony values, and
f. selecting on colony values.

Base population quantitative genetic parameters

AlphaSimR, and hence SIMplyBee, simulates each individual with its corresponding genome, and quantitative genetic and phenotypic values. To enable this simulation, we must set base population quantitative genetic parameters for the traits of interest in the global simulation parameters via `SimParamBee`. We must set:

1) the number of traits,
2) the number of quantitative trait loci (QTL) that affect the traits,
3) the distribution of QTL effects,

4) trait means, and
5) trait genetic and environmental variances - if we simulate multiple traits, we must also specify genetic and environmental covariances between the traits.

In honeybees, the majority of traits are influenced by the queen and workers. There are many biological mechanisms for these queen and workers effects. Depending on which caste is the main driver of the trait (the queen or workers), we also talk about direct and indirect effects. For example, for honey yield, workers directly affect honey yield by foraging, while the queen indirectly affects honey yield by stimulating workers via pheromone production. The queen and workers effects for a trait can be genetically and environmentally independent or correlated (usually negatively).

Here, we will simulate two traits to represent the queen and workers effects on honey yield. From this point onward we will use the terms the queen effect and queen trait interchangeably. The same applies to workers effect and workers trait. These two effects (=traits) will give rise to honey yield trait. We will assume that colony honey yield is approximately normally distributed with the mean of 20 kg and variance of 4 kg^2, which implies that most colonies will have honey yield between 14 kg and 26 kg (see `hist(rnorm(n = 1000, mean = 20, sd = sqrt(4)))`). Traits like honey yield have a complex polygenic genetic architecture, so we will assume that this trait is influenced by 100 QTL per chromosome (with 16 chromosomes, this gives us 1600 QTL in total).

We will first initiate global simulation parameters and set the mean of queen effects to 10 kg with genetic variance of 1 kg^2, while we will set the mean of workers effects to 10 kg with genetic variance of 1 kg^2. The mean and the variance for the worker effect are proportionally scaled by the expected number of workers in a colony. The mean and variance for the queen effect is assumed larger than for the workers effect, because there is one queen and many workers in colony and we assume that workers effects "accumulate". Deciding how to split the colony mean between queen and workers effects will depend on the individual to colony mapping function, which we will describe in the Colony value sub-section.

```
# Global simulation parameters
SP <- SimParamBee$new(founderGenomes)

nQtlPerChr <- 100

# Genetic parameters for queen and workers effects - each represented by a trait
mean <- c(10, 10 / SP$nWorkers)
varA <- c(1, 1 / SP$nWorkers)
```

We next set genetic correlation between the queen and workers effects to -0.5 to reflect the commonly observed antagonistic relationship between these effects. With all the quantitative genetic parameters defined, we now add two additive traits to global simulation parameters and name them `queenTrait` and `workerTrait`. These parameters drive the simulation of QTL effects. Read about all the other trait simulation options in AlphaSimR via: `vignette(topic = "traits", package="AlphaSimR")`.

```
corA <- matrix(data = c( 1.0, -0.5,
                        -0.5,  1.0), nrow = 2, byrow = TRUE)
SP$addTraitA(nQtlPerChr = nQtlPerChr, mean = mean, var = varA, corA = corA,
             name = c("queenTrait", "workersTrait"))
```

Finally, we set the environmental variance of the queen and workers effects to 3 kg^2 and we again scale the worker variance by the expected number of workers. Contrary to the negative genetic correlation, we here assume that environmental correlation between the queen and workers effects is slightly positive, 0.3. This is just an example! These parameters should be based on literature or simulation scenarios of interest.

```
varE <- c(3, 3 / SP$nWorkers)
corE <- matrix(data = c(1.0, 0.3,
                        0.3, 1.0), nrow = 2, byrow = TRUE)
SP$setVarE(varE = varE, corE = corE)
```

Individual values in the base population

Now we create a base population of virgin queens. Since we defined two traits, all honeybees in the simulation will have genetic and phenotypic values for both traits. The genetic values are stored in the `gv` slot of each `Pop` object, while phenotypic values are stored in the `pheno` slot.

```
#>      queenTrait workersTrait
#> [1,]  10.960784   0.18577622
#> [2,]   8.815073   0.12664503
#> [3,]  10.307191   0.16095838
#> [4,]  10.195715  -0.05554425
#> [5,]  10.452741   0.05887891
#> [6,]  11.510637   0.06106399
#>      queenTrait workersTrait
#> [1,]   6.302605   -0.1898603
#> [2,]   7.269197    0.2845908
#> [3,]   9.828218    0.2334159
#> [4,]  10.197979   -0.1462330
#> [5,]  15.354724    0.2593664
#> [6,]  10.209329    0.1372256
```

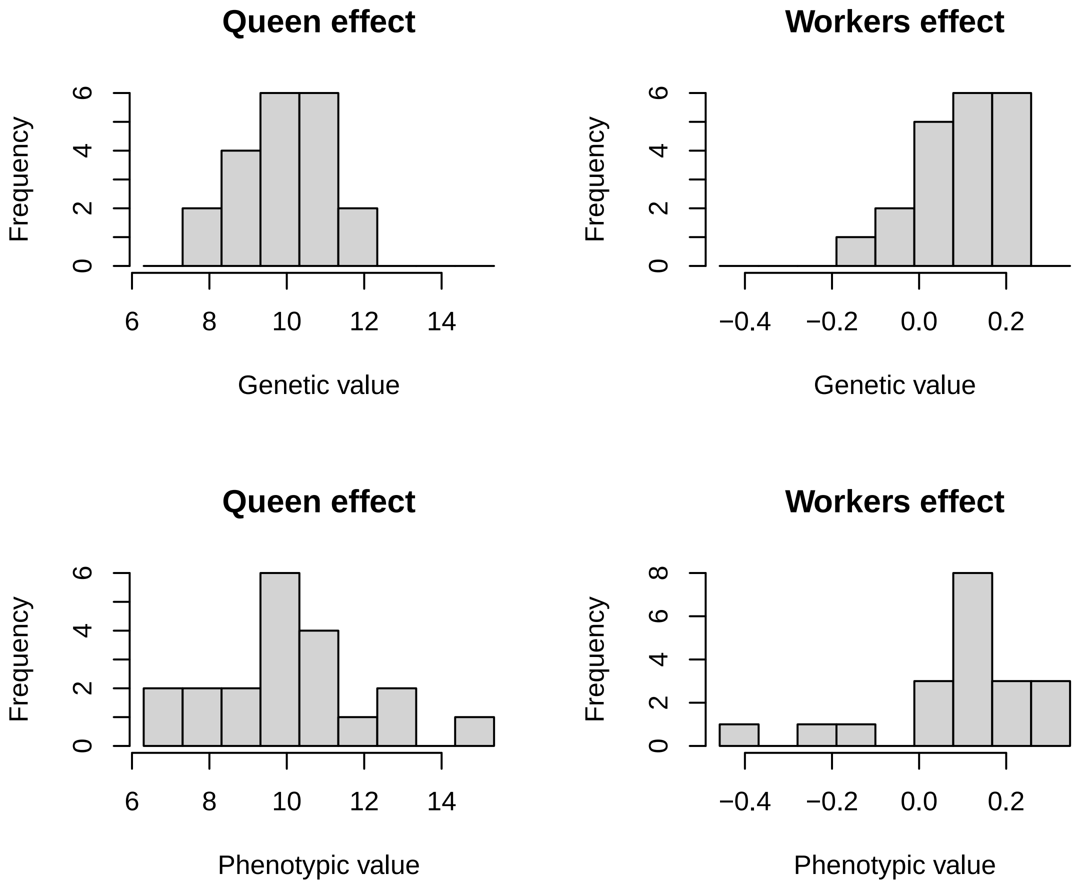

Note that these are virgin queens, yet we obtained queen and workers effect values for them! Is this wrong? No! Virgin queens carry DNA with genes that are differentially expressed in different castes, which would be only showed in their phenotype. Hence, virgin queens have genetic values for the queen and worker effects, but they might never actually express these effects. In this simulation virgin queens also obtained phenotypic

values for both of the effects. This is technically incorrect because virgin queens don't express genes for the worker effect at all, and they also do not express the queen effect, not until they become the queen of a colony. We can treat these phenotypic values for virgin queens as values that we could see if these virgin queens would express these traits. We will show later in the Colony value sub-section how we use these traits from different castes. If existence of these phenotypic values for certain castes is a hindrance, we can always remove them for population or colony objects by modifying the corresponding slots as required.

As with the virgin queens, drones also carry DNA with genes that are expressed in different castes. Therefore, drones will also have the queen and workers effect genetic (and phenotypic values) for honey yield even though they do not contribute to this trait in a colony.

```
#>      queenTrait workersTrait
#> [1,]  11.294995   0.16124533
#> [2,]   9.911036   0.22418323
#> [3,]  12.660865   0.05012213
#> [4,]   9.574344   0.10645802
#> [5,]  10.783936   0.15928072
#> [6,]   8.332516   0.13049770
```

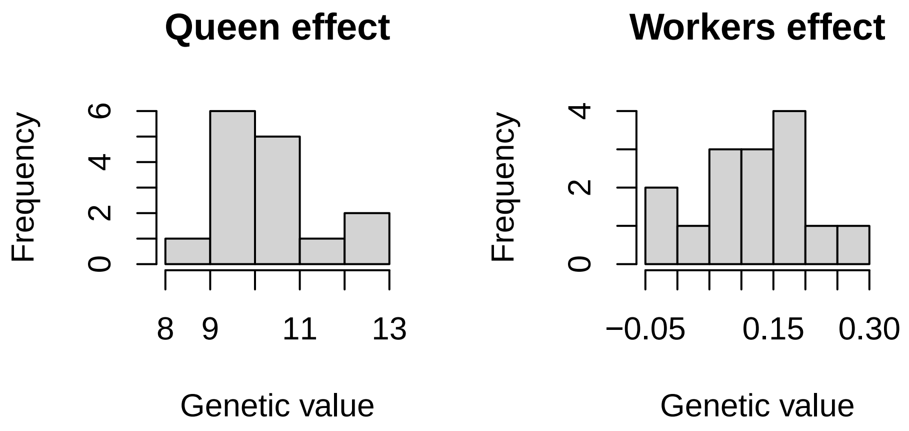

Individual values in a colony

We continue by creating a colony from one base population virgin queen, crossing it, and adding some workers.

```
colony <- createColony(x = basePop[6])
colony <- cross(x = colony, drones = drones)
colony <- addWorkers(x = colony, nInd = 50)
colony
#> An object of class "Colony"
#> Id: 1
#> Location:
#> Queen: 6
#> Number of fathers: 15
#> Number of workers: 50
#> Number of drones: 0
#> Number of virgin queens: 0
#> Has split: FALSE
#> Has swarmed: FALSE
#> Has superseded: FALSE
```

```
#> Has collapsed: FALSE
#> Is productive: FALSE
```

We can access the genetic and phenotypic values of colony members with functions `getGv()` and `getPheno()`, both of which have the `caste` argument (see more via `help(getGv)`).

```
getGv(colony, caste = "queen")
#>   queenTrait workersTrait
#> 6   11.51064   0.06106399
getGv(colony, caste = "workers") |> head(n = 4)
#>    queenTrait workersTrait
#> 36   11.96727   0.12145317
#> 37   11.08759   0.08223230
#> 38   11.22305   0.14560731
#> 39   11.05629   0.06701813

getPheno(colony, caste = "queen")
#>   queenTrait workersTrait
#> 6   10.20933    0.1372256
getPheno(colony, caste = "workers") |> head(n = 4)
#>    queenTrait workersTrait
#> 36  10.224133   0.12546050
#> 37  10.936512  -0.08765655
#> 38  13.793329   0.36517526
#> 39   8.442023  -0.30680413
```

For convenience, there are also alias functions for accessing the genetic and phenotypic values of each caste directly.

```
getQueenGv(colony)
#>   queenTrait workersTrait
#> 6   11.51064   0.06106399
getWorkersGv(colony) |> head(n = 4)
#>    queenTrait workersTrait
#> 36   11.96727   0.12145317
#> 37   11.08759   0.08223230
#> 38   11.22305   0.14560731
#> 39   11.05629   0.06701813

getQueenPheno(colony)
#>   queenTrait workersTrait
#> 6   10.20933    0.1372256
getWorkersPheno(colony) |> head(n = 4)
#>    queenTrait workersTrait
#> 36  10.224133   0.12546050
#> 37  10.936512  -0.08765655
#> 38  13.793329   0.36517526
#> 39   8.442023  -0.30680413
```

Some phenotypes, such as honey yield, are only expressed if colony is at full size. This is achieved by the `buildUp()` colony event function that adds worker and drones and hence turns on the `production` status of the colony (to `TRUE`). SIMplyBee includes a function `isProductive()` to check the production status of a colony.

```r
# Check if colony is productive
isProductive(colony)
#> [1] FALSE

# Build-up the colony and check the production status again
colony <- buildUp(colony)
colony
#> An object of class "Colony"
#> Id: 1
#> Location:
#> Queen: 6
#> Number of fathers: 15
#> Number of workers: 100
#> Number of drones: 100
#> Number of virgin queens: 0
#> Has split: FALSE
#> Has swarmed: FALSE
#> Has superseded: FALSE
#> Has collapsed: FALSE
#> Is productive: TRUE
isProductive(colony)
#> [1] TRUE
```

For the ease of further demonstration, we now combine workers' values into a single data.frame.

```r
# Collate genetic and phenotypic values of workers
df <- data.frame(id = colony@workers@id,
                 mother = colony@workers@mother,
                 father = colony@workers@father,
                 gvQueenTrait = colony@workers@gv[, "queenTrait"],
                 gvWorkersTrait = colony@workers@gv[, "workersTrait"],
                 pvQueenTrait =  colony@workers@pheno[, "queenTrait"],
                 pvWorkersTrait = colony@workers@pheno[, "workersTrait"])
head(df)
#>   id mother father gvQueenTrait gvWorkersTrait pvQueenTrait pvWorkersTrait
#> 1 86      6     21    10.612635     0.17501921     9.143841     0.04454056
#> 2 87      6     34    11.900502     0.09198649    11.178794     0.46639870
#> 3 88      6     35     9.829028     0.02140904     7.622555    -0.18996672
#> 4 89      6     22     9.195027     0.20727214     8.630935     0.42651710
#> 5 90      6     26     9.958134     0.05100661    14.238760     0.26196100
#> 6 91      6     31     9.850228     0.06593323     9.556760     0.05899982
```

To visualise correlation between queen and workers effects in workers, we plot these effect values against each other.

```r
# Covariation between queen and workers effect genetic values in workers
p <- ggplot(data = df, aes(x = gvQueenTrait, y = gvWorkersTrait)) +
  xlab("Genetic value for the queen effect") +
  ylab("Genetic value for the workers effect") +
  geom_point() +
  theme_classic()
print(p)
```

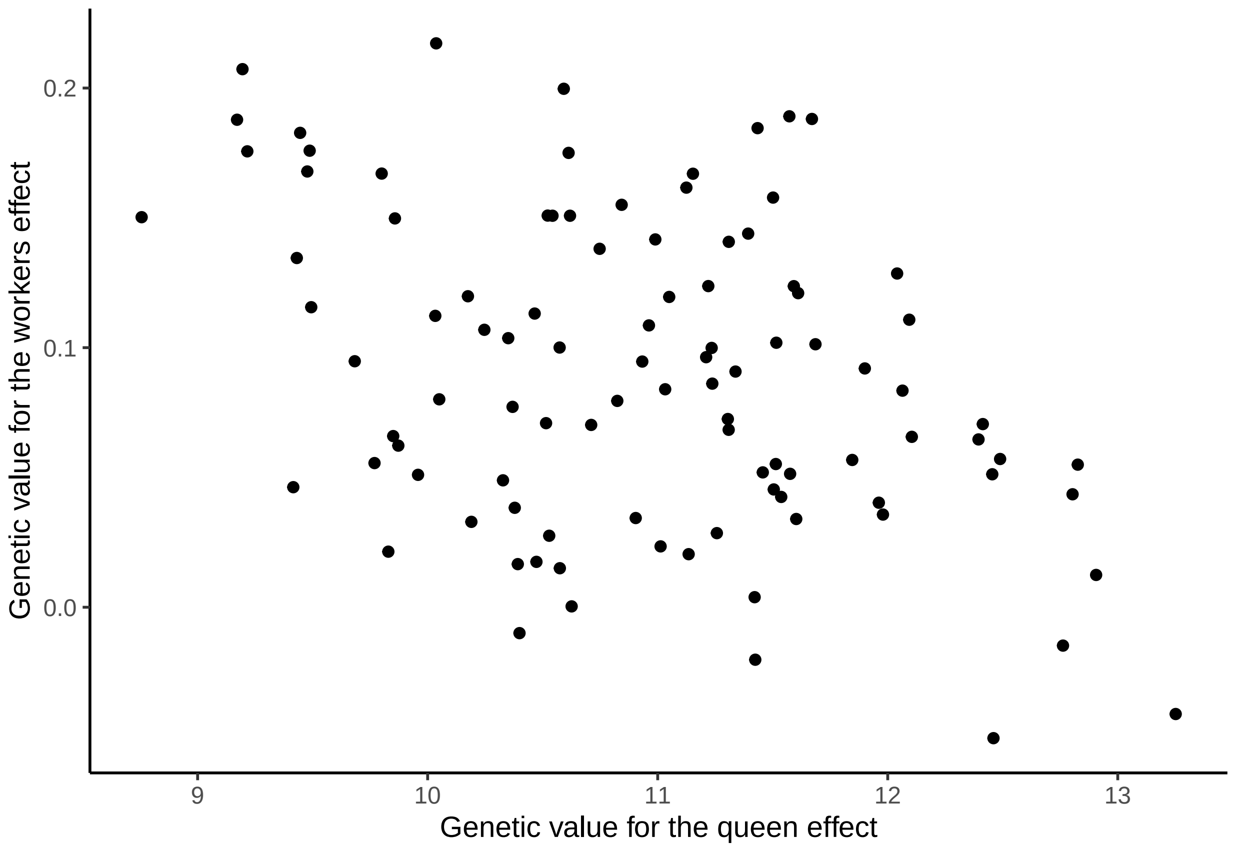

In SIMplyBee, we know genetic values of all individuals, including drones that the queen mated with (=fathers in a colony)!

```
# Variation in patriline genetic values
getFathersGv(colony)
#>    queenTrait workersTrait
#> 21  11.294995   0.16124533
#> 22   9.911036   0.22418323
#> 23  12.660865   0.05012213
#> 24   9.574344   0.10645802
#> 25  10.783936   0.15928072
#> 26   8.332516   0.13049770
#> 27  10.858471   0.15340581
#> 28  10.072441   0.15961340
#> 29  10.143273   0.12600198
#> 30   9.179415   0.07093459
#> 31   9.518660   0.04562114
#> 32   9.933094  -0.01137196
#> 33   9.474274   0.25121686
#> 34  12.703673   0.09517085
#> 35  10.793851  -0.01779449
```

Knowing the father of each worker, we inspect variation in the distribution of genetic values of worker by the patriline (workers from a single father drone) for the workers effect.

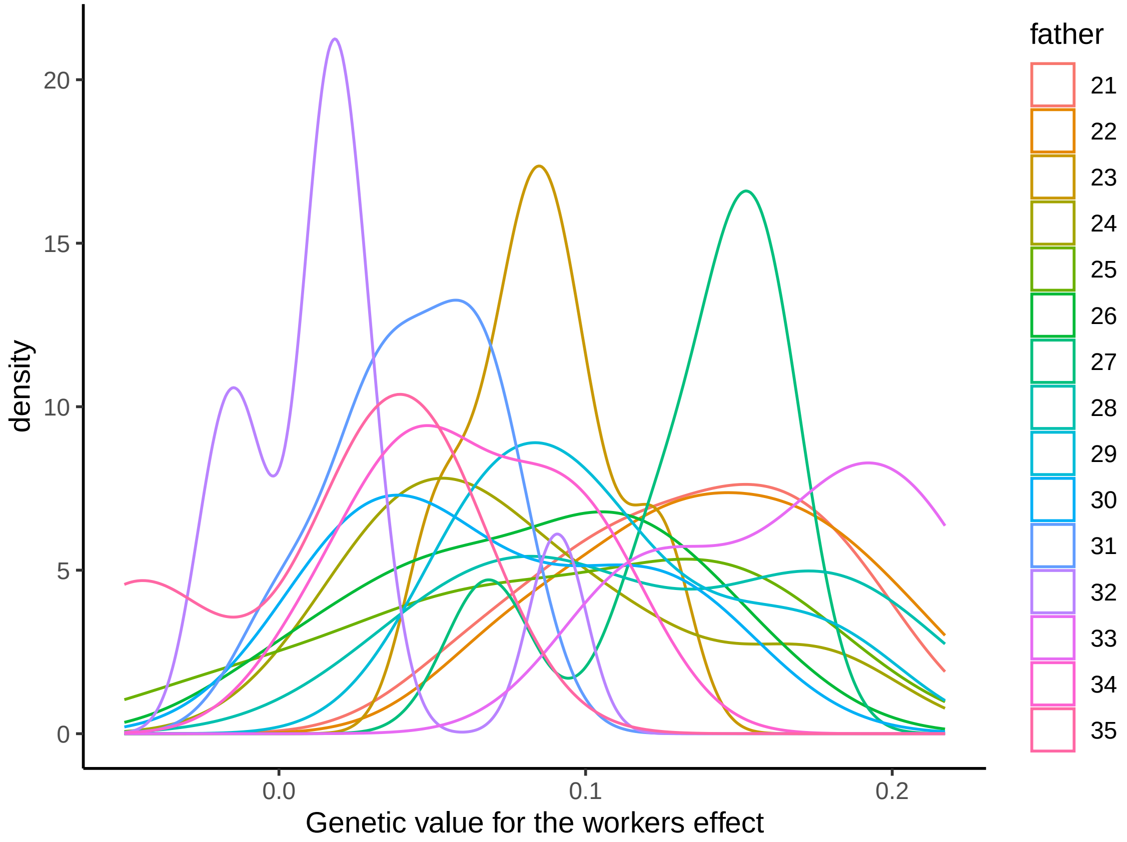

Colony value

However, in honeybees we usually don't observe values on individuals, but on a colony. SIMplyBee provides functions for mapping individual values to a colony value. The general function for this is `calcColonyValue()`, which can combine any value and trait from any caste. There are also aliases `calcColonyGv()` and `calcColonyPheno()`. These functions require users to specify the so-called mapping function (via the `FUN` argument). The mapping function specifies queen and workers traits (potentially also drone traits) and what function we want to apply to each of them before mapping them to the colony value(s). We can also specify whether the colony value(s) depend on the production status. For example, if a colony is not productive, its honey yield would be 0 or unobserved. SIMplyBee provides a general mapping function `mapCasteToColonyValue()` and aliases `mapCasteToColonyGv()` and `mapCasteToColonyPheno()`. These functions have arguments to cater for various situations. By default, they first calculate caste values: leave the queen's value as it is, sum workers' values, potentially sum drones' values, and lastly sum all these caste values together into a colony value. Users can provide their own mapping function(s) too!

We now calculate honey yield for our colony - a single value for the colony.

```
# Colony phenotype value
calcColonyPheno(colony, queenTrait = "queenTrait", workersTrait = "workersTrait")
#>          [,1]
#> [1,] 20.62719
help(calcColonyPheno)
help(mapCasteToColonyPheno)
```

These colony values are not stored in a colony, because they change as colony changes due to various events. For example, reducing the number of workers will reduce the colony honey yield.

```r
# Colony phenotype value from a reduced colony
removeWorkers(colony, p = 0.5) |>
  calcColonyPheno(queenTrait = "queenTrait", workersTrait = "workersTrait")
#>          [,1]
#> [1,] 15.54661
```

Please note that we assumed that the queen contributes half to colony honey yield and workers contribute the other half. This means that removing workers will still give a non-zero honey yield! This shows that we have to design the mapping between individual, caste, and colony values with care!

```r
# Colony phenotype value from a reduced colony
removeWorkers(colony, p = 0.99) |>
  calcColonyPheno(queenTrait = "queenTrait", workersTrait = "workersTrait")
#>          [,1]
#> [1,] 10.25354
```

Finally, note that SIMplyBee currently does not provide functionality for breeding values, dominance deviations, and epistatic deviations at caste and colony levels, despite the availabiliy of AlphaSimR `bv()`, `dd()`, and `aa()` functions. This is because we have to check or develop theory on how to calculate these values across active colonies and hence we currently advise against the use of AlphaSimR `bv()`, `dd()`, and `aa()` functions with SIMplyBee as the output of these functions could be easily misinterpreted.

MultiColony values

The same functions can be used on a `MultiColony` class object. Let's create an apiary.

```r
apiary <- createMultiColony(basePop[7:20])
drones <- createDrones(basePop[1:5], nInd = 100)
droneGroups <- pullDroneGroupsFromDCA(drones, n = nColonies(apiary), nDrones = 15)
apiary <- cross(apiary, droneGroups)
apiary <- buildUp(apiary)
```

We can extract the genetic and phenotypic values from multiple colonies in the same manner as from a single colony, by using `get*Gv()` and `get*Pheno()` functions. The output of these function is a named list with values for each colony or a single matrix if we set the `collapse` argument to `TRUE`.

```r
getQueenGv(apiary) |> head(n = 4)
#> $`2`
#>   queenTrait workersTrait
#> 7   10.49125   0.05584693
#>
#> $`3`
#>   queenTrait workersTrait
#> 8   9.102888   0.03462571
#>
#> $`4`
#>   queenTrait workersTrait
#> 9   10.94144    0.2406293
#>
#> $`5`
#>    queenTrait workersTrait
#> 10    9.47422    0.1703994
getQueenGv(apiary, collapse = TRUE) |> head(n = 4)
#>    queenTrait workersTrait
#> 7   10.491247   0.05584693
#> 8    9.102888   0.03462571
```

```
#> 9   10.941442   0.24062926
#> 10   9.474220   0.17039939
```

In a similar manner, we can calculate colony value for all the colonies in our apiary, where the row names of the output represent colony IDs.

```
colonyGv <- calcColonyGv(apiary)
colonyPheno <- calcColonyPheno(apiary)
data.frame(colonyGv, colonyPheno)
#>     colonyGv colonyPheno
#> 2  17.895104    22.07168
#> 3  10.853636    12.03306
#> 4  26.388667    27.49611
#> 5  23.129439    23.26263
#> 6  20.650031    19.94031
#> 7  24.341961    25.40788
#> 8  19.490592    21.39986
#> 9  27.609208    26.19854
#> 10 10.421110    13.81569
#> 11  9.900976    11.37337
#> 12 18.349305    15.84205
#> 13 21.198780    22.31821
#> 14 21.372387    22.84313
#> 15 17.879690    16.63635
```

Selection on colony values

Since the aim of selection is to select the best individuals or colonies for the reproduction, we could select the best colony in our apiary based on either genetic or phenotypic value for grafting the new generation of virgin queens. We can use the function `selectColonies()` that takes a matrix of colony values (the output of `calcColonyValue()` function). The default behavior is to select the colonies with the highest value (argument `selectTop` set to `TRUE`), but you can also select the colonies with the lowest values (argument `selectTop` set to `FALSE`).

```
# Select the best colony based on gv
selectColonies(apiary, n = 1, by = colonyGv)
#> An object of class "MultiColony"
#> Number of colonies: 1
#> Are empty: 0
#> Are NULL: 0
#> Have split: 0
#> Have swarmed: 0
#> Have superseded: 0
#> Have collapsed: 0
#> Are productive: 0
# Select the best colony based on phenotype
selectColonies(apiary, n = 1, by = colonyPheno)
#> An object of class "MultiColony"
#> Number of colonies: 1
#> Are empty: 0
#> Are NULL: 0
#> Have split: 0
#> Have swarmed: 0
#> Have superseded: 0
#> Have collapsed: 0
```

```
#> Are productive: 0
```

The same functionality is implemented in `pullColonies()` and `removeColonies()`.

Honey yield and Calmness

In this section we expand simulation to two uncorrelated colony traits with queen and workers effects, honey yield and calmness. We follow the same recipe as in the previous section where we simulated only one colony trait.

We first reinitialize the global simulation parameters because we will define new traits. For honey yield we will use the same parameters as before, while for calmness trait we will assume that the trait is scored continuously in such a way that negative values are undesirable and positive values are desirable with zero being population mean. We will further assume the same variances for calmness as for honey yield, and a genetic (and environmental) correlation between the queen and workers effects of -0.4 (and 0.2) for calmness. We assume no genetic or environmental correlation between honey yield and calmness. Beware, this is just an example to show you how to simulate multiple colony traits - we have made up these parameters - please use literature estimates in your simulations!

```
# Global simulation parameters
SP <- SimParamBee$new(founderGenomes)

nQtlPerChr <- 100

# Quantitative genetic parameters - for two traits, each with the queen and workers effects
meanP <- c(10, 10 / SP$nWorkers, 0, 0)
varA <- c(1, 1 / SP$nWorkers, 1, 1 / SP$nWorkers)
corA <- matrix(data = c( 1.0, -0.5,  0.0,  0.0,
                        -0.5,  1.0,  0.0,  0.0,
                         0.0,  0.0,  1.0, -0.4,
                         0.0,  0.0, -0.4,  1.0), nrow = 4, byrow = TRUE)
SP$addTraitA(nQtlPerChr = 100, mean = meanP, var = varA, corA = corA,
             name = c("yieldQueenTrait", "yieldWorkersTrait",
                      "calmQueenTrait", "calmWorkersTrait"))

varE <- c(3, 3 / SP$nWorkers, 3, 3 / SP$nWorkers)
corE <- matrix(data = c(1.0, 0.3, 0.0, 0.0,
                        0.3, 1.0, 0.0, 0.0,
                        0.0, 0.0, 1.0, 0.2,
                        0.0, 0.0, 0.2, 1.0), nrow = 4, byrow = TRUE)
SP$setVarE(varE = varE, corE = corE)
```

We continue by creating a base population of virgin queens and from them an apiary with 10 full-sized colonies.

```
basePop <- createVirginQueens(founderGenomes)
drones <- createDrones(x = basePop[1:5], nInd = 100)
apiary <- createMultiColony(basePop[6:20])
droneGroups <- pullDroneGroupsFromDCA(drones, nColonies(apiary), nDrones = 15)
apiary <- cross(x = apiary, drones = droneGroups)
apiary <- buildUp(apiary)
apiary
#> An object of class "MultiColony"
#> Number of colonies: 15
#> Are empty: 0
```

```
#> Are NULL: 0
#> Have split: 0
#> Have swarmed: 0
#> Have superseded: 0
#> Have collapsed: 0
#> Are productive: 15
```

We can again inspect the genetic (and phenotypic) values of all individuals in each colony and whole apiary with `get*Gv()` and `get*Pheno()` functions. Now, the output contains four traits representing the queen and workers effect for honey yield and calmness. These functions also take an `nInd` argument to sample a number of individuals along with their values.

```
getQueenGv(apiary) |> head(n = 4)
#> $`1`
#>   yieldQueenTrait yieldWorkersTrait calmQueenTrait calmWorkersTrait
#> 6        9.429002         0.1930271      -1.164367      -0.05137121
#>
#> $`2`
#>   yieldQueenTrait yieldWorkersTrait calmQueenTrait calmWorkersTrait
#> 7        10.67997      -0.008152619      -1.229604     -0.001548015
#>
#> $`3`
#>   yieldQueenTrait yieldWorkersTrait calmQueenTrait calmWorkersTrait
#> 8        10.80585      -0.008455044      0.0644797       -0.1667938
#>
#> $`4`
#>   yieldQueenTrait yieldWorkersTrait calmQueenTrait calmWorkersTrait
#> 9          10.465       -0.01892315      0.9477142       -0.1156579
getWorkersPheno(apiary, nInd = 3) |> head(n = 4)
#> $`1`
#>     yieldQueenTrait yieldWorkersTrait calmQueenTrait calmWorkersTrait
#> 521        9.172920         0.0254050      -1.195576       0.08632309
#> 522       10.052913         0.1802971       1.830008       0.04684197
#> 523        8.812305         0.4516191       2.885661      -0.06451385
#>
#> $`2`
#>     yieldQueenTrait yieldWorkersTrait calmQueenTrait calmWorkersTrait
#> 721        8.295013        0.05025980      1.4594951        0.1643924
#> 722        9.925329       -0.02296821      0.9496960        0.1796645
#> 723        8.622603       -0.14106368     -0.7136931        0.1078886
#>
#> $`3`
#>     yieldQueenTrait yieldWorkersTrait calmQueenTrait calmWorkersTrait
#> 921       10.787243       -0.46165117       2.825690       -0.2313329
#> 922        8.332930        0.04672685       2.441930       -0.3308982
#> 923        8.060116        0.08721198      -1.830945       -0.2966755
#>
#> $`4`
#>      yieldQueenTrait yieldWorkersTrait calmQueenTrait calmWorkersTrait
#> 1121        8.320565       0.001691403       1.668994      -0.16186823
#> 1122       10.878568       0.113298416       1.746036       0.07276455
#> 1123       10.061428      -0.199966284       1.709706       0.06685902
```

Now, we calculate colony genetic and phenotypic values for all colonies in the apiary. Since we are simulating

two traits, honey yield and calmness, we have two ways to calculate corresponding colony values. The first way is to use the default `mapCasteToColony*()` function in `calcColony*()` and only define additional arguments as shown here:

```
colonyValues <- calcColonyPheno(apiary,
                                queenTrait = c("yieldQueenTrait", "calmQueenTrait"),
                                workersTrait = c("yieldWorkersTrait", "calmWorkersTrait"),
                                traitName = c("yield", "calmness"),
                                checkProduction = c(TRUE, FALSE)) |> as.data.frame()
colonyValues
#>       yield   calmness
#> 1  22.59316 -1.5038564
#> 2  15.13196 -1.6071599
#> 3  16.13806 -2.2278228
#> 4  18.37984  3.3178979
#> 5  29.24752  7.1683388
#> 6  19.03224  0.4105692
#> 7  20.29766  0.7069055
#> 8  12.55385  5.3188263
#> 9  24.26366  2.4279099
#> 10 28.11467  0.6569555
#> 11 14.66707 -0.7321977
#> 12 17.71359  1.4703554
#> 13 21.61985  1.7344341
#> 14 23.41407  2.8555588
#> 15 18.10520  2.5277883
```

The second way is to create our own mapping function. An equivalent outcome to the above is shown below just to demonstrate use of your own function, but we are simply just reusing `mapCasteToColonyPheno()` twice;)

```
myMapCasteToColonyPheno <- function(colony) {
  yield <- mapCasteToColonyPheno(colony,
                                 queenTrait = "yieldQueenTrait",
                                 workersTrait = "yieldWorkersTrait",
                                 traitName = "yield",
                                 checkProduction = TRUE)
  calmness <- mapCasteToColonyPheno(colony,
                                    queenTrait = "calmQueenTrait",
                                    workersTrait = "calmWorkersTrait",
                                    traitName = "calmness",
                                    checkProduction = FALSE)
  return(cbind(yield, calmness))
}
colonyValues <- calcColonyPheno(apiary, FUN = myMapCasteToColonyPheno) |> as.data.frame()
colonyValues
#>       yield   calmness
#> 1  22.59316 -1.5038564
#> 2  15.13196 -1.6071599
#> 3  16.13806 -2.2278228
#> 4  18.37984  3.3178979
#> 5  29.24752  7.1683388
#> 6  19.03224  0.4105692
#> 7  20.29766  0.7069055
#> 8  12.55385  5.3188263
```

```
#> 9  24.26366  2.4279099
#> 10 28.11467  0.6569555
#> 11 14.66707 -0.7321977
#> 12 17.71359  1.4703554
#> 13 21.61985  1.7344341
#> 14 23.41407  2.8555588
#> 15 18.10520  2.5277883
```

Again, we can now select the best colony based on the best phenotypic value for either yield, calmness, or an index of both. Let's say that both traits are equally important so we select on a weighted sum of both of them - we will use the AlphaSimR `selIndex()` function that enables this calculation along with scaling. We will represent the index such that it has a mean of 100 and standard deviation of 10 units.

```
colonyValues$Index <- selIndex(Y = colonyValues, b = c(0.5, 0.5), scale = TRUE) * 10 + 100
bestColony <- selectColonies(apiary, n = 1, by = colonyValues$Index)
getId(bestColony)
#> [1] 5
```

We see that we selected colony with ID "4", but we would be selecting a different colony based on different selection criteria (yield, calmness, or index).

Strength and honey yield

In this section we change simulation to two traits where the phenotype realisation of the first trait affects the phenotype realisation of the second trait. Specifically, we will assume that queen's fecundity, and hence the number of workers, is under the genetic affect of the queen and her environment. Furthermore, we will assume as before that colony honey yield is due to the queen effect and workers effect. Since the value of the workers effect depends on then number of workers, we obtain correlation between fecundity and honey yield, even if these traits would be uncorrelated on the queen level. We emphasise that this is just an example and the biology of these traits might be different.

We follow the same logic as before and simulate three traits that will contribute to two colony traits, queen's fecundity, that is colony strength, and honey yield. We assume that fecundity is only due to the queen (and not the workers), hence we simulate only the queen effect for this trait. For honey yield we again assume that both the queen and workers contribute to the colony value. For speed of simulation we only simulate 100 workers per colony on average and split honey yield mean between the queen and workers. We measure fecundity with the number of workers, which is a count variable and for such variables Poisson distribution is a good model. This distribution has just one parameter (lambda) that represents both the mean and variance of the variable. To this end we set phenotypic variance to 100 and split it into 25 for genetic and 65 for environmental variance. As before we warn that these are just exemplary values to demonstrate the code functionality and do not necessarily reflect published values!

```
# Global simulation parameters
SP <- SimParamBee$new(founderGenomes)

# Quantitative genetic parameters
# - the first trait has only the queen effect
# - the second trait has both the queen and workers effects
nWorkers <- 100
mean <- c(nWorkers, 10, 10 / nWorkers)
varA <- c(25, 1, 1 / nWorkers)
corA <- matrix(data = c(1.0,  0.0,  0.0,
                        0.0,  1.0, -0.5,
                        0.0, -0.5,  1.0), nrow = 3, byrow = TRUE)
SP$addTraitA(nQtlPerChr = 100, mean = mean, var = varA, corA = corA,
```

```r
             name = c("fecundityQueenTrait", "yieldQueenTrait", "yieldWorkersTrait"))

varE <- c(75, 3, 3 / nWorkers)
corE <- matrix(data = c(1.0, 0.0, 0.0,
                        0.0, 1.0, 0.3,
                        0.0, 0.3, 1.0), nrow = 3, byrow = TRUE)
SP$setVarE(varE = varE, corE = corE)
```

We continue by creating an apiary with 10 colonies.

```r
basePop <- createVirginQueens(founderGenomes)
drones <- createDrones(x = basePop[1:5], nInd = 100)
apiary <- createMultiColony(basePop[6:20])
droneGroups <- pullDroneGroupsFromDCA(drones, nColonies(apiary), nDrones = 15)
apiary <- cross(x = apiary, drones = droneGroups)
```

Let's explore queen's genetic and phenotypic values for fecundity and honey yield. The below printouts show quite some variation in fecundity between queens at the genetic, but particularly phenotypic level. This is a small example, so we should not put too much into correlations between these three variables. However, if you restart this simulation many times, you will notice zero correlation on average between `fecundityQueenTrait` and the other two traits and negative correlation on average between `yieldQueenTrait` and `yieldWorkersTrait`. Just like we defined in the global simulation parameters.

```
#>    fecundityQueenTrait yieldQueenTrait yieldWorkersTrait
#> 6             94.35780        9.908602       0.079324398
#> 7            107.68910       10.370199       0.117254186
#> 8             92.58610        9.600060       0.139859682
#> 9            110.64831        9.690306      -0.039407344
#> 10           102.57312        9.575998       0.307095764
#> 11           100.80151        9.019673       0.217847980
#> 12            95.93794        9.147415       0.187296763
#> 13           103.03970        8.528660       0.006875713
#> 14           102.29582       10.234678       0.004493492
#> 15           103.42816       12.019470      -0.001030017
#> 16            98.38597        9.998500       0.196051729
#> 17           101.69519        9.236933       0.220384214
#> 18           100.14803       10.988505      -0.020804160
#> 19           105.48786       10.422964       0.062179185
#> 20            97.68019        9.582720       0.098696649
#>                     fecundityQueenTrait yieldQueenTrait yieldWorkersTrait
#> fecundityQueenTrait          1.00000000      0.07524596        0.29323207
#> yieldQueenTrait              0.07524596      1.00000000       -0.09329096
#> yieldWorkersTrait            0.29323207     -0.09329096        1.00000000
```

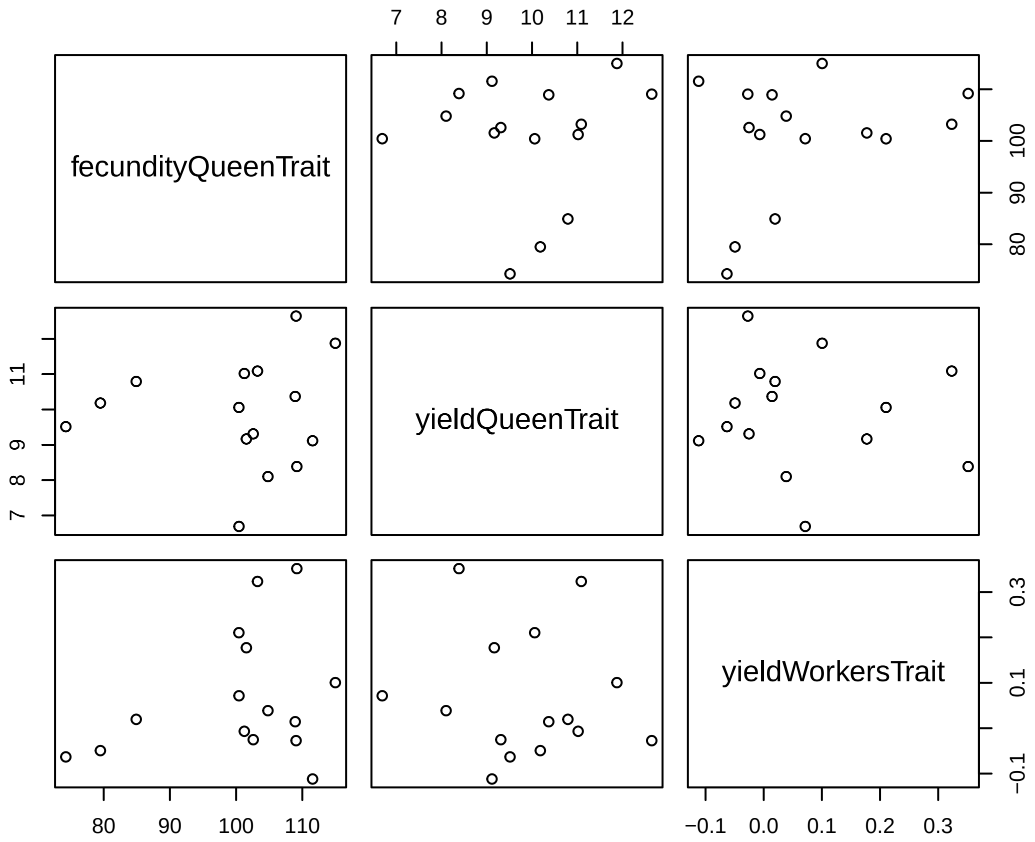

We next build-up colonies in the apiary. But instead of building them all up to the same fixed number of workers, we build them up according to queen's fecundity. For that we use the sampling function `nWorkersColonyPhenotype()`, that samples the number of workers based on phenotypes of colony members, in our case `fecundityQueenTrait` in queens. Correspondingly, each colony will have a different number of workers. Read more about this function in it's help page.

```
apiary <- buildUp(apiary, nWorkers = nWorkersColonyPhenotype,
                  queenTrait = "fecundityQueenTrait")
cbind(nWorkers = nWorkers(apiary), queenPheno)
#>    nWorkers fecundityQueenTrait yieldQueenTrait yieldWorkersTrait
#> 1       102           101.55298        9.166005       0.177260795
#> 2       100           100.42068       10.059037       0.210374126
#> 3        72            74.27704        9.513945      -0.063207025
#> 4       115           114.98931       11.876174       0.100460603
#> 5        96           100.43477        6.689929       0.071426574
#> 6       103           103.23273       11.088830       0.323306874
#> 7        76            79.49918       10.183634      -0.049334688
#> 8       101           101.24008       11.019174      -0.006782074
#> 9       111           111.55377        9.114977      -0.111875424
#> 10       97           109.05807       12.645872      -0.027390859
#> 11      105           104.80820        8.100968       0.038676430
#> 12      109           109.17380        8.385500       0.351557551
#> 13       85            84.91033       10.792932       0.019515970
#> 14      109           108.92612       10.369582       0.014326039
```

```
#> 15       103             102.58797        9.310875       -0.025272510
help(nWorkersColonyPhenotype)
```

To compute the colony value for honey yield, we again employ the `calcColonyPheno()` function. Correlating the queen and colony values we will now see a positive correlation because our individual to colony mapping function sums workers effect across all workers and the more workers there are the larger the sum.

```
#>                         nWorkers fecundityQueenTrait yieldQueenTrait
#> nWorkers             1.000000000          0.96469902    -0.003490279
#> fecundityQueenTrait  0.964699018          1.00000000     0.075245958
#> yieldQueenTrait     -0.003490279          0.07524596     1.000000000
#> yieldWorkersTrait    0.353989883          0.29323207    -0.093290958
#> yield                0.021211503          0.03435343    -0.527908759
#>                     yieldWorkersTrait       yield
#> nWorkers                   0.35398988  0.02121150
#> fecundityQueenTrait        0.29323207  0.03435343
#> yieldQueenTrait           -0.09329096 -0.52790876
#> yieldWorkersTrait          1.00000000  0.53205727
#> yield                      0.53205727  1.00000000
```

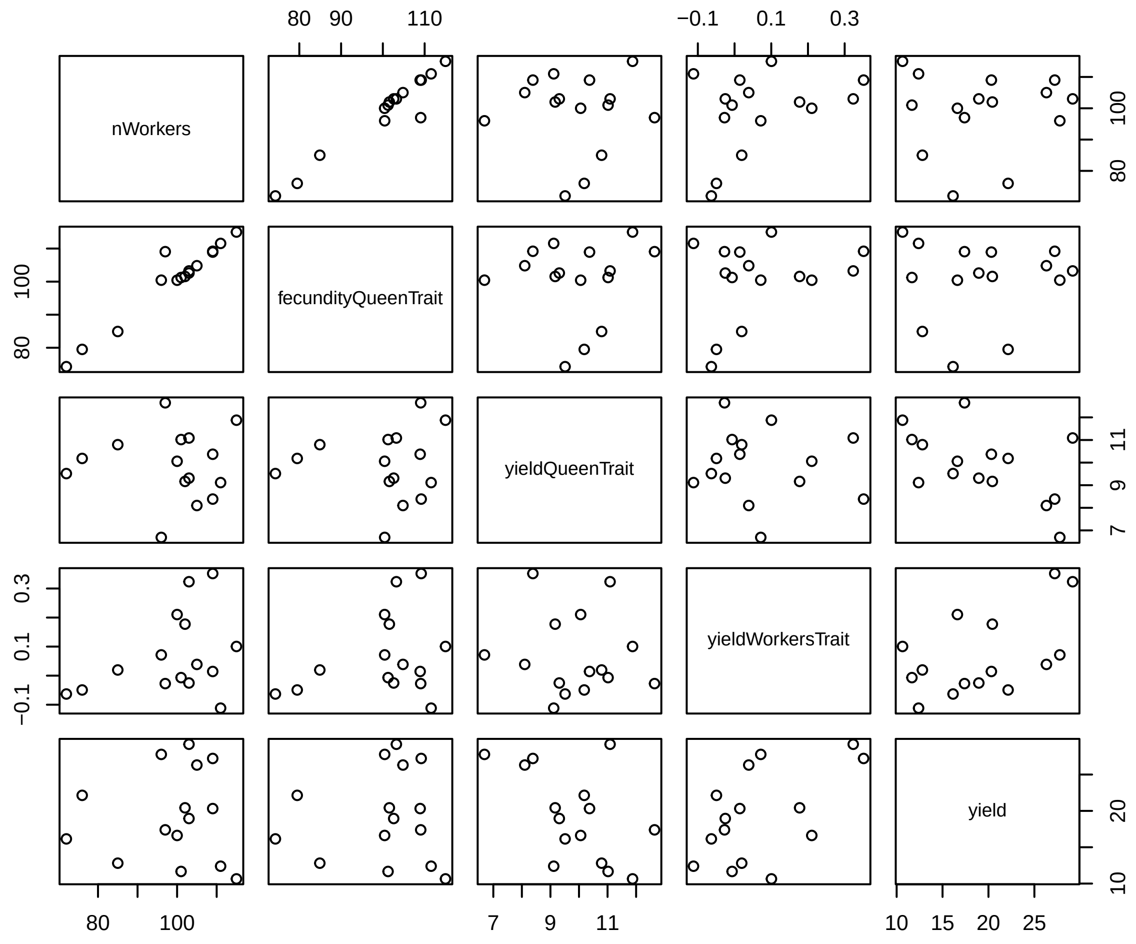

Supplement: Supplementary file 6 — Additional file 6. Quantitative genetics vignette. This vignette describes and demonstrates how SIMplyBeeimplements quantitative genetics principles for honeybees. Specifically, itdescribes three different examples where we simulate a single colony trait,two colony traits, and two colony traits where one trait impacts the otherone via the number of workers. This vignette can also be found on https://cran.r-project.org/package=SIMplyBee and http://www.SIMplyBee.info. [file 12711_2023_798_MOESM6_ESM.pdf]
