# Supplementary material for: SIMplyBee: an R package to simulate honeybee populations and breeding programs
Source: Genet Sel Evol. 2023 May 9;55:31. doi: 10.1186/s12711-023-00798-y (PMC10169377; doi:10.1186/s12711-023-00798-y)
Supplement: Supplementary file 7 — Additional file 7. Sampling functions vignette. This vignette introduces sampling functions that sample eitherthe number of caste individuals or the proportion of workers that stay orare removed in colony events. This vignette can also be found on https://cran.r-project.org/package=SIMplyBee and http://www.SIMplyBee.info. [file 12711_2023_798_MOESM7_ESM.pdf]

Additional file 7 - Sampling functions vignette

2023-03-21

Introduction

SIMplyBee includes functions to sample various values that are expected to vary between colonies and events. These functions are used to sample numbers, usually individuals, and proportions. We can use the functions, pass them to other functions, or save them in the `SimParamBee` object so they can be used by default by other functions.

We start by loading the package:

```
library(package = "SIMplyBee")
#> Loading required package: AlphaSimR
#> Loading required package: R6
#>
#> Attaching package: 'SIMplyBee'
#> The following object is masked from 'package:base':
#>
#>     split
```

Functions to sample numbers

First, there are functions to sample the number of caste individuals from either a Poisson or truncated Poisson distribution: `n*Poisson()` and `n*TruncPoisson()`, where `*` is either `Workers`, `Drones`, `VirginQueens`, or `Fathers`. Most SIMplyBee functions that take the number of individuals as an argument can accept these sampling functions as an input, meaning that the output of such function calls will be stochastic. These functions are useful when you want to sample a variable number of individuals around a mean, as for example when mating virgin queens with a variable number of drones.

Let's start a simulation by creating a DCA and an apiary with 10 virgin colonies:

```
founderGenomes <- quickHaplo(nInd = 20, nChr = 1, segSites = 100)
SP <- SimParamBee$new(founderGenomes)
basePop <- createVirginQueens(founderGenomes)

# Create a DCA from the first 10 base virgin queens
DCA <- createDrones(x = basePop[1:10], nInd = 100)

# Create an apiary with 10 virgin colonies
apiary <- createMultiColony(basePop[11:20])
```

From the literature we know that virgin queens on average mate to 17 drones, but the actual number varies around this mean. Some mate with 10 drones, some with 20, etc. To resemble this variation, we can use the function `nFathersPoisson()` to sample variable number of drones from a DCA. The default average for this function is 15, but you can use any value you want. Let's use this function to sample 1,000 values and inspect the distribution and the mean.

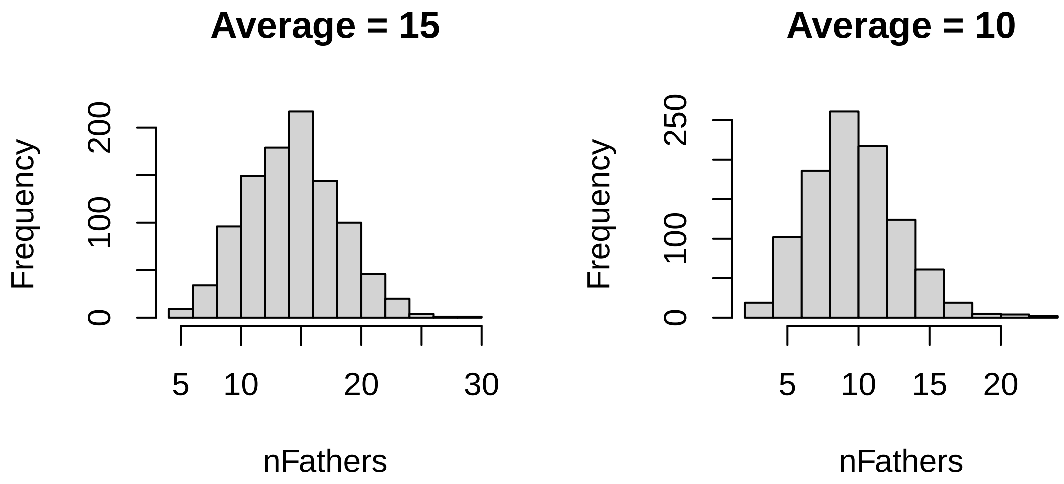

Let's now use this functionality to sample a variable number of drones from the DCA to mate with each of the 10 virgin queens.

```
droneGroups <- pullDroneGroupsFromDCA(DCA = DCA, n = 10, nDrones = nFathersPoisson)
apiary <- cross(apiary, drones = droneGroups)
```

And inspect the number of fathers in each of the colony and their mean.

```
nFathers(apiary)
#>  1  2  3  4  5  6  7  8  9 10
#> 12  9 13 22 11 17 13 16 18 19
mean(nFathers(apiary))
#> [1] 15
```

Second, we have a group of functions that will sample the number of individuals according to the colony phenotype, whatever that might be. These functions are named `n*ColonyPhenotype()`, where `*` is either is either `Workers`, `Drones`, or `VirginQueens`. An example of this would be sampling the number of workers and drones according to queen's fecundity or honey yield. An example of this can be seen in the quantitative genetics vignette in the "Strength and honey yield" example.

Functions that sample proportions

SIMplyBee also includes functions to sample the proportions of workers that leave or are removed when downsizing, splitting, or swarming a colony from either a uniform distribution or from a beta distribution that accounts for the number of individuals in a colony (colony strength). These functions are named `*PUnif()`, where `*` can be either `swarm`, `split`, or `downsize`. There is an additional function, `splitPColonyStrength()`, that determines the number of workers to be removed in a split according to the colony strength.

Let's say we want to swarm all the colonies in our apiary with a variable percentage of workers that leave. We want to sample this percentage from an uniform distribution with the mean of 0.6. For this, we use `swarmPUnif()` function that takes a `min` and a `max` values and sample a value between them. By default, the `min` is set to 0.4 and `max` to 0.6. Let's use this function to sample a 1,000 values between 0.5 and 0.7 and inspect the mean.

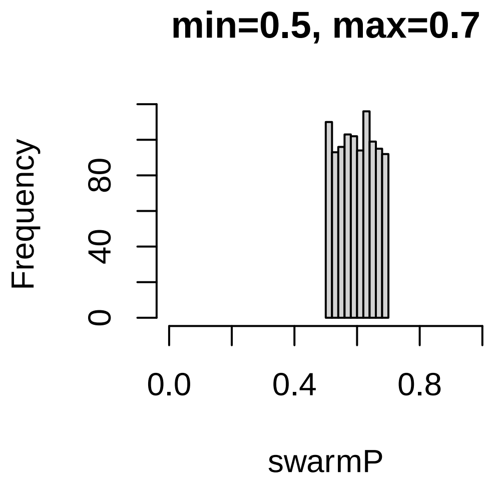

Let's now swarm all the colonies in our apiary with a variable percentage of workers that leave.

```
apiary <- buildUp(apiary, nWorkers = 1000, nDrones = 100)
tmp <- swarm(apiary, p = swarmPUnif(n = 10, min = 0.5, max = 0.7))
nWorkers(tmp$swarm)
#>  11  13  15  17  19  21  23  25  27  29
#> 579 673 666 697 516 555 512 601 555 602
```

We see that each colony swarmed with a different percentage between 0.5 and 0.7.
