# Supplementary material for: SIMplyBee: an R package to simulate honeybee populations and breeding programs
Source: Genet Sel Evol. 2023 May 9;55:31. doi: 10.1186/s12711-023-00798-y (PMC10169377; doi:10.1186/s12711-023-00798-y)
Supplement: Supplementary file 8 — Additional file 8. Computing time. The table shows the mean computing time for basicSIMplyBee functions of ten replicates. It shows the time to create ten or amillion drones; create ten or a thousand empty or virgin colonies; to crossten or a thousand colonies by providing n drone populations, where n is the number of virgin queens, or by providing a singledrone population and a cross plan; and to build-up ten or a thousandcolonies to a thousand or 60 thousand workers. [file 12711_2023_798_MOESM8_ESM.pdf]

	Command	Number	Type	Time[s]	Time[min]
1	createDrones	10	-	0.02	0.00
2	createDrones	1mio	-	11.59	0.19
3	createMultiColony	10	empty	0.00	0.00
4	createMultiColony	1000	empty	0.00	0.00
5	createMultiColony	10	virgin	3.62	0.06
6	createMultiColony	1000	virgin	251.13	4.19
7	cross	10	drone packages	33.66	0.56
8	cross	1000	drone packages	2822.52	47.04
9	cross	1000	cross plan	1241.07	20.68
10	buildUp	10	1K workers	2.15	0.04
11	buildUp	10	60K workers	18.33	0.31
12	buildUp	1000	10K workers	3.64	0.06
13	buildUp	1000	60K workers	19.89	0.33
